# Increasing histone acetylation improves sociability and restores learning and memory in *KAT6B*-haploinsufficient mice

**DOI:** 10.1172/JCI167672

**Published:** 2024-04-01

**Authors:** Maria I. Bergamasco, Hannah K. Vanyai, Alexandra L. Garnham, Niall D. Geoghegan, Adam P. Vogel, Samantha Eccles, Kelly L. Rogers, Gordon K. Smyth, Marnie E. Blewitt, Anthony J. Hannan, Tim Thomas, Anne K. Voss

**Affiliations:** 1 The Walter and Eliza Hall Institute of Medical Research, Melbourne, Victoria, Australia.; 2 Department of Medical Biology and; 3 Centre for Neurosciences of Speech, University of Melbourne, Parkville, Victoria, Australia.; 4 Redenlab Inc., Melbourne, Australia.; 5 School of Mathematics and Statistics, University of Melbourne, Parkville, Victoria, Australia.; 6 Florey Institute of Neuroscience and Mental Health, Melbourne, Victoria, Australia.; 7 Department of Anatomy and Physiology, University of Melbourne, Parkville, Victoria, Australia.

**Keywords:** Development, Genetics, Epigenetics, Genetic diseases, Neurodevelopment

## Abstract

Mutations in genes encoding chromatin modifiers are enriched among mutations causing intellectual disability. The continuing development of the brain postnatally, coupled with the inherent reversibility of chromatin modifications, may afford an opportunity for therapeutic intervention following a genetic diagnosis. Development of treatments requires an understanding of protein function and models of the disease. Here, we provide a mouse model of Say-Barber-Biesecker-Young-Simpson syndrome (SBBYSS) (OMIM 603736) and demonstrate proof-of-principle efficacy of postnatal treatment. SBBYSS results from heterozygous mutations in the *KAT6B* (*MYST4/MORF/QFK*) gene and is characterized by intellectual disability and autism-like behaviors. Using human cells carrying SBBYSS-specific *KAT6B* mutations and *Kat6b* heterozygous mice (*Kat6b^+/–^*), we showed that KAT6B deficiency caused a reduction in histone H3 lysine 9 acetylation. *Kat6b^+/–^* mice displayed learning, memory, and social deficits, mirroring SBBYSS individuals. Treatment with a histone deacetylase inhibitor, valproic acid, or an acetyl donor, acetyl-carnitine (ALCAR), elevated histone acetylation levels in the human cells with SBBYSS mutations and in brain and blood cells of *Kat6b^+/–^* mice and partially reversed gene expression changes in *Kat6b^+/–^* cortical neurons. Both compounds improved sociability in *Kat6b^+/–^* mice, and ALCAR treatment restored learning and memory. These data suggest that a subset of SBBYSS individuals may benefit from postnatal therapeutic interventions.

## Abstract 2

Treatment with a histone deacetylase inhibitor or an acetyl donor shows proof-of-principal efficacy in a mouse model of the cognitive disorder Say-Barber-Biesecker-Young-Simpson syndrome.

## Graphical abstract

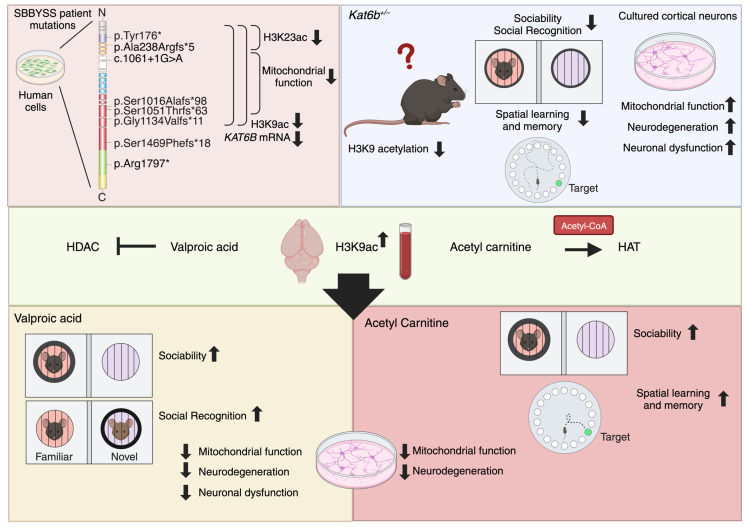

## Introduction

Heterozygous mutations in the gene encoding the MYST family histone acetyltransferase, KAT6B, underlie 2 main intellectual disability disorders: the Say-Barber-Biesecker-Young-Simpson variant of Ohdo syndrome (SBBYSS) (OMIM 603736) (1) and genitopatellar syndrome (GPS) (OMIM 606170) (2). Traits common to both syndromes include a global developmental delay, intellectual disability, autistic-like behaviors, hypotonia, congenital heart defects, and hearing loss (3). GPS is distinguished by hypoplastic or absent patellae, flexion contractures of the hips and knees, agenesis of the corpus callosum, microcephaly, craniofacial dysmorphisms, and genitourinary abnormalities (2). SBBYSS is characterized by blepharophimosis, ptosis, a mask-like face, and long first digits (1).

While 80% (17/21) of GPS variants are in the last coding exon of *KAT6B*, 50% (27/54) of SBBYSS variants are located in more proximal exons. More proximal variants are associated with a milder phenotype and are proposed to result in nonsense-mediated decay of the mutant transcript and *KAT6B* haploinsufficiency (1, 4). In contrast, variants in the final or penultimate exon of *KAT6B* are hypothesized to generate transcripts that escape nonsense-mediated decay and aberrant protein with dominant-negative effects or an abnormal function (4, 5). Notably, these hypotheses remain to be validated by functionally assessing mutant KAT6B proteins.

The *Kat6b* gene is expressed highly within the developing mouse brain (6) and in the adult subventricular zone (7, 8), where it regulates adult neurogenesis (8, 9). Mice deficient in *Kat6b* exhibit reduced proliferation in the ventricular zone of the telencephalon, a reduction in the size of the cortical plate during development, and a decrease in cortical layer V pyramidal neurons and cortical interneurons in adulthood (6). Adult mice deficient in *Kat6b* have fewer neural stem cells (NSCs), and these show impaired self-renewal and reduced neuronal differentiation in vitro (8).

Histone acetyltransferases function by transferring an acetyl-group from acetyl–coenzyme A to the ε-amino group of lysine residues on histone proteins (10). This function is opposed by histone deacetylases (HDACs). The histone acetyltransferase domain of KAT6B can acetylate the core histone proteins H2A, H2B, H3, and H4 as well as the linker histone H1 in cell-free acetylation assays (6). In comparison, in human small-cell lung cancer cells (11) and myelogenous leukemia cells (12) KAT6B is thought to acetylate histone H3 on lysine 23 (H3K23ac) specifically. A comprehensive analysis of the histone lysine targets of KAT6B across a range of cell types has not been conducted.

To investigate the consequences of individual SBBYSS-causing variants on *KAT6B* mRNA levels, histone acetylation, and mitochondrial function, we generated a panel of clonal human cell lines, each carrying a specific SBBYSS mutation. In parallel, we generated a mouse model of *Kat6b* heterozygosity to determine the effects on histone acetylation and mouse behavior, learning, and memory and how these parameters were affected by treatments that increase histone acetylation.

## Results

### Reduced KAT6B mRNA and H3K9ac in human cells carrying specific SBBYSS mutations.

**Figure 1:**
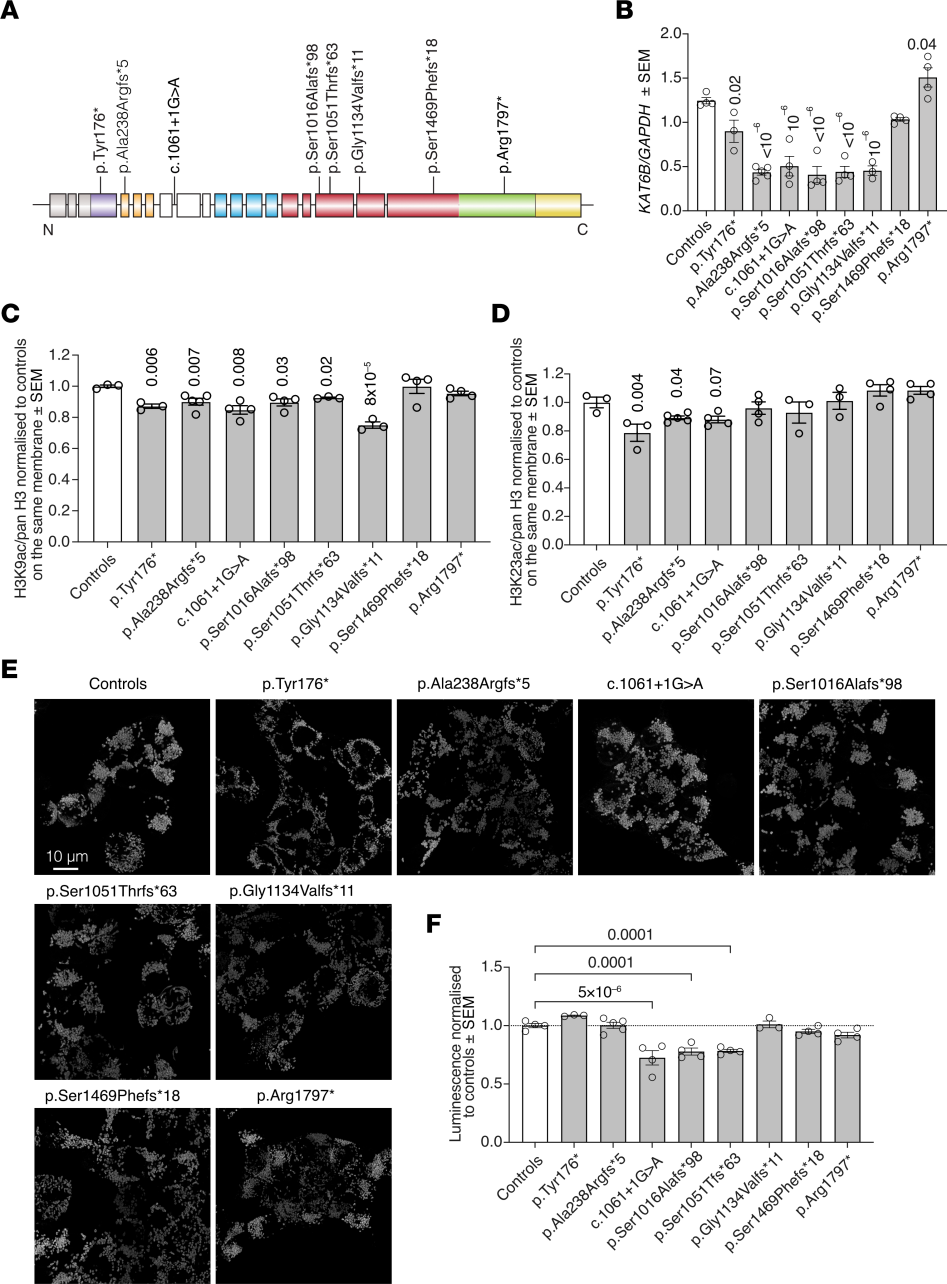
mRNA, H3K9ac, and H3K23ac levels and mitochondrial function in HEK293T cells carrying SBBYSS-specific *KAT6B* mutations. (**A**) Diagram of SBBYSS mutations analyzed. Colors indicate protein-coding domains; boxes indicate individual *KAT6B* exons. Gray, 5′ UTR; purple, conserved N-terminal domain; orange, tandem PHD fingers; blue, MYST histone acetyltransferase domain; red, acidic region; green and yellow, serine- and methionine-rich regions. (**B**) *KAT6B* mRNA levels normalized to *GAPDH* in control and HEK293T cells carrying SBBYSS-causing mutation assessed by quantitative reverse-transcriptase PCR (qRT-PCR). (**C** and **D**) Quantitation of H3K9ac (**C**) or H3K23ac (**D**) levels normalized to pan H3 in HEK293T cells carrying SBBYSS-causing mutations assessed on Western blots shown in Supplemental Figures 1 and 2. (**E**) Representative maximal projection confocal microscopy images of control and HEK293T cells carrying SBBYSS-causing mutations stained with MitoTracker. Scale bar: 10 μm. (**F**) ATP levels assessed by Mitochondrial ToxGlo assay in control and HEK293T cells carrying SBBYSS-causing mutations normalized to controls. *n =* 3–5 individual clonal HEK293T cell lines per SBBYSS mutation. Data are represented as mean ± SEM and were analyzed by 1-way ANOVA with Holm-Šidák correction for multiple testing (**B**–**D** and **F**). *P* values for each mutation are shown above each bar. Circles, individual clonal cell lines. Related data in Supplemental Figures 1–4.

Mutations underlying SBBYSS could result in nonsense-mediated decay of the mutant transcript and loss of KAT6B function (1, 4). To investigate the consequences of individual SBBYSS mutations on *KAT6B* transcript abundance and histone acetylation, we used CRISPR/Cas9 genome editing and homology-directed repair to generate clonal HEK293T cell lines carrying specific SBBYSS mutations spanning the *KAT6B* gene, validated by sequencing (Figure 1A and Supplemental Tables 1 and 2; supplemental material available online with this article; https://doi.org/10.1172/JCI167672DS1). HEK293T cells were chosen as a robust cell type, amenable to CRISPR/Cas9 manipulation and clonal cell isolation.

Relative to control cells, a 25%–66% reduction in *KAT6B* mRNA levels in cells with SBBYSS mutations in the first to the penultimate coding exon was observed, whereas *KAT6B* mRNA levels were unaffected or even elevated in cells with mutations in the final exon (*P <* 10^–6^ to 0.04; Figure 1, A and B).

We assessed acetylation at H3K14 and H3K23, previously proposed acetylation targets of KAT6B (11–13), as well as H3K9ac. Compared with control cells, we observed a reduction in H3K9ac of up to 25% in cells with SBBYSS mutations in the first to the penultimate coding exon, whereas global H3K9ac was unaffected by mutations in the final exon (*P =* 8 × 10^–5^ to 0.03; Figure 1C and Supplemental Figure 1). In addition, we found a reduction of up to 20% in H3K23ac in cells with the 2 most N-terminal *KAT6B* mutations (*P =* 0.004 and 0.04, respectively; Figure 1D and Supplemental Figure 2). Interestingly, H3K14ac was increased in *KAT6B* mutant cell lines with reduced H3K9ac levels (*P =* 0.001 to 0.048; Supplemental Figure 3).

As KAT6B has been implicated in mitochondrial function in Alzheimer’s disease (14) and aspects of the KAT6B-related KAT6A syndrome have been proposed to arise from mitochondrial dysfunction (15), we assessed mitochondrial morphology and function across SBBYSS HEK293T cell lines. Cells were grown in galactose to promote oxidative phosphorylation over glycolysis. Mitochondrial morphology did not differ between cell genotypes (Figure 1E and Supplemental Figure 4, A and B). Cells with 3 different mutations in the central part of the protein displayed a reduced ATP output compared with controls in galactose medium (*P =* 5 × 10^–6^ to 0.0001; Figure 1F), but not in glucose medium (Supplemental Figure 4C).

### Kat6b^+/–^ heterozygous and Kat6b^–/–^ homozygous mutant mice.

**Figure 2:**
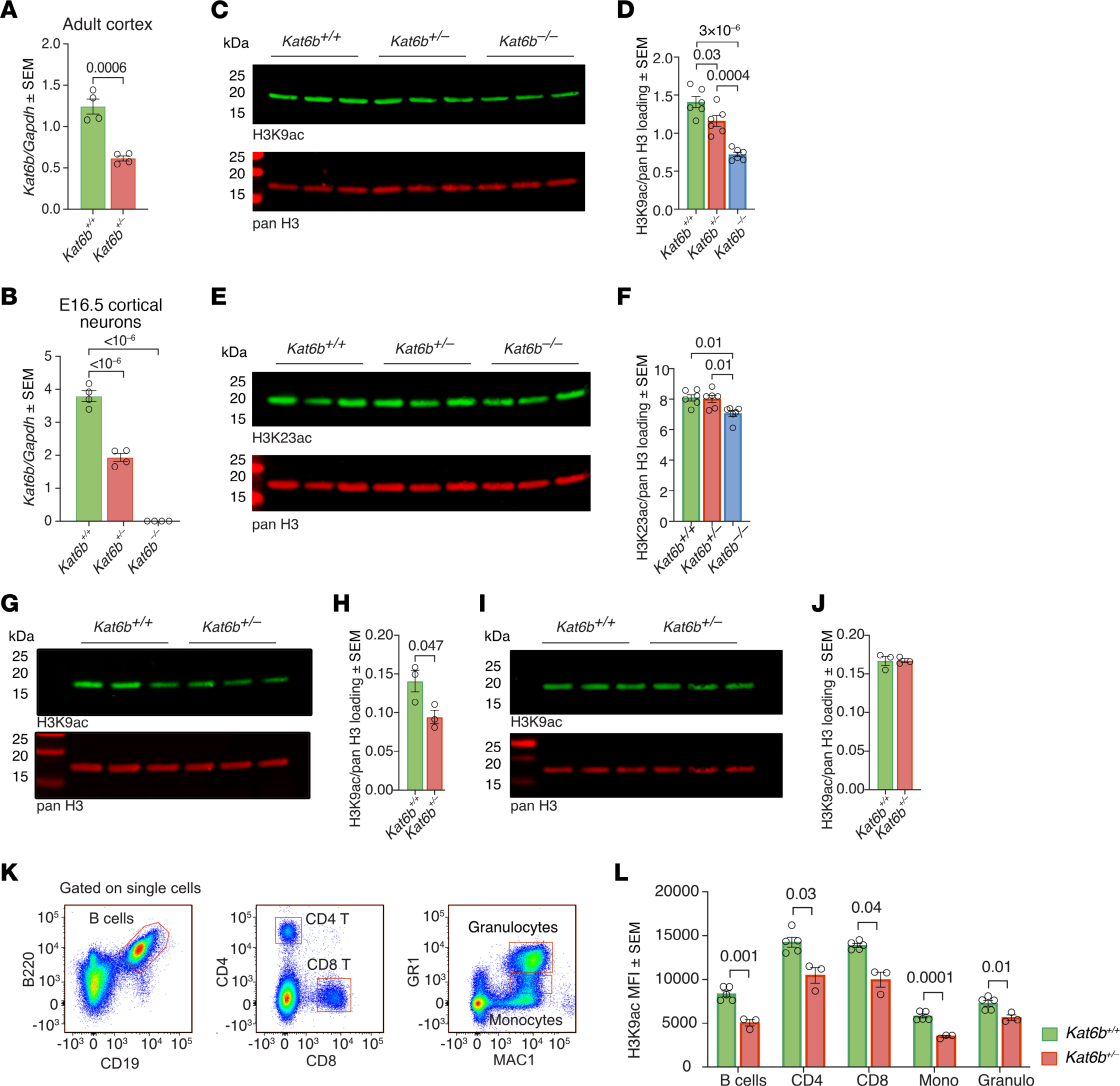
mRNA abundance, H3K9ac and H3K23ac in *Kat6b* mutant mice. (**A** and **B**) *Kat6b* mRNA levels normalized to *Gapdh* assessed by RT-qPCR in *Kat6b^+/+^* and *Kat6b^+/–^* adult cortex (**A**) or *Kat6b^+/+^*, *Kat6b^+/–^*, and *Kat6b^–/–^* E16.5 cortical neurons (**B**). (**C**–**F**) Representative Western immunoblots (**C** and **E**) and quantitation (**D** and **F**) of H3K9ac (**C** and **D**) and H3K23ac (**E** and **F**) and pan H3 in the E18.5 cortex of *Kat6b^+/+^*, *Kat6b^+/–^*, and *Kat6b^–/–^* fetuses. (**G**–**J**) Representative Western immunoblots (**G** and **I**) and quantitation (**H** and **J**) of H3K9ac and pan H3 in the P14 (**G** and **H**) and P21 cortex (**I** and **J**) of *Kat6b^+/+^* and *Kat6b^+/–^* mice. (**K**) Flow cytometry gating strategy for adult mouse peripheral WBCs. (**L**) Median fluorescence intensity (MFI) of H3K9ac in WBCs of adult *Kat6b^+/+^* and *Kat6b^+/–^* mice. 0.5 μg histone protein loaded per lane; each lane represents an individual mouse (**C**, **E**, **G**, and **I**). *n =* 6 (**D** and **F**), 3 (**H** and **J**), and 3–5 (**L**) mice per genotype. Data are represented as mean ± SEM (**A**, **B**, **D**, **F**, and **H**) and were analyzed by 1-way ANOVA with Holm-Šidák correction (**A**, **B**, **D**, and **F**) or multiple *t* tests (**F**). Circles, individual mice. Related data in Supplemental Figures 5 and 6.

We generated *Kat6b* heterozygous mice carrying 1 *Kat6b* allele lacking exons 2–12 (*Kat6b^+/^*; Supplemental Figure 5, A–D). The deleted sequences encoded the N-terminal conserved (NEMM) domain, double plant homeodomain fingers (PHD), and the catalytic MYST histone acetyltransferase domain as well as alternative splice sites. *Kat6b^+/–^* heterozygous mice showed a 48% reduction in *Kat6b* mRNA in adult cortex tissue (*P =* 0.0006; Figure 2A) and a 50% reduction in gestational day 16.5 (E16.5) cultured cortical neurons (*P <* 10^–6^; Figure 2B). *Kat6b^–/–^* homozygous mutant cortical neurons showed no detectable *Kat6b* mRNA (*P <* 10^–6^; Figure 2B).

*Kat6b^+/–^* and *Kat6b^–/–^* mutant embryos were externally indistinguishable from controls at E12.5 (Supplemental Figure 5E). At E18.5, *Kat6b^–/–^* mutant fetuses displayed underdeveloped jaws (Supplemental Figure 5E) and shortened frontal bones. *Kat6b^–/–^* mutant mice were present at the expected Mendelian ratio in utero (Supplemental Figure 5F), but died before weaning. *Kat6b^+/–^* heterozygous mice were 19% underrepresented at weaning relative to WT controls (*P =* 0.00008; Supplemental Figure 5G). Compared with *Kat6b^+/+^* siblings, newborn *Kat6b^–/–^* mutants took a longer time to inflate their lungs, oxygenate their blood, and establish a regular breathing pattern (*P =* 8 × 10^–6^ to 0.0002; Supplemental Table 3), while *Kat6b^+/–^* achieved these milestones at the same time as *Kat6b^+/+^* siblings. As heterozygous mutations of the human *KAT6B* gene cause SBBYSS, heterozygous *Kat6b^+/–^* mice were considered the relevant genotype to model SBBYSS.

H3K9ac, H3K14, and H3K23ac were assessed in the cortex of E18.5 fetuses and 2- and 3-week-old mice and in the peripheral blood cells (WBCs) of adult mice, representing a clinically accessible cell type. E18.5 was a time point at which *Kat6b^–/–^* samples could be assessed in addition to *Kat6b^+/+^* and *Kat6b^+/–^* samples. We found a 49% and 18% reduction in H3K9ac in the E18.5 *Kat6b^–/–^* and *Kat6b^+/–^* cortex relative to controls, respectively (*P =* 0.03 and 3 × 10^–6^, respectively; Figure 2, C and D). H3K23ac was reduced by 12% in *Kat6b^–/–^* samples relative to controls (*P =* 0.01; Figure 2, E and F). No statistically significant effect of *Kat6b* status on H3K14ac was observed (Supplemental Figure 6, A and B). A reduction in H3K9ac in the *Kat6b^+/–^* cortex relative to controls was also observed at 2 weeks of age (*P =* 0.047; Figure 2, G and H), but not at 3 weeks of age (Figure 2, I and J). In adult mice, H3K9ac levels in WBCs were reduced in *Kat6b^+/–^* compared with control mice (*P =* 0.0001 to 0.04; Figure 2, K and L), but H3K23ac was unaffected (Supplemental Figure 6C). We found no statistically significant effect of *Kat6b* genotype on mitochondrial morphology or ATP output in E16.5 mouse cortical neurons (Supplemental Figure 6, D–H), consistent with HEK293T cells with early truncating SBBYSS mutations showing normal ATP output (Figure 1F).

### Kat6b^+/–^ mice display reduced vocalization and a delayed auditory startle response.

**Figure 3:**
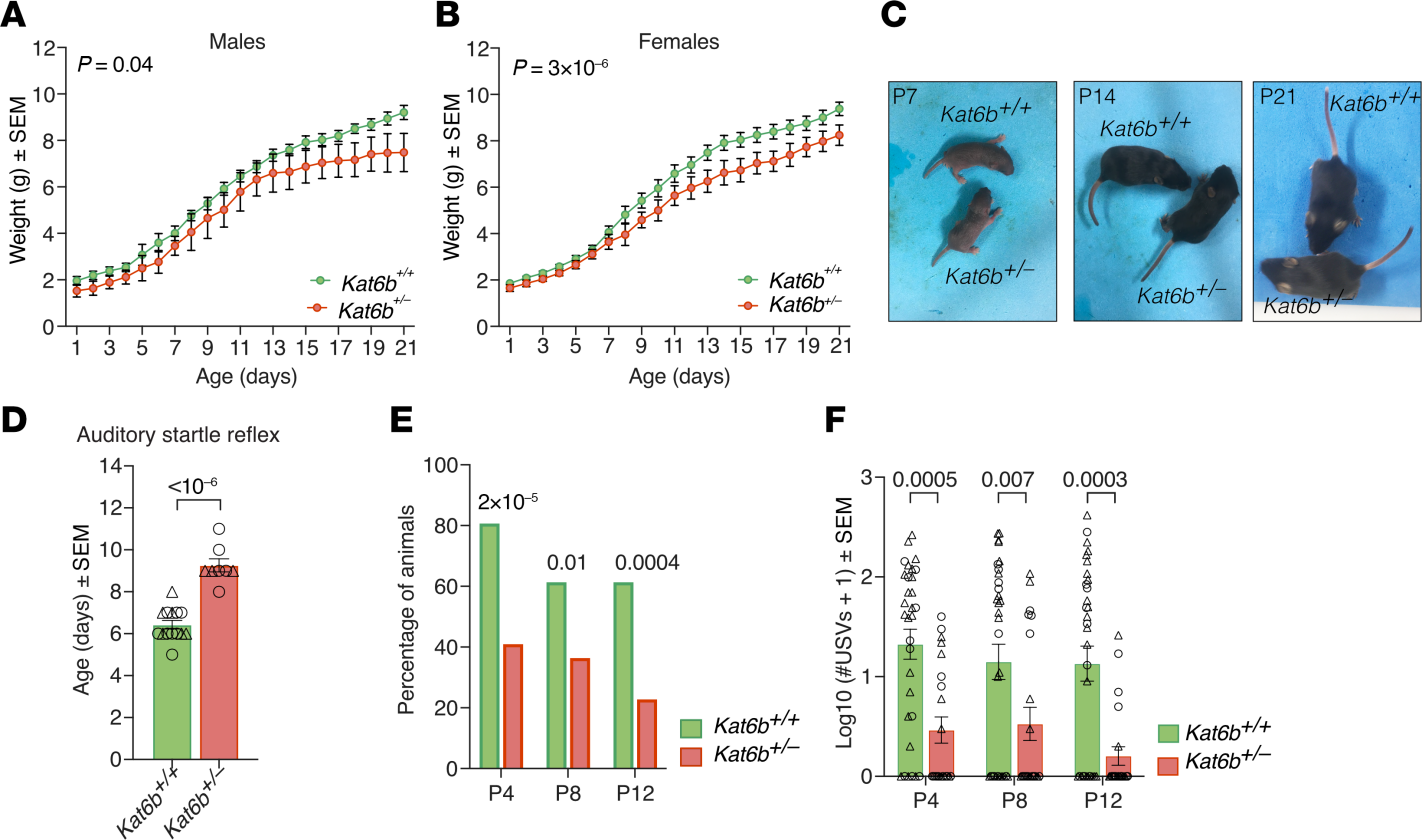
*Kat6b^+/–^* mouse pups display reduced vocalization and a delayed auditory startle response. (**A** and **B**) Body weights of male (**A**) and female (**B**) *Kat6b^+/+^* and *Kat6b^+/–^* mice from P1 to P21. (**C**) Representative images of *Kat6b^+/+^* and *Kat6b^+/–^* mice at P7, P14, and P21. (**D**) Age at which the auditory startle reflex was first observed in *Kat6b^+/+^* and *Kat6b^+/–^* mice. (**E**) Percentage of *Kat6b^+/+^* and *Kat6b^+/–^* mice that vocalized on P4, P8, and P12. (**F**) Number of USVs observed on P4, P8, and P12 in *Kat6b^+/+^* and *Kat6b^+/–^* mice. *n =* 15 *Kat6b^+/+^* (6 male/9 female [6M/9F]) and 8 *Kat6b^+/–^* mice (3M/5F) (**A**, **B**, and **D**); 31 *Kat6b^+/+^* (21M/10F) and 22 *Kat6b^+/–^* (9M/13F) (**E** and **F**). Data are represented as mean ± SEM (**A**, **B**, **D**, and **F**) or percentage (**E**) and were analyzed using 2-way ANOVA and Holm-Šidák correction (**A**, **B**, and **F**), Student’s *t* test (**D**), or χ^2^ test (**E**). Circles, triangles, individual female and male mice. Related data in Supplemental Figures 7 and 8.

At 3 weeks of age, *Kat6b^+/–^* mice weighed 12% (females) and 19% (males) less than sex-matched *Kat6b^+/+^* controls (*P =* 0.04 and 3 × 10^–6^; Figure 3, A–C). *Kat6b^+/–^* mice were otherwise normal, reaching physical and behavioral milestones at an age similar to that of *Kat6b^+/+^* siblings (Supplemental Figure 7, A–D). A notable exception was the auditory startle response, which *Kat6b^+/–^* mice displayed 3 days later than controls (*P <* 10^–6^; Figure 3D). This would be consistent with some individuals with KAT6B disorder displaying hearing impairments (3, 16), although it could also indicate delayed development of the motor startle, independent of hearing difficulties.

Assessment of maternal separation–induced ultrasonic vocalizations (USVs) revealed that a lower percentage of *Kat6b^+/–^* mice emitted vocalizations compared with *Kat6b^+/+^* mice (*P =* 2 × 10^–5^ to 0.01; Figure 3E). *Kat6b^+/–^* mice produced fewer vocalizations compared with *Kat6b^+/+^* mice (*P =* 0.0003 to 0.007; Figure 3F), even when nonvocal mice were excluded (Supplemental Figure 8A). The proportions of specific subtypes of vocalizations were largely similar between genotypes (Supplemental Figure 8, B and C). The observed reduction in vocalizing in *Kat6b^+/–^* mice suggests a vocalization impairment, consistent with the speech impairments of individuals with KAT6B disorder (3, 16).

### Prerequisites for behavioral studies on Kat6b^+/–^ adult mice.

Unlike in *Kat6b* homozygous mutant mice, which displayed a range of brain defects (6), no gross histological or morphometric differences were observed between the brains of *Kat6b^+/–^* and *Kat6b^+/+^* mice (Supplemental Figure 9). *Kat6b* heterozygosity did not affect motor coordination, muscle strength or vision, general activity, the sleep/wake cycle, or motor parameters in the home cage (Supplemental Figure 10, A–T), suggesting that *Kat6b^+/–^* mice had the basic capability of performing behavioral tests. No effects of sex were observed in any of the behavior tests conducted. Nevertheless, we have indicated the sexes as circles (females) and triangles (males) in bar graphs.

### Kat6b^+/–^ mice spend more time in the open, suggesting decreased anxiety.

**Figure 4:**
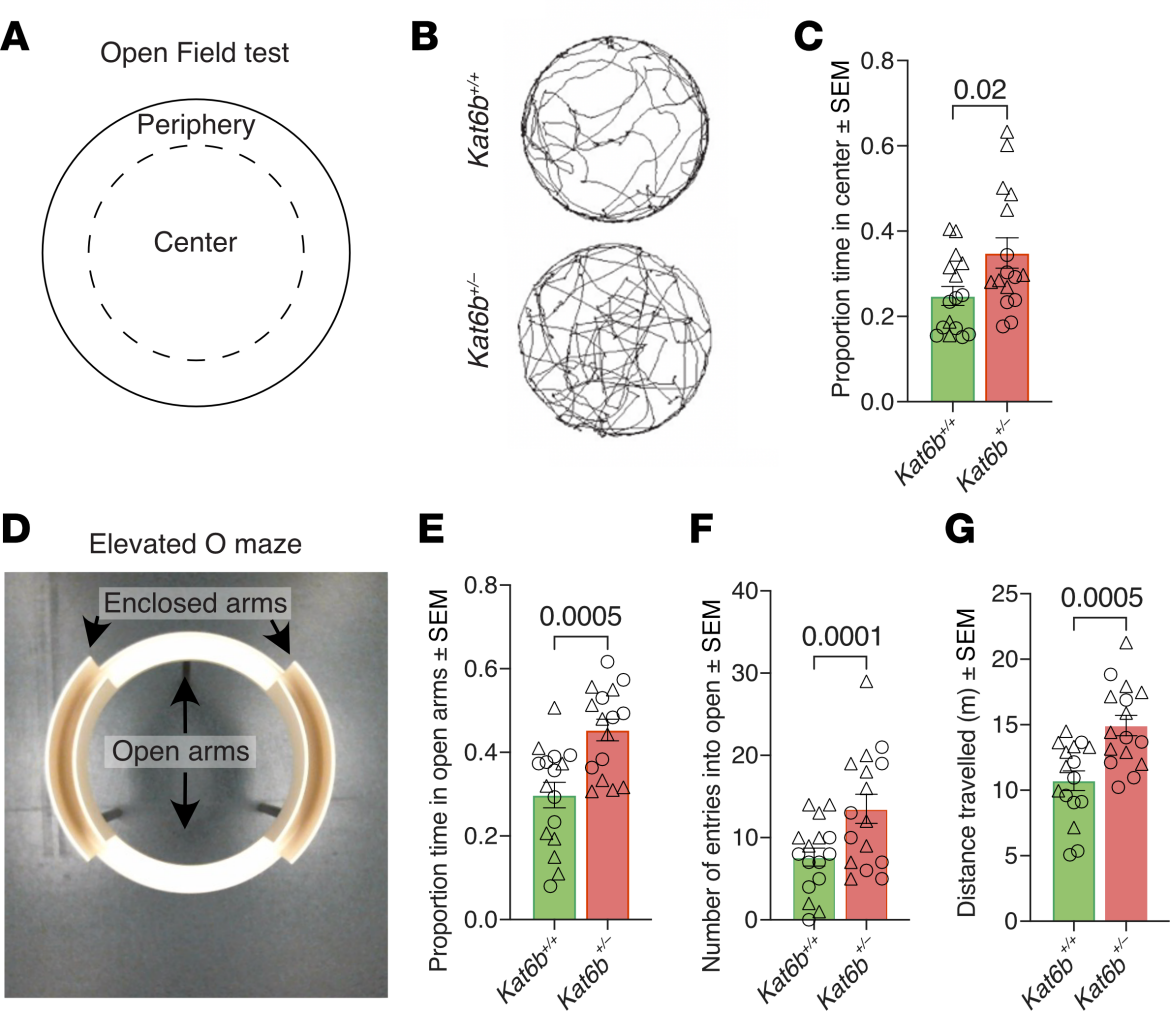
Adult *Kat6b^+/–^* mice spend a greater proportion of time in the open. (**A**–**C**) Diagram of the open field (**A**), representative traces of *Kat6b^+/+^* and *Kat6b^+/–^* mouse movements in 5 minutes of the 20-minute testing time (**B**), and proportion of time spent in the center (**C**) of the open field. (**D**–**G**) Image of the elevated O maze (**D**), proportion of time spent in the open arms (**E**), number of entries into the open arms (**F**), and total distance traveled (**G**) in the elevated O maze. *n =* 16 *Kat6b^+/+^* (8M/8F) and 16 *Kat6b^+/–^* (9M/7F) 12-week-old mice per genotype. Data are represented as mean ± SEM and were analyzed using Student’s *t* test (**C**, **E**, **F**, and **G**). Circles, triangles, individual female and male mice. Related data in Supplemental Figures 9, 10, and 11.

In the large open field, *Kat6b^+/–^* mice spent a 40% greater proportion of testing time in the center compared with WT controls (*P =* 0.02; Figure 4, A–C). *Kat6b^+/–^* mice also spent more absolute time in the center and traveled a greater distance in the center (*P =* 0.03 and 0.02; Supplemental Figure 11, A–F). In the elevated O maze, *Kat6b^+/–^* mice spent a 50% greater proportion of time in the open sections than *Kat6b^+/+^* mice (*P =* 0.0005; Figure 4, D and E), showed a 74% increase in the number of entries into the open sections (*P =* 0.0001; Figure 4F), and traveled a greater total distance (*P =* 0.0005; Figure 4G).

### Kat6b^+/–^ mice show impaired learning and memory.

Learning difficulties and a global developmental delay are the most commonly described traits across individuals with KAT6B disorder (3, 17). To assess learning and memory, the novel object–recognition test, Y maze, Barnes maze, and foot-shock fear-conditioning tests were performed.

**Figure 5:**
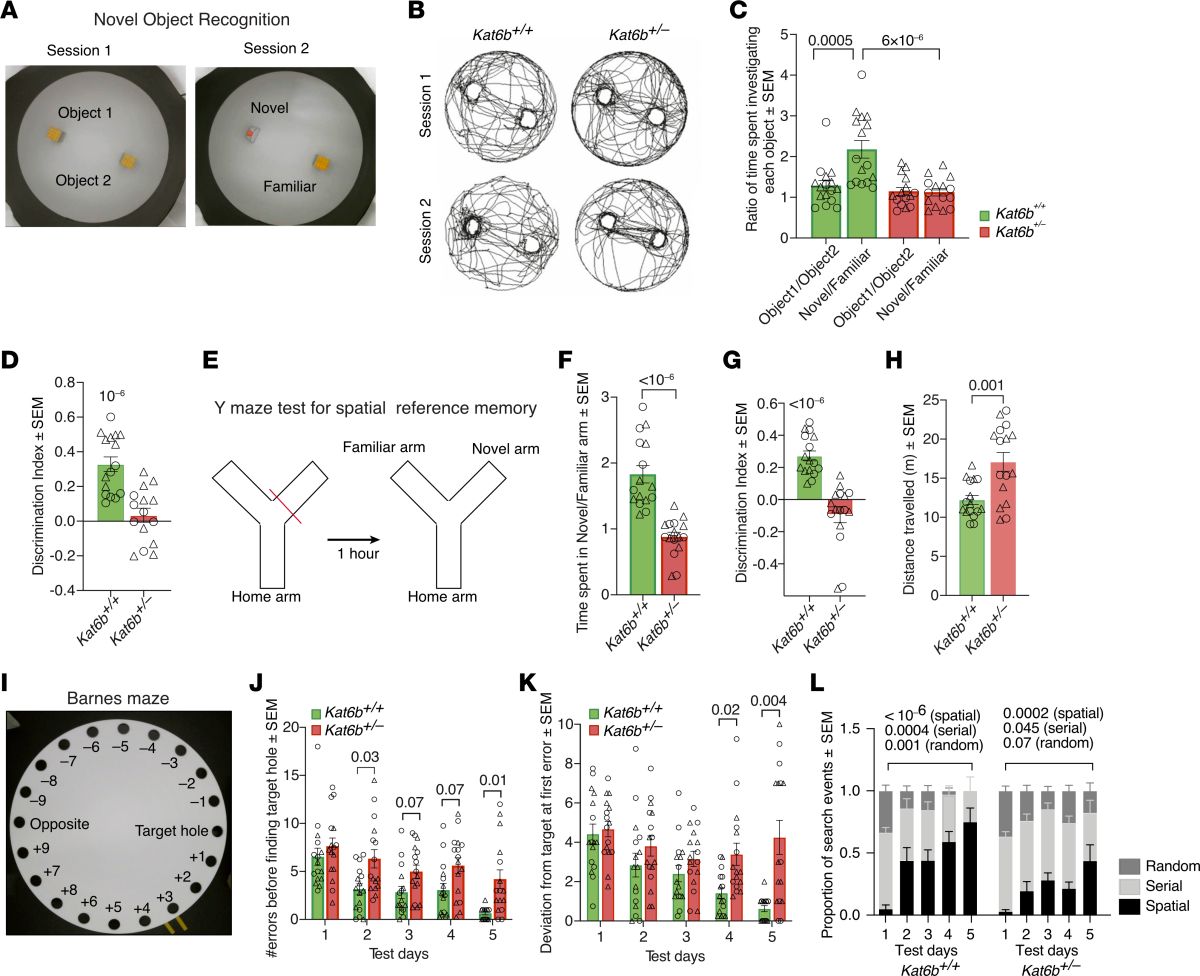
*Kat6b^+/–^* mice display learning and memory deficits. (**A**–**D**) Images (**A**) and representative traces (**B**) of the novel object–recognition session 1 (2 identical blocks) and session 2 (1 familiar and 1 novel object), ratio of time spent around each object in session 1 and 2 (**C**), and discrimination index for novel over familiar object (**D**). (**E**–**H**) Depiction of Y maze spatial recognition–memory test (**E**), ratio of time spent in novel over familiar arm (**F**), discrimination index for the novel over the familiar arm (**G**), and total distance traveled (**H**). (**I**–**L**) Image of Barnes maze with hole positions labeled (**I**), number of errors made before finding target hole across 4 trials per day of training (days 1–4) and during 24-hour recall session (day 5) (**J**), deviation from target hole at first error (**K**), and proportion of random, serial, and spatial search strategies used to find target hole (**L**) in the Barnes maze. *n =* 16 *Kat6b^+/+^* (8M/8F) and 15–16 *Kat6b^+/–^* (9M/6-7F) mice. Data are represented as mean ± SEM and were analyzed by 2-way ANOVA with Holm-Šidák correction (**C**, **J**, **K**, and **L**), 1-sample *t* test comparing with 0 (**D** and **G**), and Student’s *t* test (**F** and **H**). Circles, triangles, individual female and male mice. Related data in Supplemental Figure 11.

In the novel object–recognition test, *Kat6b^+/–^* mice showed no preference, whereas *Kat6b^+/+^* mice showed a 1.7-fold preference for the novel over the familiar object (*P =* 0.0005; discrimination index *P =* 10^–6^; Figure 5, A–D), indicating impaired recognition memory in *Kat6b^+/–^* mice. No difference was observed between *Kat6b^+/+^* and *Kat6b^+/–^* mice in the spontaneous alternations between arms of the Y maze or total distance traveled (Supplemental Figure 11, G–I), indicating a functioning working memory. In the Y maze for spatial reference memory, the ratio of time spent in the previously closed, novel arm, relative to the previously explored familiar arm, was 52% reduced in *Kat6b^+/–^* mice compared with *Kat6b^+/+^* controls (*P <* 10^–6^; Figure 5, E and F). *Kat6b^+/–^* mice did not show a preference, whereas *Kat6b^+/+^* mice showed a preference for the novel arm (*P <* 10^–6^; Figure 5G), suggesting impaired spatial reference memory in *Kat6b^+/–^* mice. *Kat6b^+/–^* mice traveled a greater total distance in this Y maze test (*P =* 0.001; Figure 5H). Assessment of associative memory using the foot-shock fear-conditioning test revealed no difference between genotypes (Supplemental Figure 11, J and K), indicating that *Kat6b^+/–^* mice were capable of associative learning with an adverse stimulus.

In the Barnes maze, during the 4-day training phase (Figure 5, I–K), *Kat6b^+/–^* mice showed an approximately 2-fold greater number of errors before finding the target hole on day 2 (*P =* 0.03; Figure 5J) and 2.4-fold greater deviation from the target on day 4 compared with *Kat6b^+/+^* mice (*P =* 0.02; Figure 5K). At the 24-hour recall on day 5, *Kat6b^+/–^* mice showed a 6.7-fold greater number of errors before finding the target hole (*P =* 0.01; Figure 5J) and 6.8-fold greater deviation from the target at first error compared with controls (*P =* 0.004; Figure 5K). Both *Kat6b^+/+^* and *Kat6b^+/–^* mice displayed search strategy learning over the 5 days (Figure 5L). *Kat6b^+/+^* mice increased in the proportion of direct spatial accesses to the target hole 15-fold (*P <* 10^–6^; Figure 5L), replacing random searches entirely (*P =* 0.001) and 59% of the serial searches (*P =* 0.0004). Although *Kat6b^+/–^* mice improved the proportion of direct spatial accesses to the target hole (*P =* 0.0002; Figure 5L), they did not reach the same level as the controls. *Kat6b^+/–^* mice increased the spatial-search strategy at the expense of serial searches (*P =* 0.045), but did not show a statistically significant reduction in random searches.

### Kat6b^+/–^ mice display impaired social interaction and social recognition.

**Figure 6:**
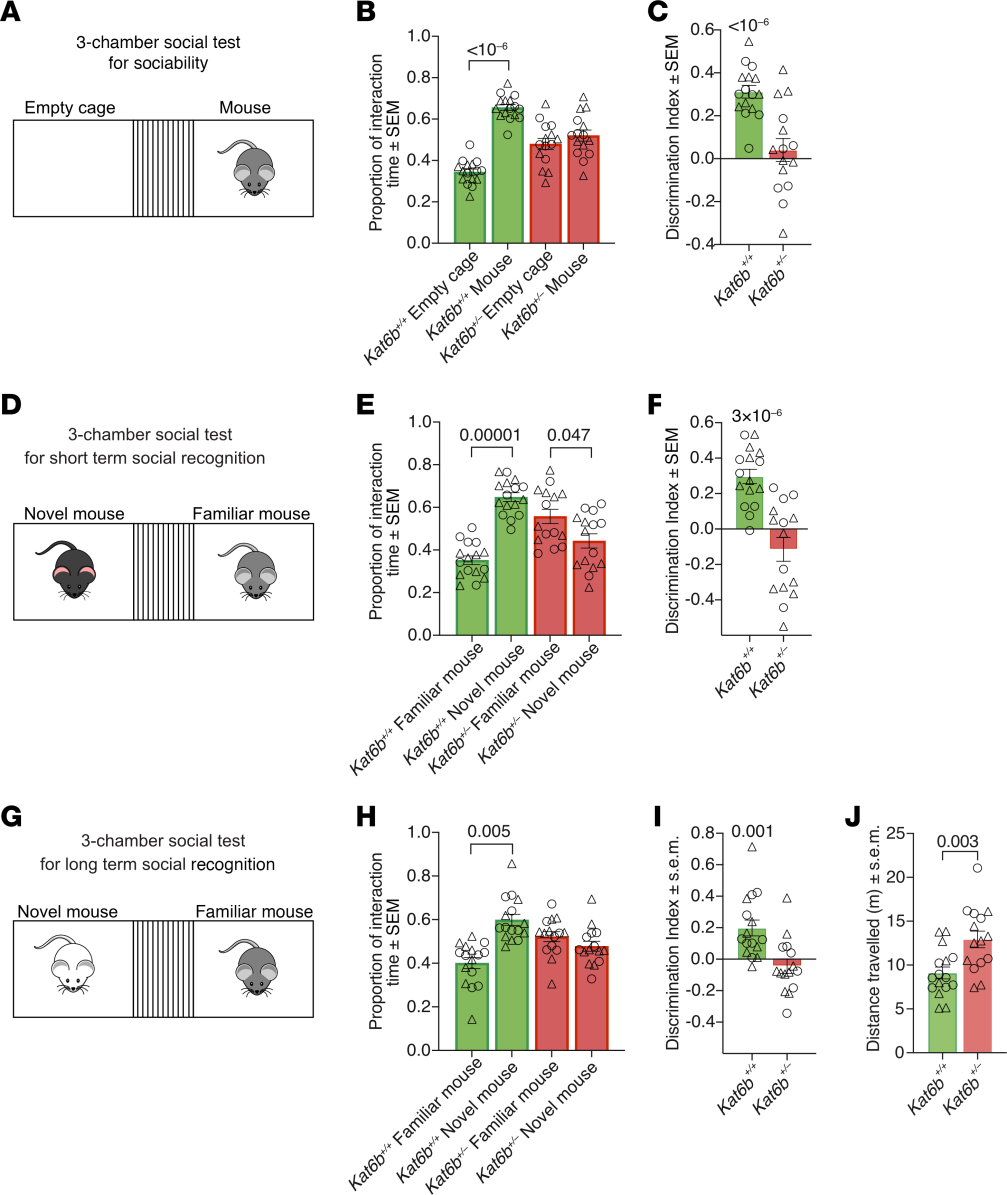
*Kat6b^+/–^* mice show reduced sociability and social recognition. (**A**–**J**) Three-chamber social test. Session 1: mouse versus empty cage (**A**–**C**), session 2: short-term social recognition (1 hour) novel versus familiar mouse (**D**–**F**), session 3: long-term social recognition (24 hours) novel versus familiar mouse (**G**–**J**). Proportion of time spent around the empty cage and cage with mouse (**B**). Discrimination index for the mouse over the empty cage (**C**). Proportion of time spent around the novel mouse and the familiar mouse (**E** and **H**). Discrimination index for the novel over the familiar mouse (**F** and **I**). Total distance traveled in session 3 of the 3-chamber social test (**J**). *n =* 16 *Kat6b^+/+^* (8M/8F) and 15 *Kat6b^+/–^* (8-9M/6-7F) mice. Data are represented as mean ± SEM and were analyzed using 2-way ANOVA with Holm-Šidák correction (**B**, **E**, and **H**), 1-sample *t* test comparing with 0 (**C**, **F**, and **I**), and Student’s *t* test (**J**). Circles, triangles, individual female and male mice. Related data in Supplemental Figure 11.

In session 1 of the 3-chamber social test, *Kat6b^+/+^* mice spent 1.9-fold more time with the mouse compared with the empty cage (*P <* 10^–6^; Figure 6, A–C), whereas *Kat6b^+/–^* mice showed no preference for the mouse, indicating impaired sociability (Figure 6, B and C). In session 2, 1 hour later, *Kat6b^+/+^* mice spent 1.9-fold more time with the novel mouse compared with the familiar mouse (*P =* 10^–5^ and 3 × 10^–6^; Figure 6, D–F). *Kat6b^+/–^* mice failed to show a preference (Figure 6, E and F), suggesting a lack of social recognition. In the final session, 24 hours later, *Kat6b^+/+^* mice spent 1.4-fold more time with the novel mouse compared with the familiar mouse (*P =* 0.005 and 0.001; Figure 6, G–I). *Kat6b^+/–^* mice showed no preference (Figure 6, H and I), suggesting a lack of long-term social recognition. Mice of both genotypes traveled comparable distances in sessions 1 and 2 (Supplemental Figure 11, L and M); however, *Kat6b^+/–^* traveled a greater distance in session 3 (*P =* 0.003; Figure 6J). While *Kat6b^+/–^* mice showed a slight preference for the familiar mouse in session 2 (*P =* 0.047; Figure 6E), they approached the novel mouse, indicating no aversion to the novel mouse.

### Assessing experimental treatments.

H3K9ac levels were reduced in WBCs of *Kat6b^+/–^* mice (Figure 2L) and in the developing cortex of *Kat6b^+/–^* and *Kat6b^–/–^* mice (Figure 2, D and H). The enzymatic reaction opposing histone acetylation is histone deacetylation, catalyzed by HDACs. Inhibition of HDACs could alleviate an acetylation deficiency and could modulate the behavioral deficits of *Kat6b* heterozygous mice. Alternatively, it might be possible to boost the activity of the KAT6B protein produced by the single healthy copy of the *Kat6b* gene by providing more substrate in the form of an acetyl-group donor.

### Valproic acid treatment of Kat6b^+/–^ and control mice.

**Figure 7:**
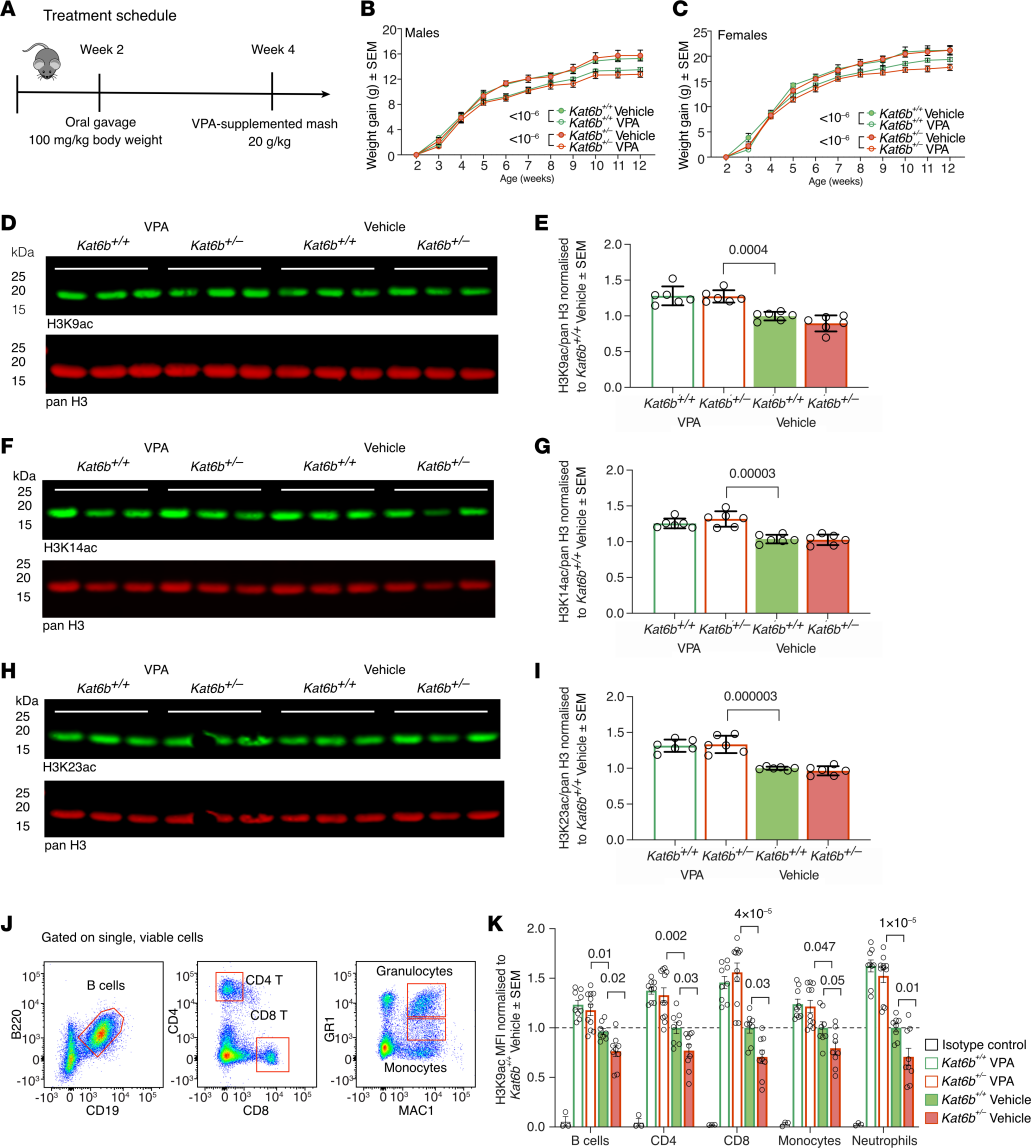
VPA treatment increases H3K9ac in *Kat6b^+/–^* and *Kat6b^+/+^* mice. (**A**) Treatment schedule for VPA or vehicle. (**B** and **C**) Weight gain from 2 to 12 weeks of age in VPA- and vehicle-treated male (**B**) and female (**C**) *Kat6b^+/+^* and *Kat6b^+/–^* mice. (**D**–**I**) Representative Western immunoblots and quantitation of H3K9ac (**D** and **E**), H3K14ac (**F** and **G**), H3K23ac (**H** and **I**), and pan H3 (**D**, **F**, and **H**) in the cortex of adult *Kat6b^+/+^* and *Kat6b^+/–^* mice treated with VPA or vehicle since 2 weeks of age. Each lane represents an individual mouse. 0.5 μg (H3K9ac, H3K23ac) or 2 μg (H3K14ac) of protein loaded per lane. (**J**) Flow cytometry gating strategy for WBCs after size selection and gating on viable cells. H3K9ac was quantitated within each cell type. (**K**) H3K9ac MFI in WBCs as assessed by flow cytometry, normalized to the *Kat6b^+/+^* vehicle group. Isotype negative control for 3 independent experiments is shown. *n =* 6–9 (6–8M/7–9F; **B** and **C**), 6 (**D**–**I**), and 8–11 (**K**) mice per genotype and treatment group. Data are represented as mean ± SEM and were analyzed using a 3-way (**B** and **C**) or 2-way (**E**, **G**, **I**, **K**) ANOVA with Holm-Šidák correction. Circles, triangles, individual female and male mice. Related data in Supplemental Figures 12 and 13.

Valproic acid (VPA) is a broad-spectrum HDAC inhibitor, currently in clinical use for the treatment of epilepsy, bipolar disorder, depression, and schizophrenia (18). To model intervention in young children, we treated *Kat6b* heterozygous and WT mice from 2 weeks of age (Figure 7A), equivalent to 2 to 3 years of age in humans (19). Behavioral tests for which statistically significant differences were observed between untreated *Kat6b^+/–^* and WT mice were performed. In addition, we determined whether treatment affected motor activity or vision. VPA-treated *Kat6b^+/+^* and *Kat6b^+/–^* mice gained 9% and 16% less weight, respectively, compared with genotype- and sex-matched vehicle controls (*P <* 10^–6^; Figure 7, B and C), a VPA effect reported previously (20). Otherwise, VPA-treated mice showed no overt adverse effects.

H3K9, H3K14, and H3K23 acetylation levels in the adult cortex were increased 1.2- to 1.4-fold in mice of both genotypes treated with VPA from 2 to 12–14 weeks of age compared with vehicle-treated *Kat6b^+/+^* controls (*P =* 0.0004 to 3 × 10^–6^; Figure 7, D–I). Similarly, a shorter VPA treatment for 5 days from 2 weeks of age onward increased H3K9ac levels in both genotypes (*P =* 0.03; Supplemental Figure 12, A and B). Like untreated mice (Figure 2L), vehicle-treated *Kat6b^+/–^* mice showed reduced H3K9ac levels relative to vehicle-treated *Kat6b^+/+^* controls in WBCs (*P =* 0.01 to 0.05; Figure 7, J and K). VPA-treated mice showed a 1.2- to 2.2-fold increase in H3K9ac levels across all cell types, relative to vehicle-treated mice (*P =* 10^–5^ to 0.047; Figure 7K). Therefore, WBCs, which are clinically accessible cells, may be useful for monitoring the effects of treatment on histone acetylation.

VPA has also been shown to drive hematopoietic stem and progenitor cell expansion (21). Indeed, VPA-treated *Kat6b^+/+^* and *Kat6b^+/–^* mice showed a 1.4-fold greater percentage of hematopoietic stem cells (HSCs) in the bone marrow than their respective vehicle controls (*P =* 0.03 and 0.046, respectively; Supplemental Figure 12, C and D). VPA-treated *Kat6b^+/–^* mice also had elevated WBCs in the bone marrow compared with vehicle-treated *Kat6b^+/–^* mice (*P =* 0.01; Supplemental Figure 12E). Akin to the cortex and WBCs of VPA-treated mice, VPA treatment elevated H3K9, K14, and K23 acetylation levels 2- to 3-fold in HEK293T cells with SBBYSS mutations compared with vehicle-treated control cells (*P <* 10^–6^; Supplemental Figure 13, A–F).

Behavioral testing was performed on VPA- and vehicle-treated mice from 8 weeks of age. In the rotor-rod test, no difference was observed in the latency to fall between genotypes within treatment groups (Supplemental Figure 14A). However, VPA-treated mice of both genotypes showed a shorter latency to fall from 25 rpm onwards than genotype-matched vehicle controls (Supplemental Figure 14A), indicating that VPA treatment caused impaired motor coordination or strength. This effect has been reported previously in mice treated postnatally (22), while movement disorders have been reported in a subset of individuals treated with valproate (23). Mice of both genotypes and treatment groups spent more time on the shallow side of the visual cliff test, indicating that VPA treatment did not affect vision (Supplemental Figure 14B).

**Figure 8:**
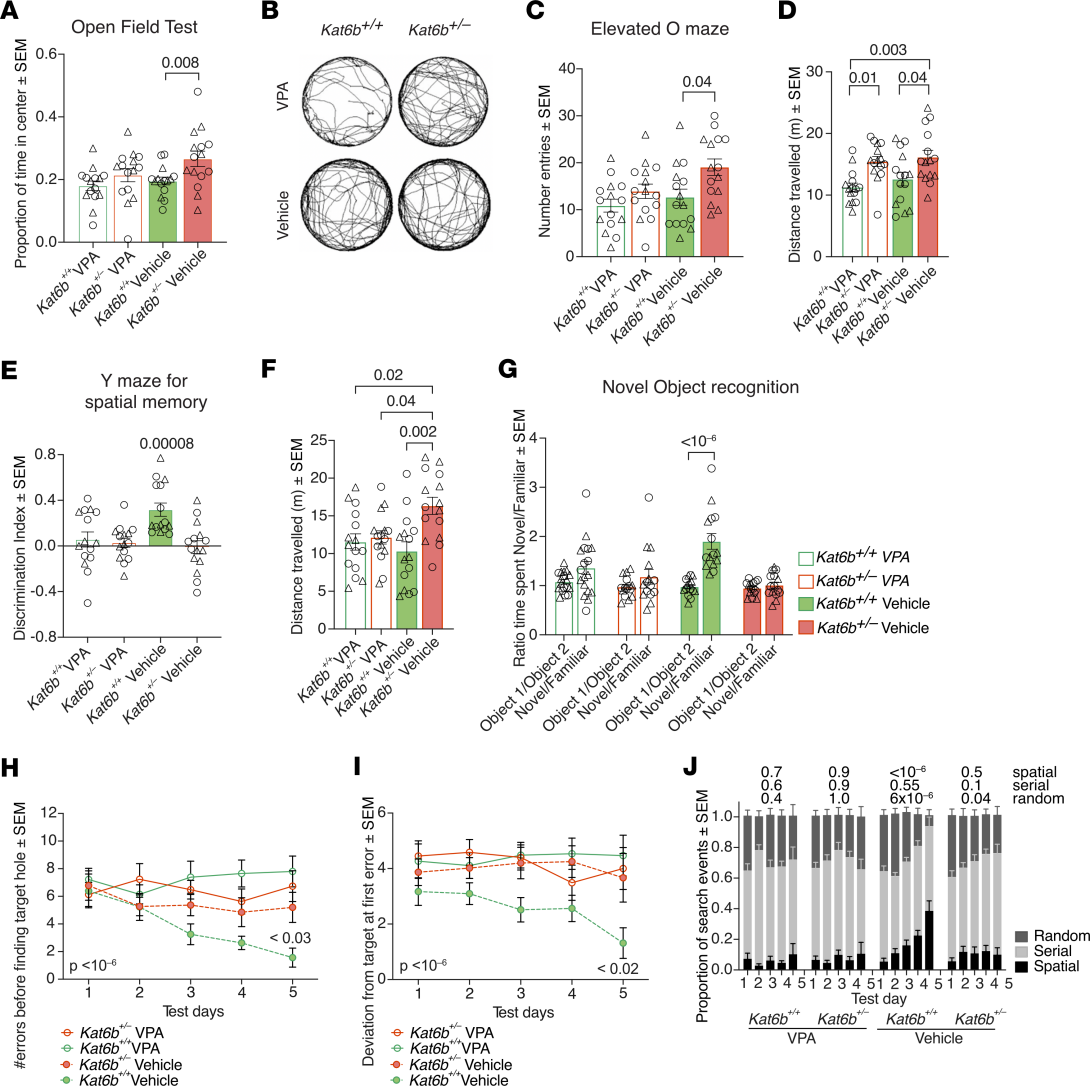
VPA treatment does not improve learning and memory in *Kat6b^+/–^* mice. (**A** and **B**) Proportion of time spent in the center of the open-field arena (**A**) and representative traces of movement in 5 minutes of the 20-minute testing time (**B**). (**C** and **D**) Total number of entries into the open arms (**C**) and distance traveled (**D**) in the elevated O maze. (**E** and **F**) Discrimination index for the novel over the familiar arm (**E**) and distance traveled in the Y maze for spatial memory (**F**). (**G**) Ratio of time spent around object 1 and object 2 or the novel and familiar object in the novel object–recognition test. (**H**–**J**) Number of errors before finding the target (**H**), deviation from the target at first error (**I**), and search strategy used (**J**) in the Barnes maze. *n =* 15–18 *Kat6b^+/+^* (6–11M/6–9F) and 15 *Kat6b^+/–^* (5–8M/7–10F) mice per treatment group. Data are represented as mean ± SEM and were analyzed using 2-way (**A**, **C**, **D**, and **F**) or 3-way (**G**–**J**) ANOVA with Holm-Šidák correction or 1-sample *t* test comparing with 0 (**E**). Circles, triangles, individual female and male mice. Related data in Supplemental Figure 14.

In the open-field test, vehicle-treated *Kat6b^+/–^* mice spent more time in the center of the arena compared with vehicle-treated *Kat6b^+/+^* controls (*P =* 0.008; Figure 8, A and B), as observed in the baseline analysis (Figure 4C). In comparison, *Kat6b^+/–^* VPA-treated mice spent a proportion of time in the center of the arena similar to that of VPA- and vehicle-treated *Kat6b^+/+^* mice (Figure 8, A and B), suggesting that VPA treatment restored normal anxiety levels to *Kat6b^+/–^* mice in this test. No difference was observed for any motor parameter in the open-field test between vehicle-treated genotypes (Supplemental Figure 14, C–H), consistent with the baseline assessment (Supplemental Figure 10, F–K). Curiously, VPA treatment affected some movement parameters in *Kat6b^+/+^* mice that were not affected in VPA-treated *Kat6b^+/–^* mice (Supplemental Figure 14, G and H).

In the elevated O maze, vehicle-treated *Kat6b^+/–^* mice spent more time in the open areas compared with vehicle-treated *Kat6b^+/+^* mice (*P =* 0.003; Supplemental Figure 14I), entered the open arms more frequently (*P =* 0.04; Figure 8C), and traveled a greater total distance (*P =* 0.04; Figure 8D), as in the baseline assessment (Figure 4, E–G). The proportion of time spent by VPA-treated *Kat6b^+/–^* mice in the open arms was not statistically significantly different from that of VPA-treated *Kat6b^+/+^* mice, but was greater than that of vehicle-treated *Kat6b^+/+^* mice (*P =* 0.01; Supplemental Figure 14I). Interestingly, VPA-treated *Kat6b^+/–^* mice did not enter the open arms more often than VPA- or vehicle-treated *Kat6b^+/+^* mice (Figure 8C) and VPA-treated *Kat6b^+/–^* mice did not travel a statistically significantly greater distance than *Kat6b^+/+^* vehicle-treated controls (Figure 8D). This suggests that VPA treatment led to a partial restoration of normal anxiety behavior in *Kat6b^+/–^* mice in this test.

In the Y maze for spatial reference memory, vehicle-treated *Kat6b^+/–^* mice failed to show a preference for the novel arm (Figure 8E), as in the baseline assessment (Figure 5, F and G). Neither *Kat6b^+/+^* nor *Kat6b^+/–^* VPA-treated mice showed a preference for the novel arm (Figure 8E). As seen in the baseline (Figure 5H), vehicle-treated *Kat6b^+/–^* mice traveled a greater distance than *Kat6b^+/+^* vehicle controls (*P =* 0.002, Figure 8F), while distance was comparable between both VPA-treated genotypes and vehicle-treated *Kat6b^+/+^* mice.

In the novel object–recognition test, *Kat6b^+/–^* vehicle-treated mice failed to spend more time with the novel object (Figure 8G), as seen in the baseline cohort (Figure 5, C and D). Vehicle-treated *Kat6b^+/+^* mice spent 2-fold more time with the novel over the familiar object (*P <* 10^–6^, Figure 8G). Neither VPA-treated genotype spent more time with the novel object (Figure 8G).

In the Barnes maze, vehicle-treated *Kat6b^+/–^* mice, VPA-treated *Kat6b^+/–^* mice, and VPA-treated *Kat6b^+/+^* mice showed impaired spatial learning (Figure 8, H and I). While vehicle-treated *Kat6b^+/+^* control mice learned to reduce the proportion of random searches and increased the proportion of spatial access to the target hole over the training and recall sessions, neither vehicle- nor VPA-treated *Kat6b^+/–^* mice showed an increase in spatial access and even VPA-treated *Kat6b^+/+^* mice failed to display learning in this test (Figure 8J), indicating that VPA not only failed to improve the spatial learning and memory impairment in *Kat6b^+/–^* mice, but also compromised the spatial learning and memory of WT mice.

**Figure 9:**
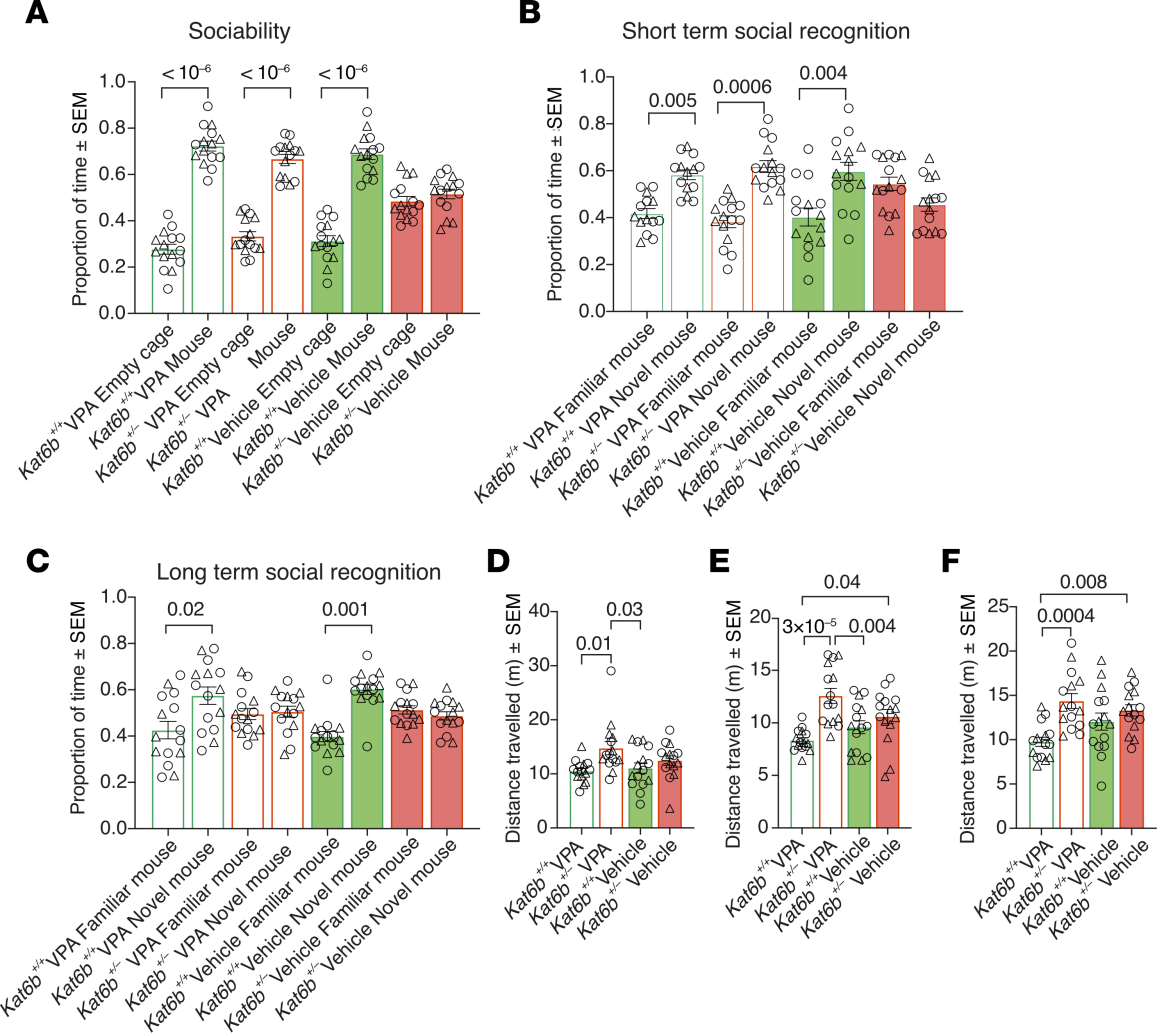
VPA treatment improves social behavior in *Kat6b^+/–^* mice. (**A**–**F**) Three-chamber social test. Proportion of time spent around the empty cage and the mouse (**A**). Proportion of time spent around the familiar and the novel mouse (1-hour recall) (**B**). Proportion of time spent around the familiar and the novel mouse (24-hour recall) (**C**). Total distance traveled in session 1 (**D**), session 2 (**E**), and session 3 (**F**). *n =* 15 *Kat6b^+/+^* (6–7M/8–9F) and 15 *Kat6b^+/–^* (7–8M/7–8F) mice per treatment group. Data are represented as mean ± SEM and were analyzed using 3-way (**A**, **B**, and **C**) or 2-way (**D**, **E**, and **F**) ANOVA with Holm-Šidák correction. Circles, triangles, individual female and male mice. Related data in Supplemental Figure 14.

In the 3-chamber social test, vehicle-treated *Kat6b^+/–^* mice showed no preference for the mouse over an empty cage (Figure 9A and Supplemental Figure 14J), as in the baseline assessment (Figure 6, B and C). Remarkably, VPA-treated *Kat6b^+/–^* mice showed a 2-fold preference for the mouse over the empty cage (*P <* 10^–6^; Figure 9A and Supplemental Figure 14J), like vehicle and VPA-treated *Kat6b^+/+^* mice. Furthermore, *Kat6b^+/–^* VPA-treated mice showed a 1.6-fold preference for the novel mouse over the familiar mouse in the short-term social-recognition test (*P =* 0.0006; Figure 9B and Supplemental Figure 14K), similarly to vehicle- and VPA-treated *Kat6b^+/+^* mice. In session 3, 24 hours later, vehicle and VPA-treated *Kat6b^+/+^* mice showed a preference for the novel mouse over the familiar mouse (*P =* 0.02 and 0.001; Figure 9C and Supplemental Figure 14L), but neither VPA nor vehicle-treated *Kat6b^+/–^* mice showed a preference (Figure 9C and Supplemental Figure 14L). Across sessions, VPA-treated *Kat6b^+/–^* mice traveled a greater distance than VPA-treated *Kat6b^+/+^* mice (Figure 9, D–F). Unlike in the baseline cohort (Figure 6J), vehicle-treated *Kat6b^+/–^* mice traveled distances similar to those of controls (Figure 9, D–F).

### Acetyl-carnitine treatment of Kat6b^+/–^ and control mice.

Acetyl-carnitine (ALCAR) is a naturally occurring amine that can act as an acetyl donor for histone acetyltransferases, promoting histone acetylation by raising the levels of the acetyl–coenzyme A substrate (24). ALCAR has been the subject of widespread interest due its potential to ameliorate neurodegenerative conditions (25).

**Figure 10:**
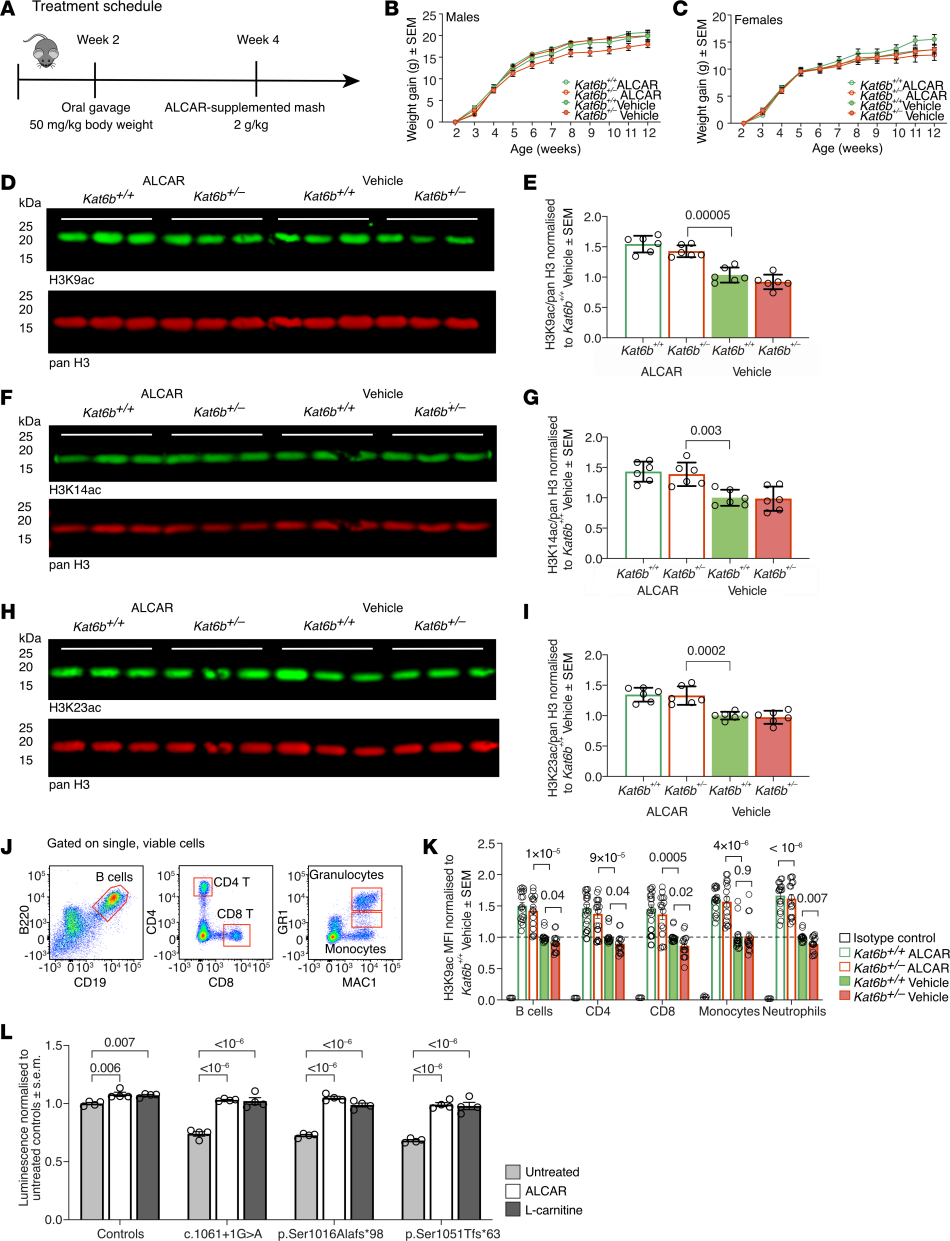
ALCAR treatment increases H3K9ac in *Kat6b^+/–^* and *Kat6b^+/+^* mice. (**A**) Treatment schedule for ALCAR or vehicle. (**B** and **C**) Weight gain from 2 to 12 weeks of age in ALCAR- and vehicle-treated male (**B**) and female (**C**) *Kat6b^+/+^* and *Kat6b^+/–^* mice. (**D**–**I**) Representative Western immunoblots (**D**, **F**, and **H**) and quantitation (**E**, **G**, and **I**) of H3K9ac (**D** and **E**), H3K14ac (**F** and **G**), H3K23ac (**H** and **I**), and pan H3 in the cortex of adult *Kat6b^+/+^* and *Kat6b^+/–^* mice treated with ALCAR or vehicle since 2 weeks of age. Each lane represents an individual mouse. 0.5 μg (H3K9ac, H3K23ac) or 2 μg (H3K14ac) of protein loaded per lane. (**J**) Flow cytometry gating strategy for WBCs after size selection and gating on viable cells. (**K**) H3K9ac MFI in WBCs. (**L**) ATP levels assessed by Mitochondrial ToxGlo assay. Luminescence in control and SBBYSS HEK239T cells treated with vehicle, 1 mM ALCAR, or 1 mM l-carnitine. *n =* 5–11 (6–11M/5–9F; **B** and **C**), 6 (**E**, **G**, and **I**), and 15–18 (**K**) mice or 4 clonal cell lines (**L**) per genotype and treatment. Data analyzed using 3-way (**B** and **C**) or 2-way (**E**, **G**, and **I**–**L**) ANOVA with Holm-Šidák correction. Circles, individual mice (**E**, **G**, **I**, and **K**) or clonal cell lines (**L**). Related data in Supplemental Figures 15 and 16.

ALCAR-treated mice showed no adverse effects or difference in weight gain compared with vehicle-treated mice (Figure 10, A–C). In the cortex, H3K9, H3K14, and H3K23 acetylation levels were increased by 1.4-fold in mice of both genotypes treated with ALCAR from 2 weeks of age for 10 to 12 weeks compared with vehicle-treated controls (*P =* 0.003 to 5 × 10^–5^; Figure 10, D–I) and increased 1.3- to 1.8-fold in ALCAR-treated SBBYSS HEK293T cells compared with vehicle-treated control cells (Supplemental Figure 15, A–F). Similarly, a shorter ALCAR treatment for 5 days from 2 weeks of age onward increased H3K9ac in the mouse cortex in both genotypes (*P =* 0.03; Supplemental Figure 16, A and B). ALCAR treatment resulted in a 1.4- to 1.8-fold increase in H3K9ac levels in WBCs in mice of both genotypes compared with vehicle-treated genotypes (*P =* 0.0005 to <10^–6^; Figure 10, J and K), but did not affect the proportion of hemopoietic cells (Supplemental Figure 16, C and D). As ALCAR as well as its unacetylated form, l-carnitine, have important roles in mitochondrial function (24, 25), we assessed whether they could restore the defective ATP output in SBBYSS HEK293T cell lines (Figure 1F). Treatment with 1 mM ALCAR or l-carnitine elevated ATP output in cells with the 3 mutations that previously showed a reduction in ATP output (*P <* 10^–6^ to 0.007; Figure 10L).

**Figure 11:**
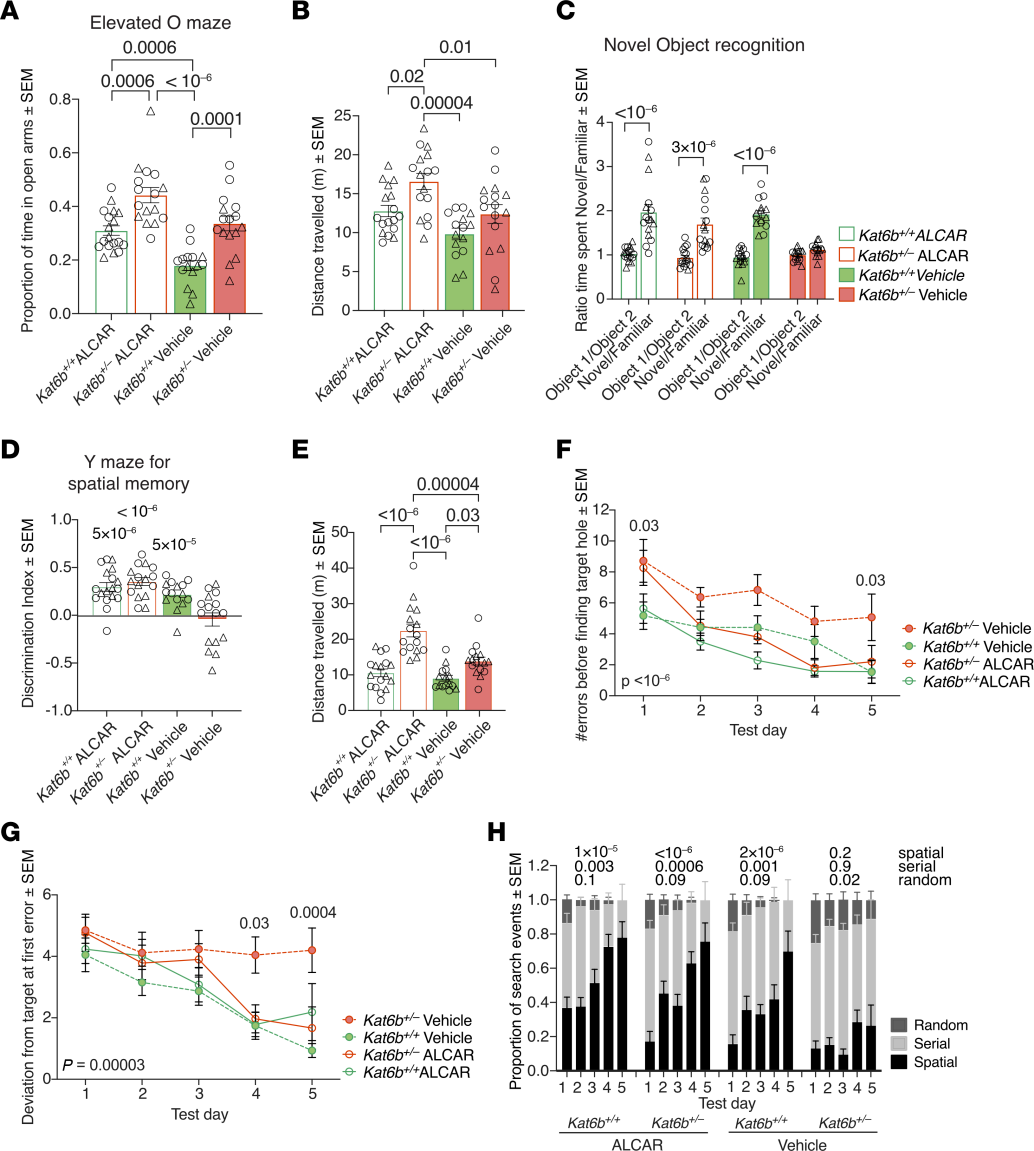
ALCAR treatment restores spatial learning and memory in *Kat6b^+/–^* mice. (**A** and **B**) Proportion of time spent in the open arms (**A**) and distance traveled (**B**) in the elevated O maze. (**C**) Ratio of time spent investigating object 1 and 2 or the novel and familiar object in the novel object–recognition test. (**D** and **E**) Discrimination index (**D**) and distance traveled (**E**) for the novel over the familiar arm of the Y maze for spatial memory. (**F**–**H**) Number of errors before finding the target (**F**), deviation from the target at first error (**G**), and strategy used to find the target (**H**) in the Barnes maze. *n =* 15–18 *Kat6b^+/+^* (7–8M/8–10F) and 15–16 *Kat6b^+/–^* (8–9M/5–7F) mice per treatment group. Data are represented as mean ± SEM and were analyzed using 2-way (**A**, **B**, and **E**) or 3-way (**C**, **F**, **G**, and **H**) ANOVA with Holm-Šidák correction or 1-sample *t* test comparing with 0 (**D**). Circles, triangles, individual female and male mice. Related data in Supplemental Figure 17.

No difference was observed in ALCAR- versus vehicle-treated mice between genotypes in the rotor-rod test (Supplemental Figure 17A), the visual cliff (Supplemental Figure 17B), or motor functions in the open-field test (Supplemental Figure 17, C–I). Genotype effects persisted within both treatment groups in the elevated O maze (Figure 11, A and B, and Supplemental Figure 17J). Apart from this, ALCAR-treated *Kat6b^+/+^* WT mice spent 1.7-fold more time in the open arms of the elevated O maze than vehicle-treated *Kat6b^+/+^* controls (*P* = 0.0006; Figure 11A), suggesting an anxiolytic effect of ALCAR in this test.

In the novel object–recognition test and Y maze test of spatial reference memory, vehicle-treated *Kat6b^+/–^* mice failed to spend more time around the novel object or in the novel arm (Figure 11, C and D), as observed in previous cohorts (Figure 5, C, D, F, and G, and Figure 8, E and G). Notably, ALCAR-treated *Kat6b^+/–^* showed a clear preference for the novel object (*P* = 3 × 10^–6^; Figure 11C) and novel arm (*P* < 10^–6^; Figure 11D), similarly to control mice, while traveling a greater distance than control mice (Figure 11E). In the Barnes maze, vehicle-treated *Kat6b^+/–^* mice showed impaired spatial learning (Figure 11, F–H), as seen in the baseline assessment (Figure 5, J–L). Remarkably, ALCAR-treated *Kat6b^+/–^* mice displayed normal spatial search strategy learning behavior and performed similarly to vehicle-treated *Kat6b^+/+^* mice (Figure 11, F–H). Together, the restored preferences for the novel object and the novel Y maze arm and the restored search strategy learning and memory performance of the ALCAR-treated *Kat6b^+/–^* mice compared with the vehicle-treated *Kat6b^+/–^* mice suggest that ALCAR treatment improved learning and memory in *Kat6b^+/–^* mice.

**Figure 12:**
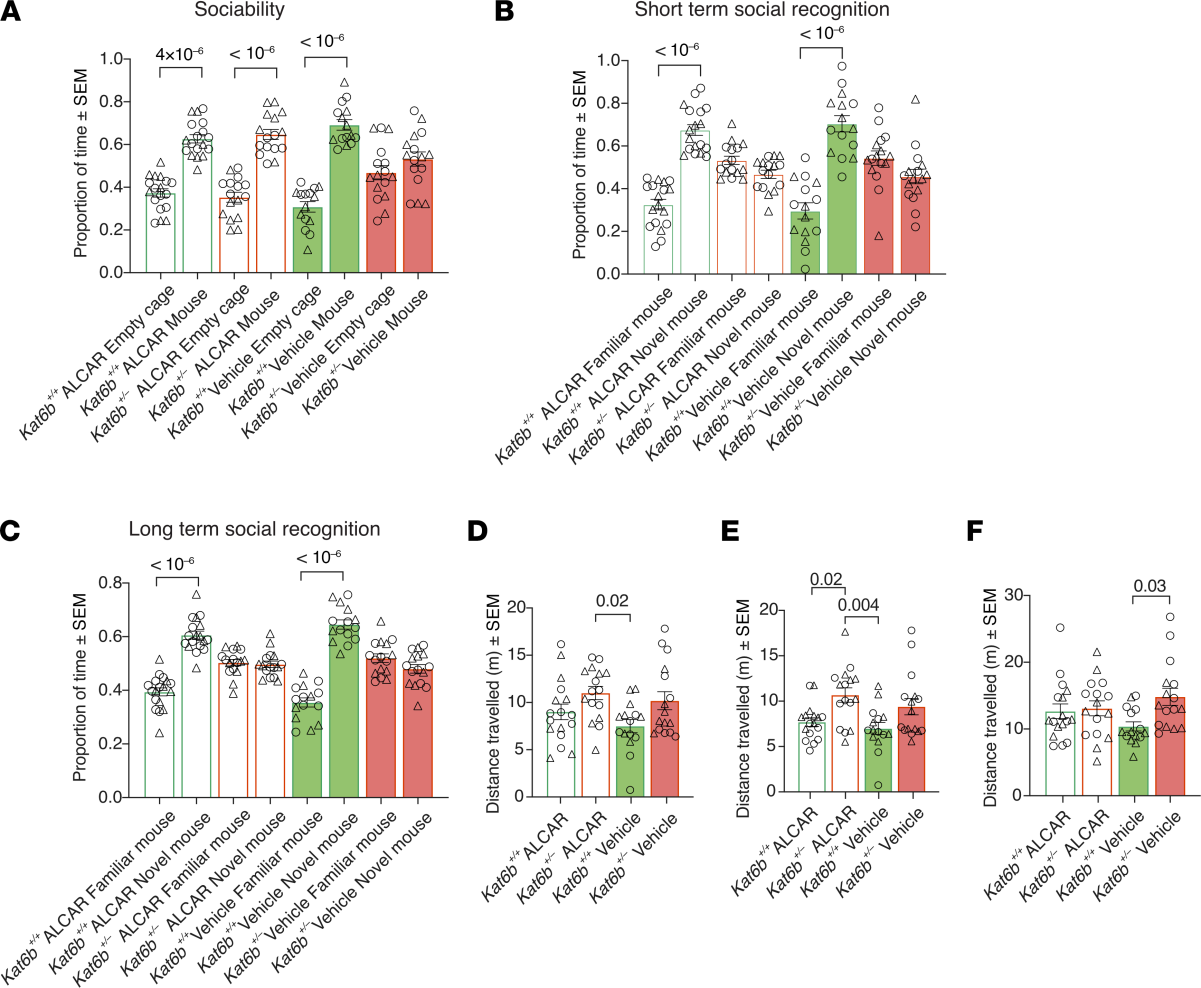
ALCAR improves sociability, but not social recognition in *Kat6b^+/–^* mice. (**A**–**F**) Three-chamber social test. Proportion of time spent around the empty cage and mouse (**A**). Proportion of time spent around the novel and familiar mouse (1-hour recall) (**B**). Proportion of time spent around the novel and familiar mouse (24-hour recall) (**C**). Total distance traveled in session 1 (**D**), session 2 (**E**), and session 3 (**F**). *n =* 15–18 *Kat6b^+/+^* (7–8M/8–10F) and 15–16 *Kat6b^+/–^* (8–9M/5–7F) mice per treatment group. Data are represented as mean ± SEM and were analyzed using 3-way (**A**–**C**) or 2-way (**D**–**F**) ANOVA with Holm-Šidák correction. Circles, triangles, individual female and male mice. Related data in Supplemental Figure 17.

In the 3-chamber social test, vehicle-treated *Kat6b^+/–^* mice failed to preference a mouse over an empty cage (Figure 12A), as seen previously (Figure 6, B and C, and Figure 9A). In contrast, ALCAR-treated *Kat6b^+/–^* mice showed a 1.9-fold preference for the mouse over an empty cage (*P =* 10^–6^; Figure 12A and Supplemental Figure 17K), indicating improved social interaction in this genotype. ALCAR had no effect on the short- or long-term social recognition defects of *Kat6b^+/–^* mice (Figure 12, B and C, and Supplemental Figure 17, L and M). ALCAR-treated *Kat6b^+/–^* mice traveled a greater distance than vehicle-treated *Kat6b^+/+^* mice (Figure 12, D–F).

### Effects of ALCAR and VPA treatment on gene expression in cortical neurons.

To investigate how KAT6B deficiency altered gene expression and how this in turn was affected by ALCAR and VPA treatment, we isolated *Kat6b^+/–^* and *Kat6b^+/+^* E16.5 cortical neurons as a cell type relevant to cognition and cultured them in 1 mM ALCAR, 1 mM VPA, or untreated medium (vehicle) for 4 days.

**Figure 13:**
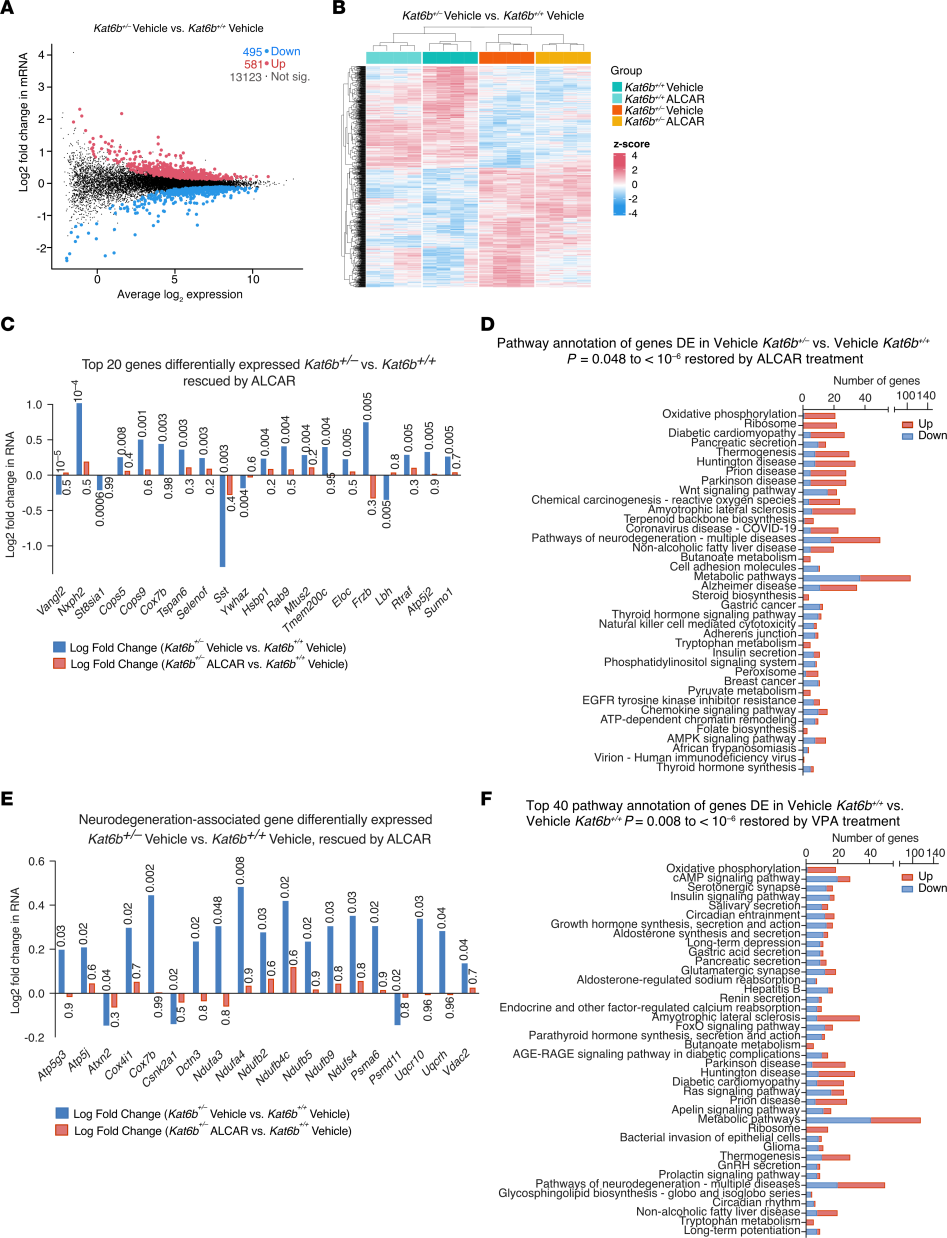
ALCAR and VPA treatment partially rescue expression changes in *Kat6b^+/–^* E16.5 cortical neurons. (**A**–**F**) RNA-Seq results of cortical neurons isolated from *Kat6b^+/–^* versus *Kat6b^+/+^* E16.5 mouse fetuses and cultured with 1 mM ALCAR, 1 mM VPA, or untreated medium (vehicle) for 4 days. *n =* 4 fetuses (2M/2F) per genotype. Data were analyzed as described in Supplemental Methods. FDR < 0.05 was used as transcriptome-wide significance cutoff. (**A**) Mean-difference plot of expressed genes comparing vehicle-treated *Kat6b^+/–^* and vehicle-treated *Kat6b^+/+^* neurons. Upregulated (red), downregulated (blue), not statistically significantly changed (black). (**B**) Heatmap of genes differentially expressed between vehicle-treated *Kat6b^+/–^* and vehicle-treated *Kat6b^+/+^* neurons. Results for ALCAR-treated *Kat6b^+/–^* and ALCAR-treated *Kat6b^+/+^* neurons are also shown. (**C** and **E**) Top 20 genes (**C**) and neurodegeneration-associated genes (**E**) differentially expressed in vehicle-treated *Kat6b^+/–^* versus vehicle-treated *Kat6b^+/+^* neurons (blue), but not differentially expressed in ALCAR-treated *Kat6b^+/–^* versus vehicle-treated *Kat6b^+/+^* neurons (red). FDRs for the comparison vehicle-treated *Kat6b^+/–^* versus vehicle-treated *Kat6b^+/+^* neurons are indicated above the bars. ALCAR-treated *Kat6b^+/–^* versus vehicle-treated *Kat6b^+/+^* neurons. *P =* 0.2–1.0. (**D** and **F**) KEGG pathway annotations of genes differentially expressed in vehicle-treated *Kat6b^+/–^* versus vehicle-treated *Kat6b^+/+^* neurons, but rescued in the ALCAR-treated *Kat6b^+/–^* versus vehicle-treated *Kat6b^+/+^* neurons (**D**) and in the VPA-treated *Kat6b^+/–^* versus vehicle-treated *Kat6b^+/+^* neurons (**F**). Number of affected genes in each pathway, downregulated genes (blue), upregulated genes (red). Related to data in Supplemental Figure 18.

Vehicle-treated *Kat6b^+/–^* E16.5 cortical neurons displayed 1,076 differentially expressed genes compared with vehicle-treated *Kat6b^+/+^* control cells (495 downregulated, 581 upregulated; Supplemental Table 4, Figure 13A, and Supplemental Figure 18A). ALCAR treatment drastically changed gene expression in *Kat6b^+/–^* and *Kat6b^+/+^* neurons (Figure 13B and Supplemental Figure 18, B and C). Notably, 431 of 1,076 (40%) genes that were differentially expressed in vehicle-treated *Kat6b^+/–^* versus vehicle-treated *Kat6b^+/+^* neurons were rescued by ALCAR treatment (Figure 13C and Supplemental Table 4). Only 7 of 431 rescued genes were overcompensated by ALCAR treatment. The rescued genes were enriched in Kyoto Encyclopedia of Genes and Genomes (KEGG) pathways, including oxidative phosphorylation, ribosome, several neurodegenerative diseases, and metabolic pathways (Figure 13D and Supplemental Table 5). Importantly, of the 50 genes differentially expressed in vehicle-treated *Kat6b^+/–^* versus vehicle-treated *Kat6b^+/+^* E16.5 cortical neurons (FDR = 0.048 to 0.0009) annotated with the term “Pathways of neurodegeneration — multiple diseases,” 19 were not differentially expressed in ALCAR-treated *Kat6b^+/–^* versus vehicle-treated *Kat6b^+/+^* neurons (*P =* 0.3 to 1.0; Figure 13E, Supplemental Table 4, and Supplemental Table 5). The rescued genes in “Pathways of neurodegeneration — multiple diseases” displayed a large overlap (13 of 19 genes) with genes in the pathway “Oxidative phosphorylation” (Supplemental Figure 18D), consistent with restored ATP output in SBBYSS HEK293T cells following ALCAR or l-carnitine treatment (Figure 10L).

In the VPA versus vehicle data set, VPA had a very strong effect on gene expression in *Kat6b^+/–^* and *Kat6b^+/+^* E16.5 cortical neurons (Supplemental Figure 18, F–J, and Supplemental Table 6). VPA treatment rescued 516 of 1,150 (45%) genes differentially expressed in vehicle-treated *Kat6b^+/–^* versus vehicle-treated *Kat6b^+/+^* neurons (Supplemental Table 6). Of these, 154 (30%) were overcompensated after VPA treatment, suggesting that the VPA dose could have been reduced. VPA treatment of *Kat6b^+/–^* neurons restored 81 of 130 (62%) KEGG pathway annotations enriched in genes differentially expressed in vehicle-treated *Kat6b^+/–^* versus vehicle-treated *Kat6b^+/+^* to normal (Figure 13F and Supplemental Table 7). The pathway annotation rescued by VPA overlapped with that rescued by ALCAR treatment, including oxidative phosphorylation, ribosome, several neurodegenerative diseases, and metabolic pathways (Figure 13F and Supplemental Table 7). In addition, VPA treatment rescued genes with neuronal function annotations that were not rescued by ALCAR, including serotonergic synapse, circadian entrainment, long-term depression, and glutaminergic synapse (Supplemental Table 7). Overall, both ALCAR and VPA treatment partially rescued gene expression changes observed in *Kat6b^+/–^* versus *Kat6b^+/+^* neurons.

## Discussion

In this study, we showed that *Kat6b^+/–^* mice display behavioral anomalies resembling certain aspects of the human KAT6B deficiency syndrome, SBBYSS, including learning difficulties and autism-like presentation. Additionally, we provided an assessment of *KAT6B* mRNA abundance and histone acetyltransferase function for a range of *KAT6B* variants in human cells. Our observation that *KAT6B* mRNA levels were reduced in cells with all SBBYSS mutations except for those in the final exon 18 is congruent with previous assessments of 2 proximal *KAT6B* mutations (26, 27) and 4 mutations in the final exon (27), together suggesting that KAT6B variants outside the final exon undergo nonsense-mediated decay. In the absence of reliable antibodies against KAT6B, we cannot verify whether abnormal KAT6B protein is produced in our *KAT6B* mutant HEK293T cell lines. While most N-terminal and central mutations, but not C-terminal SBBYSS mutations, affected acetylation levels, only 3 SBBYSS mutations affected mitochondrial function. Of note, these observations were made in HEK293T cells and the effects of KAT6B mutations may differ in a cell type–specific manner.

We observed a greater effect on H3K9ac than H3K23ac in HEK293T cells carrying SBBYSS mutations and in *Kat6b* mutant mouse tissues. This suggests that KAT6B may predominantly acetylate H3K9, with a lesser role in the acetylation of H3K23. Alternatively, there may be a greater capacity for compensation of KAT6B-mediated acetylation of H3K23, for example, by KAT6A, which has been shown to acetylate this mark (28, 29). KAT6B and KAT6A share protein complex members (13), which may become available to form more KAT6A complexes when KAT6B levels are reduced.

In the *Kat6b^+/–^* cortex compared with controls, a reduction in H3K9ac was only observed during development at E18.5 and P14, but not in the P21 or adult cortex. In contrast, adult *Kat6b^+/–^* WBCs still displayed a reduction in H3K9ac. The absence of a detectable difference in the more mature brain tissue may be due to the cellular complexity of the cortex, which might mask any differences that might exist. Indeed, we only were able to detect differences in WBCs when we separately assessed individual cell types. In the bulk WBC population, those differences were masked.

Curiously, we also observed an increase in H3K14ac levels in *KAT6B* mutant HEK293T cell lines with a decrease in H3K9ac. H3K14ac is catalyzed by KAT7 (30, 31), another MYST family histone acetyltransferase. KAT7 shares protein complex members with KAT6B (13). *KAT6B* mutations lowering KAT6B protein levels could make critical complex members available, which might enable KAT7 to elevate H3K14ac levels.

We did not observe an effect on mRNA abundance or global histone acetylation in cells with SBBYSS mutations in the last exon. While global acetylation may not be affected by these mutations, there may be locus-specific effects not detectable by immunoblotting. Disruption of the C-terminal serine/methionine-rich domain of KAT6B, which is predicted to bind to transcription factors based on in vitro studies in Sf9 insect cells (32), may cause KAT6B to fail to localize to its target genes. This could have detrimental consequences without affecting global acetylation levels at H3K9 or H3K23.

Our *Kat6b^+/–^* mice exhibited not only the histone acetylation deficits of human cells carrying SBBYSS-specific *KAT6B* mutations, but also modeled some of the cognitive and social deficits of individuals with SBBYSS as well as hearing and vocalization defects, which mirror individuals with KAT6B disorders, who present with hearing and speech deficits (2, 16). The underlying cause for these vocalization differences may be structural, with defects in the jaw, tongue, or larynx or impaired innervation of these structures, suggested by shorter lower jaws in *Kat6b^–/–^* mice and *Kat6b^gt/gt^* mice (6). Alternatively, these differences may be behavioral. Maternal separation–induced USVs rely on separation being a sufficient stressor to induce vocalization. As adult *Kat6b^+/–^* mice demonstrated reduced anxiety in the open field and elevated O maze, maternal separation may not have been sufficiently stressful to induce vocalizations in *Kat6b^+/–^* pups.

The learning and memory defects observed here in *Kat6b^+/–^* mice are consistent with learning defects in individuals with KAT6B disorders (2, 16). The lack of appropriate anxiety in *Kat6b^+/–^* mice is not a commonly described trait in individuals with SBBYSS; however, some individuals with GPS present with increased anxiety and aggression (3). As GPS has been proposed to result from gain-of-function mutations in *KAT6B*, reduced anxiety levels in a loss-of-function mouse model could be relevant. It is also possible that the reduced anxiety observed in *Kat6b^+/–^* mice is due to a broader cognitive impairment, such that these mice may fail to recognize the risks associated with open areas. Finally, the sociability and social-recognition deficits in *Kat6b^+/–^* mice mirror autism-like traits observed in some individuals with KAT6B disorders, including limited social interactions, impaired communication, restricted interests, and difficulty in sensory processing (17).

Both VPA and ALCAR treatment increased histone acetylation in WBCs and cortex, restored a subset of gene expression changes, and excitingly, improved some of the behavioral deficits observed in *Kat6b^+/–^* mice. This suggests that treatments to ameliorate the condition of individuals with *KAT6B* heterozygous mutations may be possible and that histone acetylation in WBCs could serve as a biomarker. While VPA and ALCAR elevated histone acetylation levels, they had only partially overlapping effects on behavior. In this context, it is important to note that, in addition to affecting histone acetylation levels, VPA and ALCAR have been reported to have other functions (some of which will be discussed below), such that it is not surprising that their effects did not fully overlap.

The failure of VPA treatment to rescue the spatial reference memory defect in *Kat6b^+/–^* mice and its negative effects on the spatial reference memory performance of WT mice add to conflicting reports of the effects of VPA on cognition in rodents. Long-term treatment with VPA has been variably reported to cause no negative effects on memory in rodents (33, 34) or impaired performance in learning and memory tests (35–37). Furthermore, a subset of individuals receiving VPA to prevent seizures have been reported as displaying cognitive impairments and memory deficits (23, 38), which were reverted upon VPA cessation (39).

In addition to inhibiting HDACs, VPA can elevate GABA levels within the brain. Mice deficient in KAT6B have a reduction in the number of GABAergic neurons (6), and treatment of rats with a GABA-A receptor antagonist has been shown to decrease social behavior (40). It is possible that elevating GABA via VPA treatment may contribute to the improved sociability and social recognition in *Kat6b^+/–^* mice.

VPA also affects serotonin signaling, with both increased (41) and reduced (42) serotonin levels found in the brains of rats treated with VPA in utero. Notably, defects in sociability and early development were observed in mice lacking the serotonin biosynthesis enzyme TPH2 (43). If VPA elevated serotonin signaling, this could possibly improve sociability in *Kat6b^+/–^* mice.

Our finding that ALCAR treatment improved learning and memory in *Kat6b^+/–^* mice is congruent with ALCAR treatment of individuals with autism-spectrum disorders improving cognition (44). Individuals with Rett syndrome achieved a modest improvement in communication after treatment with l-carnitine (45), the nonacetylated form of ALCAR. l-carnitine treatment also caused a modest improvement in cognitive function in a mouse model of Rett syndrome (46).

The lack of appropriate anxiety observed in ALCAR-treated *Kat6b^+/+^* and *Kat6b^+/–^* mice is consistent with observed anxiolytic effects of ALCAR in rodents (47). While the molecular underpinning of ALCAR as an anxiolytic is not well understood, ALCAR has been proposed to cause antidepressant effects through epigenetic induction of a glutamatergic receptor (48), l-carnitine can increase levels of neurotransmitters such as noradrenaline and serotonin in the cortex (49), and ALCAR treatment in rats has been shown to increase levels of serotonin and serotonin metabolites in the brain (49).

Additionally, ALCAR can contribute an acetyl group to acetylcholine (50). Consistent with our observations of restored performance in learning and memory tasks in ALCAR-treated *Kat6b^+/–^* mice, acetylcholine is implicated in learning and memory, with increased acetylcholine levels associated with hippocampal-dependent learning (51).

Finally, l-carnitine can contribute to GABA synthesis (52). Hence, similarly to VPA, ALCAR may improve the sociability defect of *Kat6b^+/–^* mice through elevated GABA neurotransmission. However, unlike VPA, ALCAR did not improve social recognition in *Kat6b^+/–^* mice, suggesting that brain regions and pathways regulating social recognition may be modulated by VPA, but not ALCAR.

Although we did not observe a difference in mitochondrial function in E16.5 cortical neurons, we observed increased expression in a number of genes associated with mitochondrial function, suggesting that the functional assays may be less sensitive than RNA-Seq. VPA and ALCAR treatment restored expression of mitochondrial function genes and neurodegeneration genes differentially expressed between *Kat6b^+/–^* and *Kat6b^+/+^* samples to normal, suggesting that treatment may be beneficial.

We treated mice from early postnatal life and throughout behavioral testing under the assumption that continued treatment was required for continued beneficial outcomes. Consistent with this hypothesis, inhibition of HDACs with sodium butyrate in a mouse model of Alzheimer’s disease improved contextual memory, which returned to baseline following treatment withdrawal (53). While long-term treatment with the HDAC inhibitor vorinostat is well tolerated (54), it remains to be determined whether treatment restricted to a critical window of development would produce self-sustaining neural changes and confer beneficial outcomes.

Although VPA and ALCAR affect mechanisms other than histone acetylation levels, as discussed above, other compounds affecting histone acetylation levels in mouse models have shown promising results, suggesting that effects on histone acetylation levels may be relevant. HDAC inhibition with vorinostat in mice deficient in the H3K36 methyltransferase ASHL1, variants of which are associated with a high risk of autism spectrum disorder, saw improvements in sociability and memory (55). In a mouse model of Kabuki syndrome, resulting from deficiency in the H3K4 lysine methyltransferase KMT2D, HDAC inhibition with AR-42 rescued structural and functional brain deficits (56). In mice heterozygous for *Crebbp*, the gene mutated in Rubinstein-Taybi syndrome (57), HDAC inhibition with trichostatin A (TSA) rescued defective neurogenesis in cortical precursors (58), and vorinostat treatment improved long-term potentiation and fear-conditioning memory defects (59). In mice hemizygous for *Mecp2*, the gene implicated in Rett syndrome, HDAC inhibition with tubastatin improved exploratory behavior (60). Collectively, these reports highlight that compounds increasing histone acetylation may be useful for the treatment of chromatin-mediated intellectual-disability disorders. Importantly, this includes disorders not specifically resulting from mutations in acetyltransferase genes. This suggests that elevating histone acetylation levels may be beneficial in treating a range of disorders arising from mutations in chromatin-modifier genes, thereby alleviating the need to develop targeted activators or inhibitors of individual proteins affected in congenital intellectual-disability disorders.

In conclusion, we found ALCAR treatment restored learning and memory and improved sociability in *Kat6b^+/–^* mice without negatively affecting motor skills or other behavioral parameters. In contrast, while VPA improved the sociability and short-term social recognition of *Kat6b^+/–^* mice, its negative effects on learning and memory in *Kat6b^+/+^* mice and motor function in both genotypes are concerning. It therefore seems that ALCAR might be useful for the treatment of KAT6B deficiency, whereas VPA treatment would require careful consideration and dosage adjustment. One important caveat to these findings is that not all *KAT6B* variants may be loss-of-function mutations. Indeed, as mentioned in the Introduction, individuals with GPS may carry mutations that might cause a gain of an abnormal or dominant negative KAT6B protein function. The assessment of SBBYSS-causing mutations in HEK293T cells here showed reduced *KAT6B* mRNA and histone acetylation levels in cells with mutations in various exons, but not in the final exon. For individuals with mutations in the final exon, agents that augment histone acetylation may not present a treatment option and may even worsen the effects of their *KAT6B* mutation. The identified mitochondrial impairment in cells carrying some SBBYSS mutations suggests that some individuals with *KAT6B* mutations may additionally benefit from mitochondria-supporting treatment. Hence, while this study suggests that ALCAR treatment might be of benefit for individuals with KAT6B disorders for the improvement of memory and sociability, our functional studies of individual human mutations have shown that the functional consequences of each *KAT6B* variant need to be assessed individually.

## Methods

### Sex as a biological variable

Male and female mice were used as they became available in the breeding colony and in litters of embryos and fetuses recovered. Numbers of male and female mice are stated in the figure legends and depicted with triangles and circles for male and female mice, respectively, for behavioral tests. The human cells used, HEK293T cells, are female.

### Human cell culture and generation of SBBYSS-causing gene mutations

HEK293T cells were supplied by M. Herold (Walter and Eliza Hall Institute of Medical Research). SBBYSS-causing mutations were generated in HEK293T cells using CRISPR/Cas9 and homology direct repair. Details can be found in Supplemental Methods.

### Mice

#### Kat6b-null allele.

The *Kat6b* locus was targeted twice (Supplemental Figure 5, A–C) to flank *Kat6b* exon 2 and exons 11 to 12 with *loxP* sites (*Kat6b^e2fl&e11–12–fl^*). *Kat6b^e2–fl&e11–12–fl^* mice were crossed to a Cre-deleter strain (61), resulting in the Cre-recombinase–mediated deletion of exons 2–12 of the *Kat6b* gene, creating a *Kat6b*-null allele (*Kat6b^–^*). Mice were backcrossed to C57BL/6 mice for more than 10 generations and genotyped by PCR using primers displayed in Supplemental Table 8.

### Behavioral tests

Detailed descriptions of the behavioral tests and statistical tests employed for each test are in the Supplemental Methods. All mice, including outliers, were included in assessment of behavioral paradigms. Male mice are depicted with a triangle and female mice with a circle in all behavioral test graphs. Measurements of distance were assessed using TopScanLite Basic RealTime Option, version 2.00, tracking software (CleverSys Inc).

### Treatment of mice

#### VPA.

From ages 2 to 4 weeks, mice were given 100 mg per kg body weight VPA sodium salt (P4543, MilliporeSigma) twice daily by oral gavage or an equivalent volume of vehicle (H_2_O) only. From age 4 weeks and throughout behavioral testing, mice were given chow powder supplemented with 20 g/kg VPA sodium salt made into a mash with H_2_O or mash without VPA.

#### ALCAR.

From ages 2 to 4 weeks, mice were given 50 mg per kg body weight *O*-acetyl-*l*-carnitine hydrochloride (ALCAR) (A6706, MilliporeSigma) twice daily by oral gavage or an equivalent volume of vehicle (H_2_O) only and from age 4 weeks, chow powder supplemented with 2 g/kg ALCAR made into a mash with H_2_O or mash without ALCAR.

### Statistics

RNA-Seq data were analyzed as described in Supplemental Methods. Other data were analyzed using GraphPad Prism, version 8.3.1, for Mac (GraphPad Software). In all figures, each circle or triangle represents a clonal HEK293T cell line or an individual mouse. All data are represented as mean ± SEM unless otherwise stated. The statistical analyses are stated in the figure legends. *P* < 0.05 and FDR < 0.05 were considered significant.

Detailed methods and materials are described in Supplemental Methods.

### Study approval

Mouse experiments were conducted in accordance with the Australia Code of Practise for the Care and Use of Animals for Scientific Purposes and with the approval of the Walter and Eliza Hall Institute Animal Ethics Committee.

### Data availability

The RNA-Seq data have been deposited in the NCBI’s Gene Expression Omnibus database (GEO GSE249964). Values for all data points in graphs are reported in the Supporting Data Values file.

## Author contributions

MIB carried out experiments, performed data analyses, and drafted the manuscript. HKV and NDG carried out experiments and performed data analyses. ALG performed RNA-Seq data analysis, supervised by GKS. SE, APV, KLR, MEB, and AJH provided feedback on designing and performing experiments. AKV and TT conceived the project, designed experiments, performed data analyses, and drafted the manuscript. All authors read and contributed to the manuscript.

## Supplementary Material

Supplemental data

Unedited blot and gel images

Supplemental table 2

Supplemental table 4

Supplemental table 5

Supplemental table 6

Supplemental table 7

Supporting data values

## Version 1 04/01/2024

Electronic publication
