# Supplementary material for: Increasing histone acetylation improves sociability and restores learning and memory in KAT6B-haploinsufficient mice
Source: J Clin Invest. 2024 Apr 1;134(7):e167672. doi: 10.1172/JCI167672 (PMC10977983; doi:10.1172/JCI167672)
Supplement: Supplemental data [file jci-134-167672-s087.pdf]

Supplemental Material

Increasing histone acetylation improves sociability and restores learning and memory in *KAT6B*-haploinsufficient mice

Maria I. Bergamasco[1,2], Alexandra L. Garnham[1,2], Hannah K Vanyai[1,2], Niall D. Geoghegan[1,2], Adam P. Vogel[3], Samantha Eccles[1,2], Kelly L. Rogers[1,2], Gordon K. Smyth[1,4], Marnie E. Blewitt[1,2], Anthony J. Hannan[5,6], Tim Thomas[1,2,*] and Anne K. Voss[1,2,*]

[1]*The Walter and Eliza Hall Institute of Medical Research, Melbourne, Victoria, Australia*

[2]*Department of Medical Biology, University of Melbourne, Melbourne, Victoria, Australia*

[3]*Centre for Neurosciences of Speech, University of Melbourne, Melbourne, Victoria, Australia*

[4]*School of Mathematics and Statistics, University of Melbourne, Parkville, Victoria, Australia.*

[5]*Florey Institute of Neuroscience and Mental Health, Melbourne Brain Centre, University of Melbourne, Parkville, Victoria, Australia.*

[6]*Department of Anatomy and Neuroscience, University of Melbourne, Parkville, Victoria, Australia.*

18 Supplemental Figures

Supplemental Tables 1, 3 and 8-11 supplied here

Supplemental Tables 2 and 4-7 supplied as Excel files

Supplemental Methods

Supplemental Figures

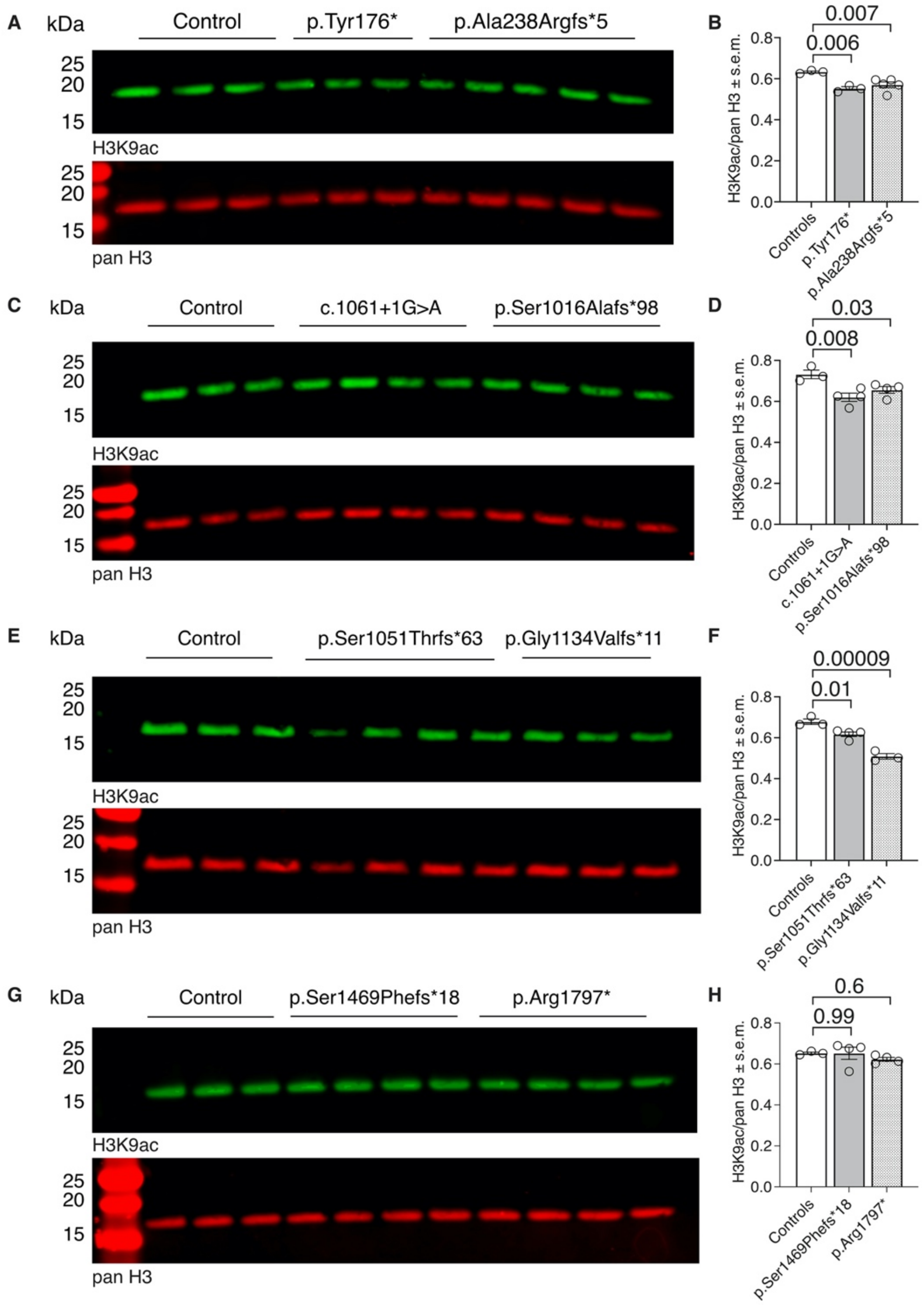

Supplemental Figure 1. Effect of SBBYSS mutations on H3K9 acetylation in HEK293T cells

(A-H) Western immunoblot (A,C,E,G) and quantitation (B,D,F,H) of H3K9ac and pan H3 of KAT6B control HEK293T cells or cells carrying the following SBYYSS-causing mutations: (A,B) c.527dup p.Tyr176* and c.708-709delTT p.Ala238Argfs*5,

(C,D) splice site mutation c.1061+1G>A and c.3046del p.Ser1016Alafs*98,

(E,F) c.3152delG p.Ser1051Thrfs*63 and c.3401delG p.Gly1134Valfs*11,

(G,H) c.4405dupT p.Ser1469Phefs*18 and c.5389C > T p.Arg1797*.

N = Histones extracts derived from 3-5 individual HEK293T clonal cell lines per SBBYSS mutation. Each western immunoblot lane represents a single clonal cell line. 500 ng acid extracted protein was loaded per lane. Data are displayed as mean ± s.e.m. and were analysed using a one-way ANOVA followed by a Holm-Sidak post-hoc correction for multiple testing.

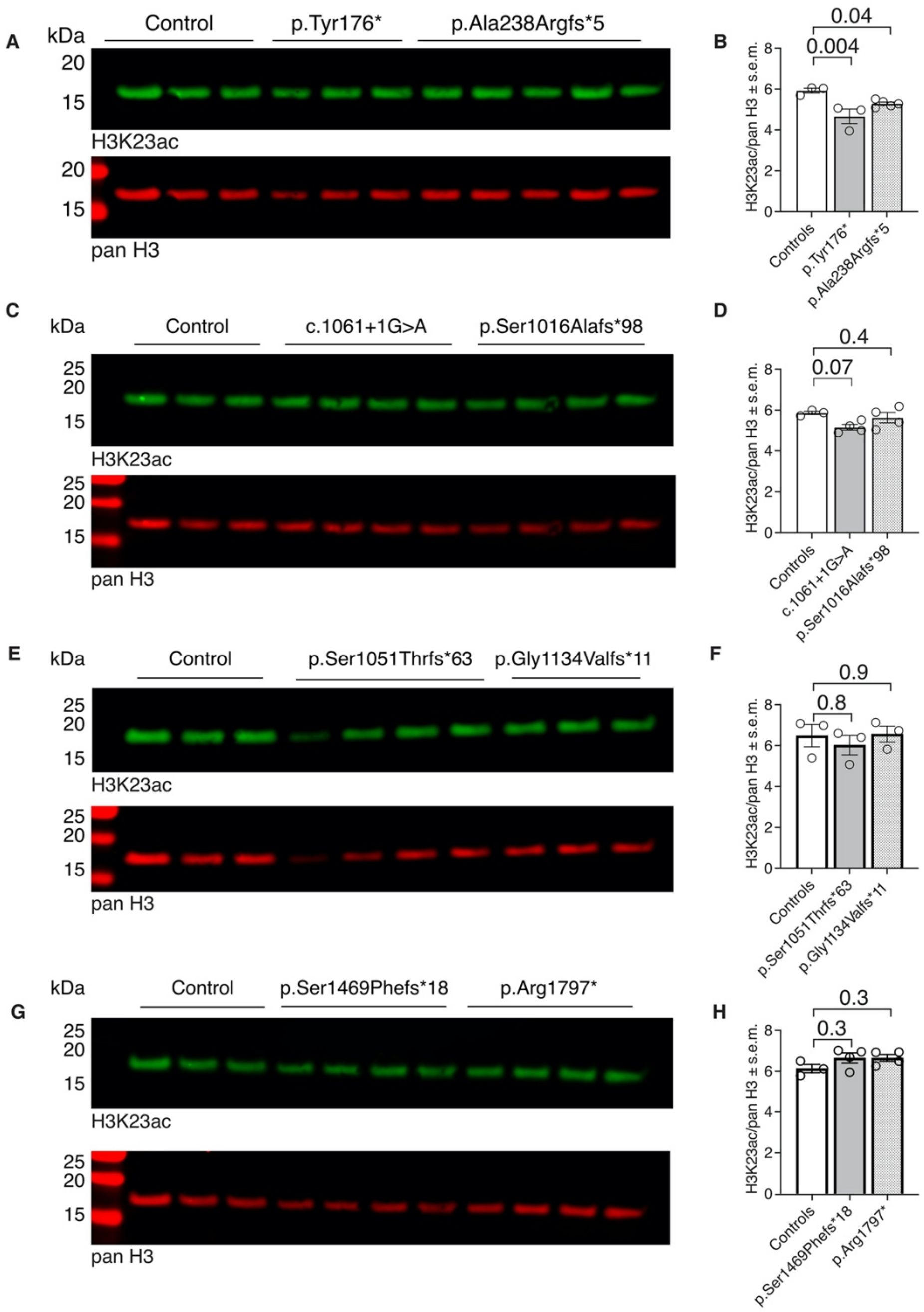

Supplemental Figure 2. Effect of SBBYSS mutations on H3K23 acetylation in HEK293T cells

(A-H) Western immunoblots (A,C,E,G) and quantitation (B,D,F,H) of H3K23ac and pan H3 of KAT6B control HEK293T cells or cells carrying the following SBYYSS-causing mutations:

(A,B) c.527dup p.Tyr176* and c.708-709delTT p.Ala238Argfs*5,

(C,D) splice site mutation c.1061+1G>A and c.3046del p.Ser1016Alafs*98,

(E,F) c.3152delG p.Ser1051Thrfs*63 and c.3401delG p.Gly1134Valfs*11,

(G,H) c.4405dupT p.Ser1469Phefs*18 and c.5389C > T p.Arg1797*.

N = Histones extracts derived from 3-5 individual HEK293T clonal cell lines per SBBYSS mutation. Each western immunoblot lane represents a single clonal cell line. 200 ng acid extracted protein was loaded per lane. Data are displayed as mean ± s.e.m. and were analysed using a one-way ANOVA followed by a Holm-Sidak correction.

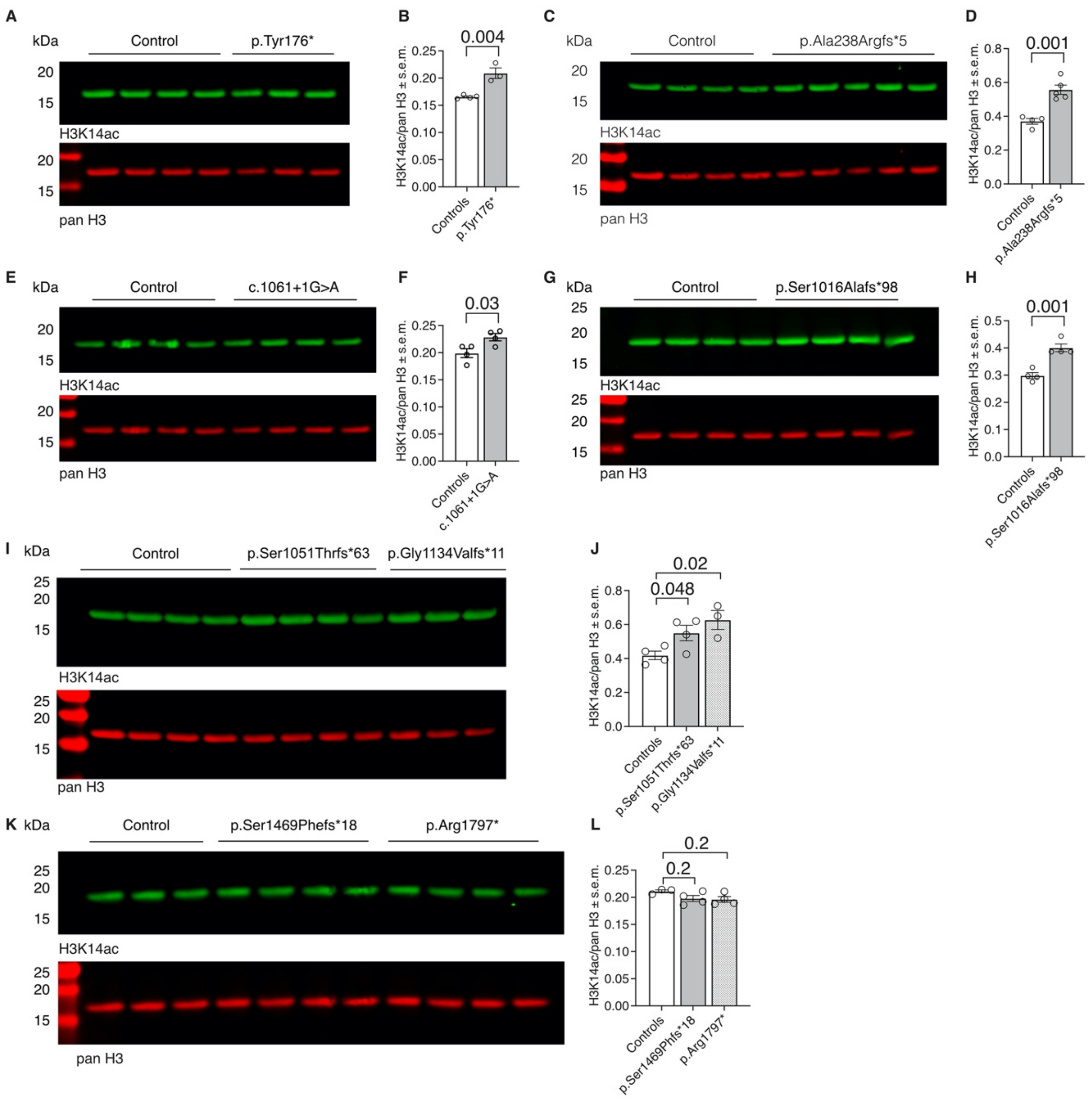

Supplemental Figure 3. Effect of SBBYSS mutations on H3K14 acetylation in HEK293T cells

(A-L) Western immunoblots (A,C,E,G,I,K) and quantitation (B,D,F,H,J,L) of H3K14ac and pan H3 of KAT6B control HEK293T cells or cells carrying the following SBYYSS mutations:

(A,B) c.527dup p.Tyr176*,

(C,D) c.708-709delTT p.Ala238Argfs*5,

(E,F) splice site mutation c.1061+1G>A

(G,H) c.3046del p.Ser1016Alafs*98,

(I,J) c.3152delG p.Ser1051Thrfs*63 and c.3401delG p.Gly1134Valfs*11,

(K,L) c.4405dupT p.Ser1469Phefs*18 and c.5389C > T p.Arg1797*.

N = Histones extracts derived from 3-5 individual HEK293T clonal cell lines per SBBYSS mutation. Each western immunoblot lane represents a single clonal cell line. 2 μg acid extracted protein was loaded per lane. Data are displayed as mean ± s.e.m. and were analysed using a Student's t-test (B,D,F,H) or one-way ANOVA with Holm-Sidak correction (J,L).

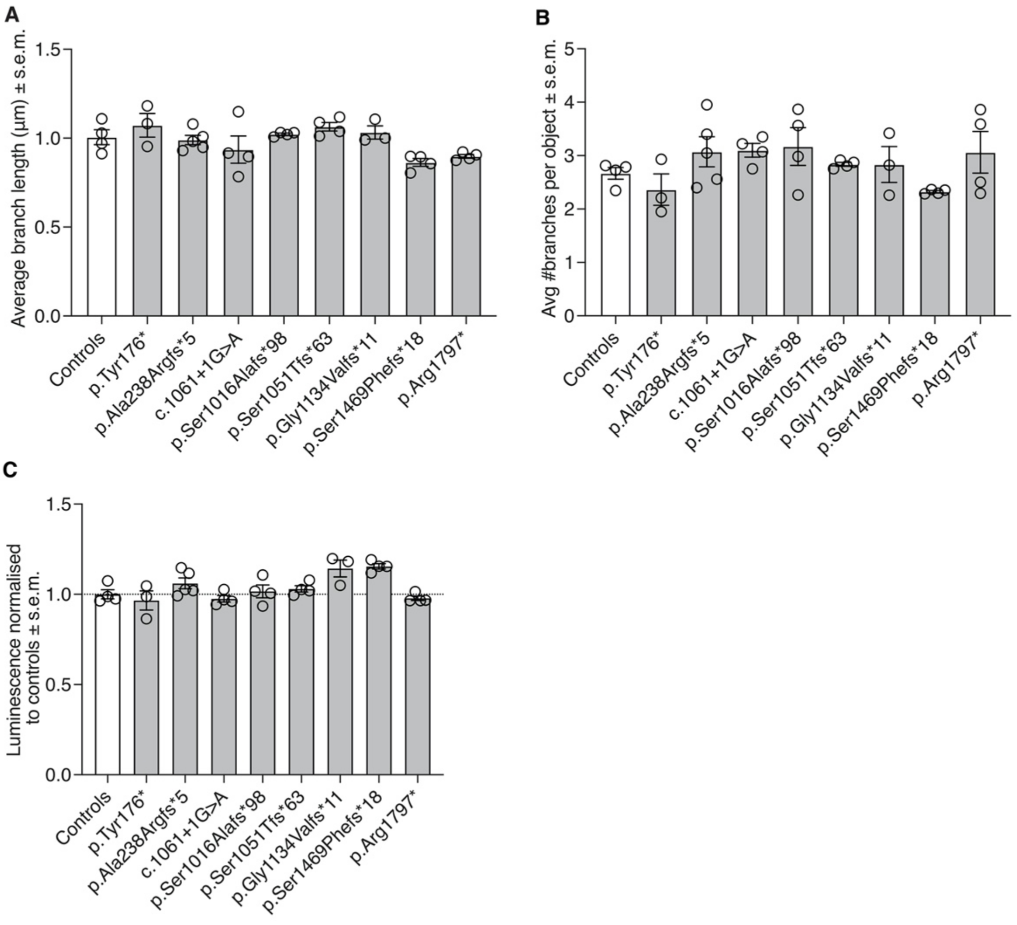

Supplemental Figure 4: Effect of SBBYSS mutations on mitochondrial morphology and function in HEK293T cells

(A-B) Average mitochondrion branch length and number of branches per mitochondrion in control HEK293T cells or cells carrying individual SBBYSS mutations assessed on confocal microscopy images of MitoTracker stained cells.

(C) ATP levels as assessed by Mitochondrial ToxGlo assay in control and HEK293T cells carrying SBBYSS mutations normalised to controls.

Each circle represents the average of 30-40 cells of an individual clone (A,B,C). N = 3-5 clones per mutation. Data in all graphs presented as mean ± s.e.m. and analysed using a one-way ANOVA with Holm-Sidak correction.

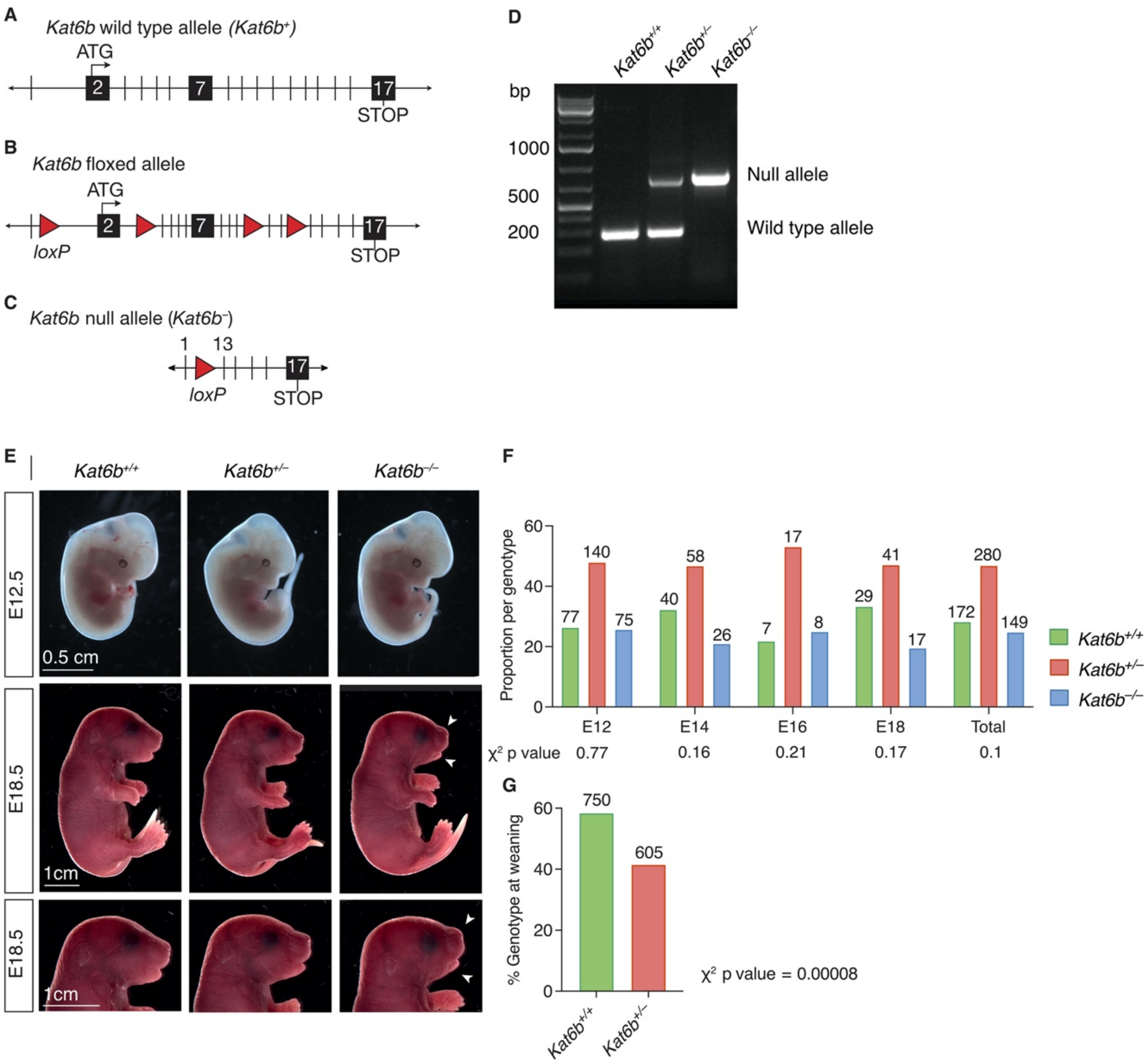

Supplemental Figure 5. Mouse wild-type, conditional and null *Kat6b* alleles

(A-C) Depiction of the *Kat6b* wild type and mutant alleles with exon structure. The *Kat6b* wild-type allele (*Kat6b*$^{+}$) (A) was double targeted by homologous recombination to introduce *loxP* sites flanking exon 2 and exons 11 - 12 (B). *Cre*-mediated recombination removed exons 2 - 12, comprising 130 kb of DNA, and yielded the *Kat6b* null allele (*Kat6b*$^{-}$) (C), lacking the coding sequence for the initiating methionine, the NEMM domain, PHD domains, catalytic MYST domain and a portion of the acidic domain. Any protein product produced would lack acetyltransferase activity and would be out of frame, thereby lacking any resemblance to the KAT6B protein.

(D) Representative PCR genotyping DNA gel of *Kat6b*$^{+/+}$, *Kat6b*$^{+/-}$ and *Kat6b*$^{-/-}$ samples.

(E) Representative images of *Kat6b*$^{+/+}$, *Kat6b*$^{+/–}$ and *Kat6b*$^{–/–}$ E12.5 mouse embryos and E18.5 foetuses. The heads of E18.5 foetuses are magnified to show the upper and lower jaw of the *Kat6b*$^{–/–}$ mutant foetus (white arrows). Scale bar = 1 cm.

(F) Percentages of *Kat6b*$^{+/+}$, *Kat6b*$^{+/–}$ and *Kat6b*$^{–/–}$ embryos and foetuses recovered at E12.5, E14.5, E16.5 and E18.5. The number of mice per genotype at each time point is shown above each bar. Pearson's Chi squared test p-values for the comparison of observed numbers to the expected numbers are shown below each time point.

(G) Percentage of genotypes of at weaning (3 weeks of age) in offspring of *Kat6b*$^{+/–}$ by wild-type matings. The number of mice per genotype is shown above each bar. *Kat6b*$^{+/–}$ mice are significantly underrepresented compared to controls ($p = 0.00008$) based on the Pearson's Chi squared test for the comparison of observed to expected numbers.

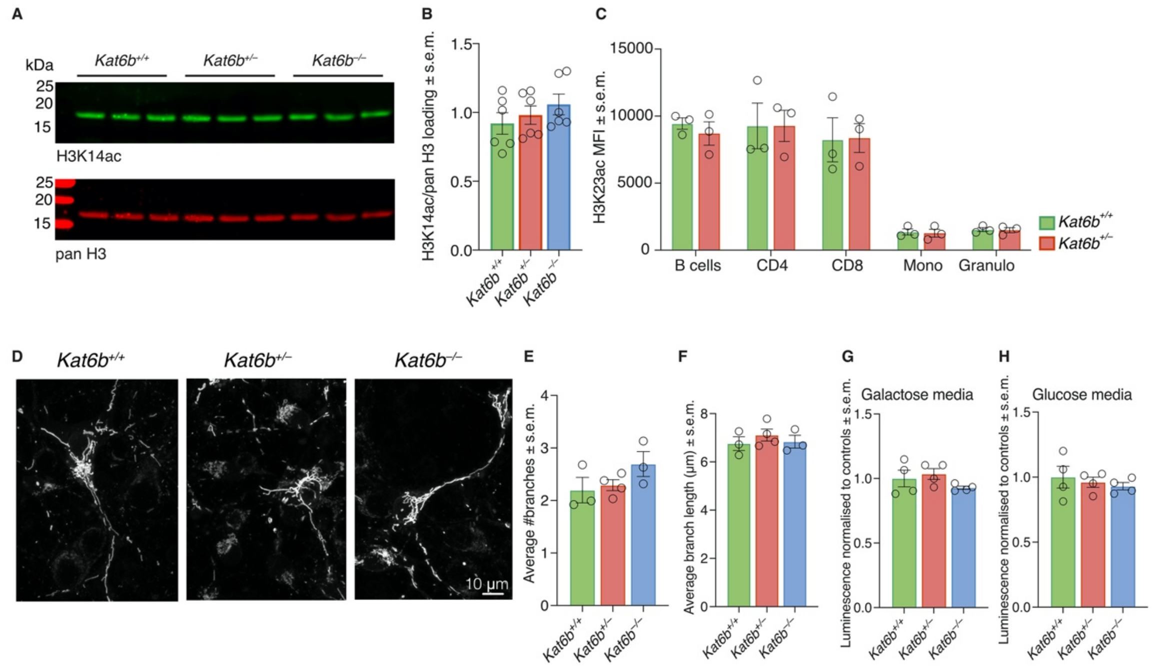

Supplemental Figure 6. Effect of *Kat6b* gene status on H3K14ac in the E18.5 mouse cortex, H3K23ac in mouse peripheral blood and mitochondrial function in E16.5 cortical neurons

(A,B) Representative Western immunoblot (A) and quantitation (B) of H3K14ac and pan H3 in E18.5 cortex of *Kat6b*$^{+/+}$, *Kat6b*$^{+/-}$ and *Kat6b*$^{-/-}$ foetuses. Each lane represents an individual foetus. 2 µg acid extracted protein was loaded per sample.

(C) Median fluorescence intensity (MFI) of H3K23ac in peripheral blood cells of adult *Kat6b*$^{+/-}$ and *Kat6b*$^{+/+}$ mice.

(D) Representative max projections of confocal microscopy images of Mitotracker-labelled mitochondria in E16.5 cortical neurons from *Kat6b*$^{+/+}$, *Kat6b*$^{+/-}$ and *Kat6b*$^{-/-}$ foetuses. Scale bar = 10 µm.

(E-F) Average number of branches (E) and branch length (F) of mitochondria in E16.5 cortical neurons from *Kat6b*$^{+/+}$, *Kat6b*$^{+/-}$ and *Kat6b*$^{-/-}$ foetuses.

(G-H) ATP levels as assessed by Mitochondrial ToxGlo assay of E16.5 cortical neurons from *Kat6b*$^{+/+}$, *Kat6b*$^{+/–}$ and *Kat6b*$^{–/–}$ foetuses grown for 24 h in galactose (G) or glucose-based medium (H).

N = 6 mice (B), 3 mice (C) and 3-4 mice (E-H) per genotype. Each circle represents an individual mouse (B,C,E-H). Data are presented as mean ± s.e.m. and were analysed using a one-way ANOVA with Holm-Sidak correction (B,E,F,G,H) or data were log transformed and analysed using t-tests (C).

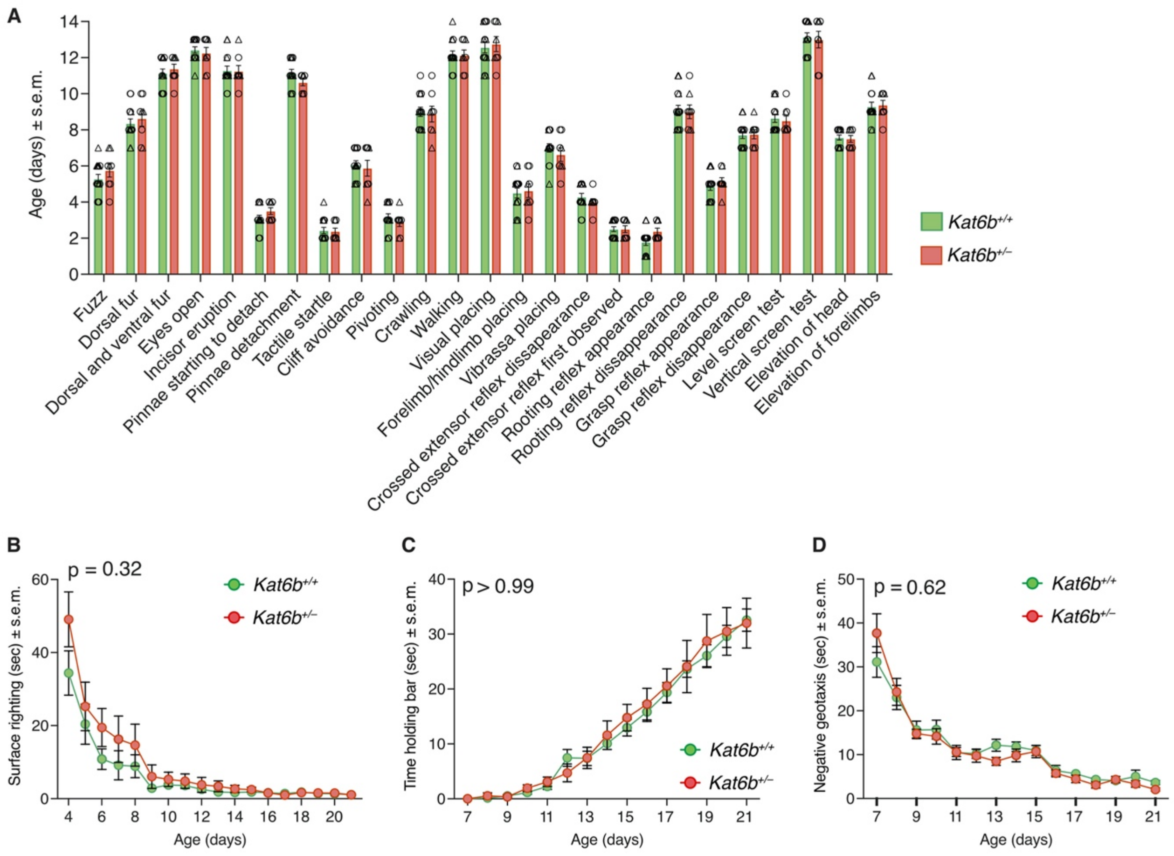

Supplemental Figure 7. Basic behavioural and physical milestones in *Kat6b*$^{+/+}$ and *Kat6b*$^{+/–}$ pups

(A) Age at which basic phenotypic and behavioural milestones were observed in *Kat6b*$^{+/+}$ and *Kat6b*$^{+/–}$ mice. All graphs start from the age at which these behaviours were first displayed and do not depict earlier time points, as mice failed the tests at earlier stages.

(B-D) Time taken on each day for *Kat6b*$^{+/+}$ and *Kat6b*$^{+/+}$ mice to surface right (B), hold a bar (C) and rotate 180° when placed on an inclined surface (D).

N = 14 *Kat6b*$^{+/+}$ (6 males and 8 females, 6M/8F) and 9 *Kat6b*$^{+/–}$ (3M/6F) mice. Data presented as mean ± s.e.m. and were analysed using a Student's t-test (A) or two-way ANOVA with Holm-Sidak correction (B-D). Circles, triangles, individual female and male mice.

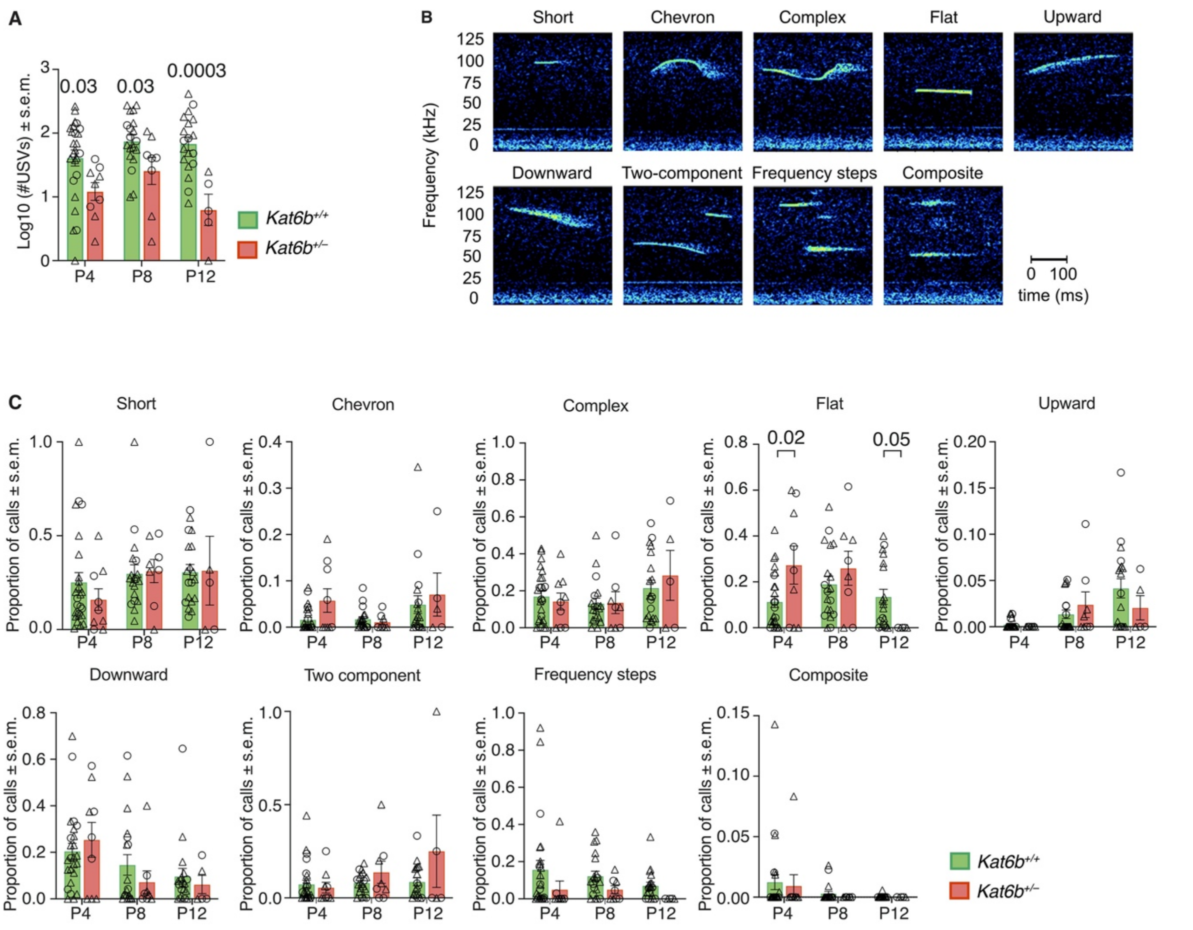

Supplementary Figure 8. Maternal separation induced ultrasonic vocalisations in *Kat6b*$^{+/+}$ and *Kat6b*$^{+/–}$ pups

(A) Number of ultrasonic vocalisations (USVs) recorded in a 3 min recording interval for *Kat6b*$^{+/–}$ and *Kat6b*$^{+/+}$ pups at postnatal days 4, 8 and 12, excluding pups that did not vocalise (for inclusive data see Figure 3F).

(B) Representative images of USV subtypes analysed.

(C) Proportion per USV call type in *Kat6b*$^{+/+}$ and *Kat6b*$^{+/–}$ mice at P4, 8 and 12, excluding mice that did not vocalise.

N = 24 *Kat6b*$^{+/+}$ (18M/6F) and 9 *Kat6b*$^{+/–}$ (5M/4F) (P4); 19 *Kat6b*$^{+/+}$ (14M/5F) and 8 *Kat6b*$^{+/–}$ (4M/4F) (P8); 19 *Kat6b*$^{+/+}$ (13M/6F) and 5 *Kat6b*$^{+/–}$ (2M/3F) (P12). Data are presented as

mean ± s.e.m. and were analysed using a two-way ANOVA with Holm-Sidak correction (A) or multiple t-tests (C). Circles, triangles, individual female and male mice.

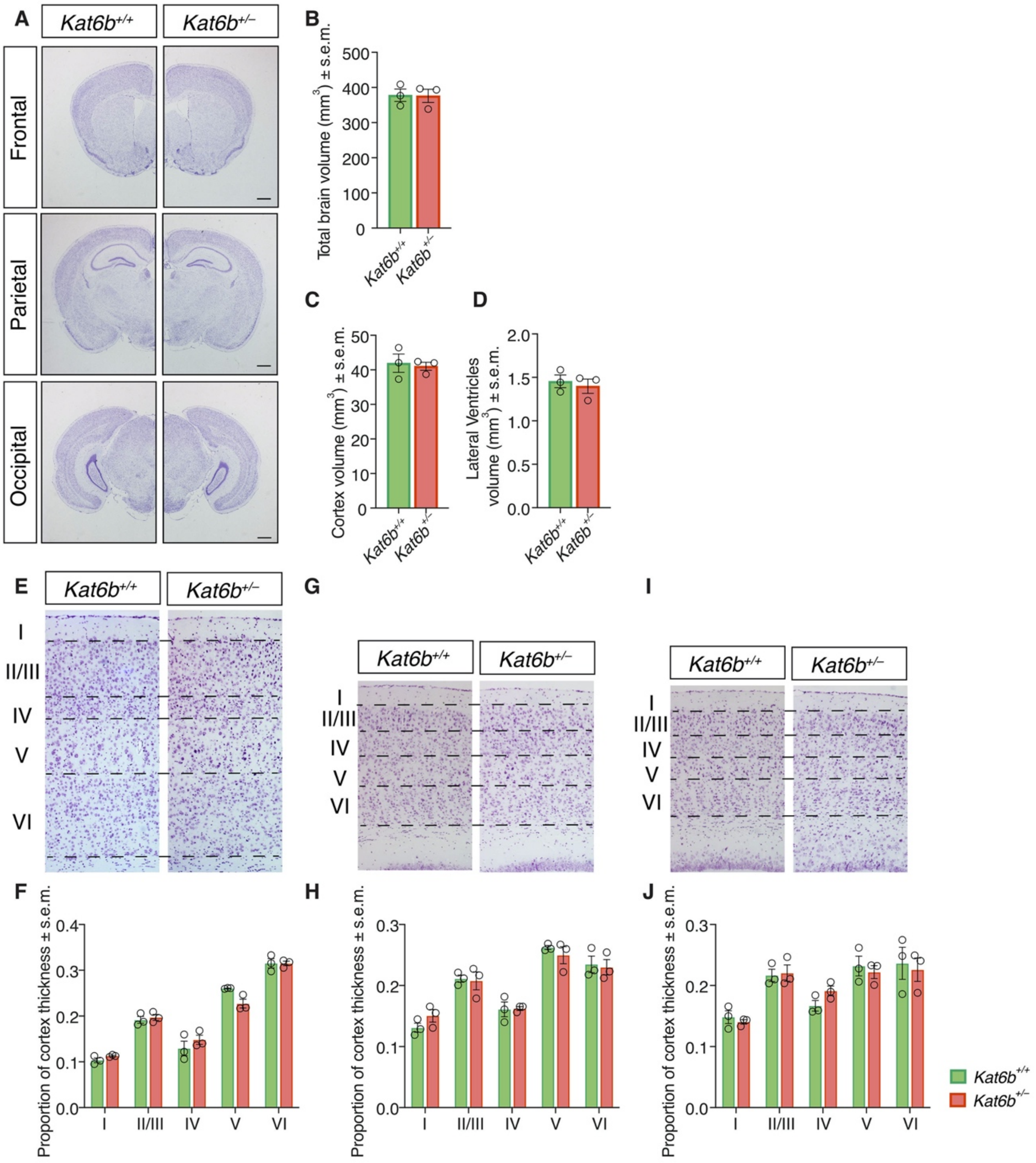

Supplemental Figure 9. Histological analysis of adult *Kat6b*$^{+/+}$ and *Kat6b*$^{+/–}$ mouse brains

(A,E,G,I) Representative images and morphometric analysis (B-D,F,H,J) of paraffin embedded brain sections of 12-week-old *Kat6b*$^{+/+}$ and *Kat6b*$^{+/–}$ mice stained with cresyl violet.

(A) Overview at the level of the frontal, parietal and occipital cortex. Scale bar = 500 µm.

(B) Total brain volume.

(C) Cerebral cortex volume.

(D) Lateral ventricle volume.

(E-J) Representative images (E,G,I) and proportion of each cortical layer of the total cortex depth (F,H,J) of the frontal cortex (E,F), parietal cortex (G,H) and occipital cortex (I,J). Dotted lines distinguish cortical layers I, II/III, IV, V and VI.

N = brains from 3 male 12-week-old mice per genotype. Data presented as mean ± s.e.m. and were analysed using a Student's t-test in (B-D) and two-way ANOVA with Holm-Sidak correction (F,H,J). Each circle represents an individual mouse.

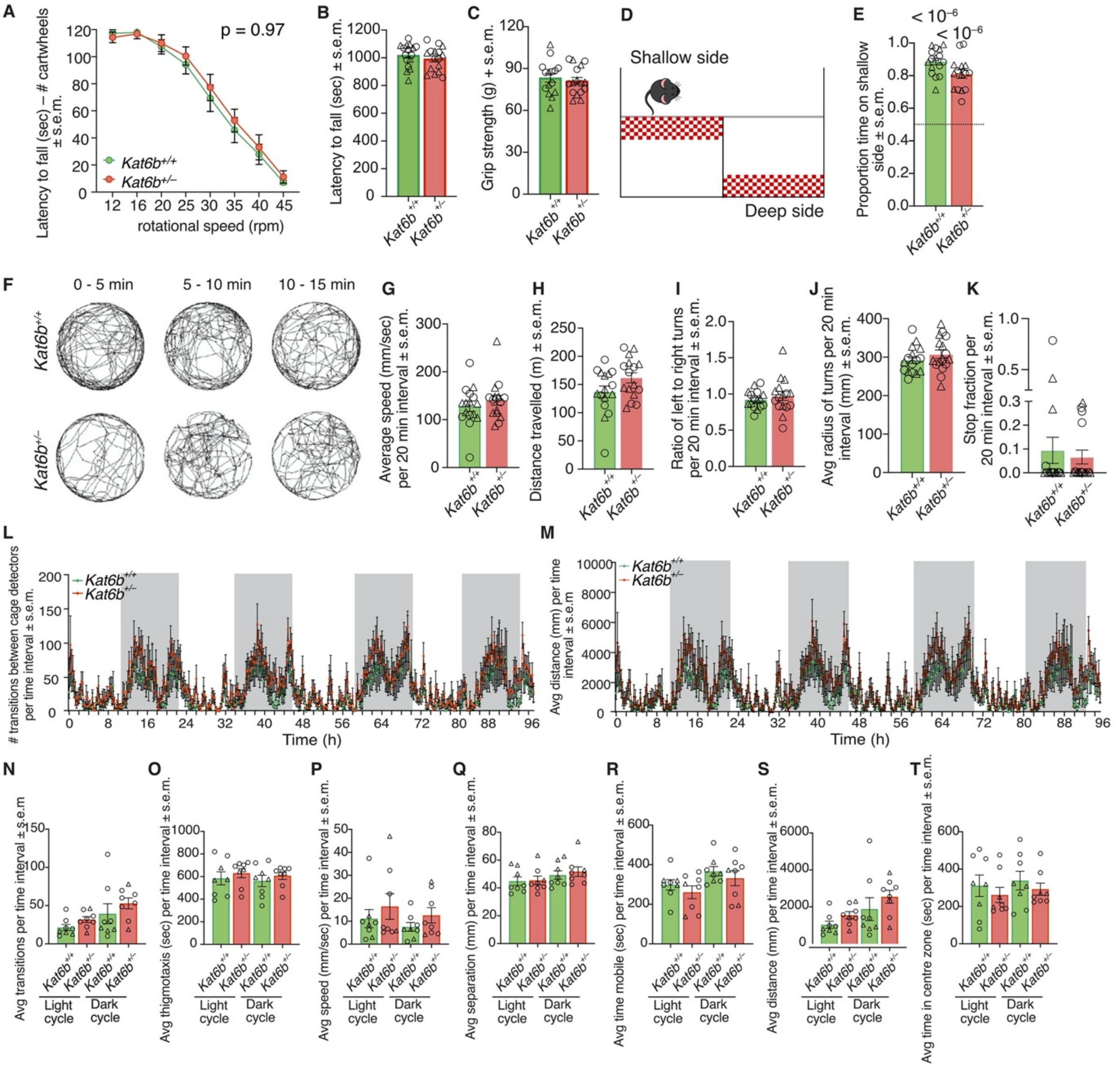

Supplemental Figure 10. Baseline analysis of adult *Kat6b*$^{+/+}$ and *Kat6b*$^{+/–}$ mice

(A) Rotor-rod test, latency to fall (sec) with 1 sec cartwheel penalty across increasing rotations per minute (rpm). The average of two trials performed on the same day is depicted.

(B) Latency to fall from the hanging mesh. The average of two trials performed on the same day is depicted.

(C) Grip strength. The average of the best 3 of 5 trials performed on the same day is depicted.

(D) Visual cliff indicating the shallow and deep sides. Grey line indicates clear acrylic plate that extends beyond the boundary between the shallow and deep sides.

(E) Proportion of time spent on the shallow side of the visual cliff compared to indiscriminate movement (0.5). Dotted line = 0.5.

(F) Representative traces in the open field test at 5 min intervals.

(G-K) Motor parameters per 20-min time interval in the open field test as indicated on the y-axes.

(L) Activity, defined as number of transitions between detectors per 15-minute time interval in the home cage across a 96-h testing period. Grey rectangles indicate the dark stages of the light/dark cycle.

(M) Average distance travelled per 15-minute time interval in the home cage across a 96-h testing period. Grey rectangles indicate the dark stages of the light/dark cycle.

(N-T) Movement parameters per 15-minute time interval, in the home cage analysis, in the light and dark cycles as indicated on the y-axes.

N = 16 *Kat6b*$^{+/+}$ (8M8F) and 16 *Kat6b*$^{+/–}$ (9M7F) mice (B,C,E,G-K); 8 *Kat6b*$^{+/+}$ (3M/5 F and 8 *Kat6b*$^{+/–}$ (3M/5F) mice (L-T). Data are presented as mean ± s.e.m. and were analysed using a Student's t-test (B,C,G-K), two-way ANOVA with Holm-Sidak correction (A,L,M,N-T) or one-sample t-test comparing to a theoretical value of 0.5 (E). Circles, triangles, individual female and male mice.

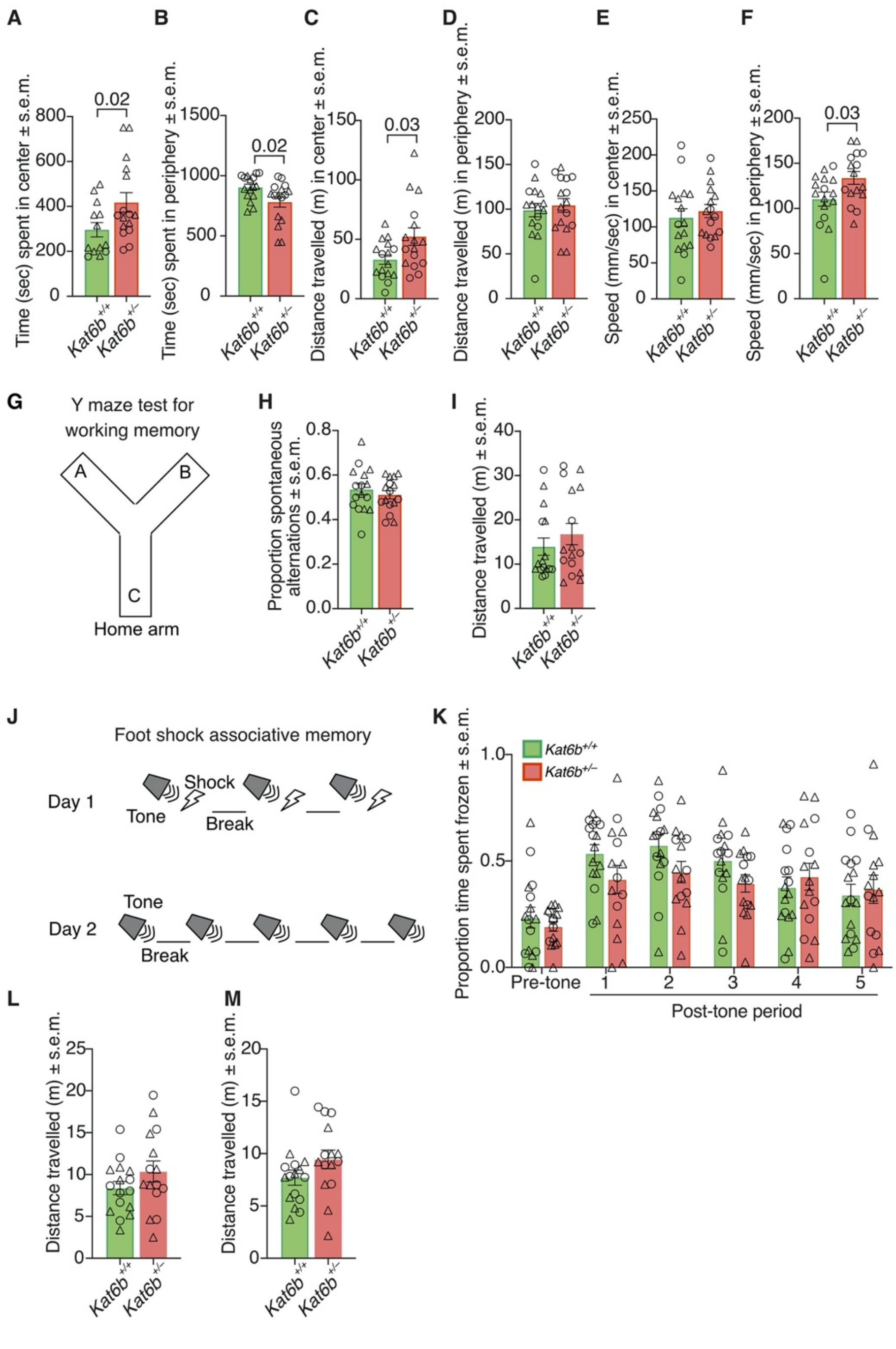

Supplemental Figure 11: Analysis of movements, spatial memory and associative learning in adult *Kat6b*$^{+/+}$ and *Kat6b*$^{+/–}$ mice

(A-F) Parameters time (A,B), distance travelled (C,D) and speed (E,F) of *Kat6b*$^{+/+}$ and *Kat6b*$^{+/–}$ mice in the centre (A,C,E) and periphery (B,D,F) of the open field arena.

(G) Depiction of the Y maze for working memory with arm A, B and C (home arm) indicated.

(H) Proportion of spontaneous alternations observed in *Kat6b*$^{+/+}$ and *Kat6b*$^{+/–}$ mice in the Y maze for working memory.

(I) Distance travelled in *Kat6b*$^{+/+}$ and *Kat6b*$^{+/–}$ mice in the Y maze for working memory.

(J) Depiction of the foot shock test for associative memory.

(K) Proportion of time spent frozen in the pre-tone and in each of 5 post-tone periods on day 2 of the foot shock test for associative memory.

(L, M) Distance travelled in session 1 (L) and session 2 (M) of the three-chamber social test.

N = 16 *Kat6b*$^{+/+}$ (8M/8F) and 16 *Kat6b*$^{+/–}$ (9M/7F). Data are displayed as mean ± s.e.m. and were analysed using an unpaired Student's t-test (A-F,H,I,L,M) or two-way ANOVA with Holm-Sidak correction (K). Circles, triangles, individual female and male mice.

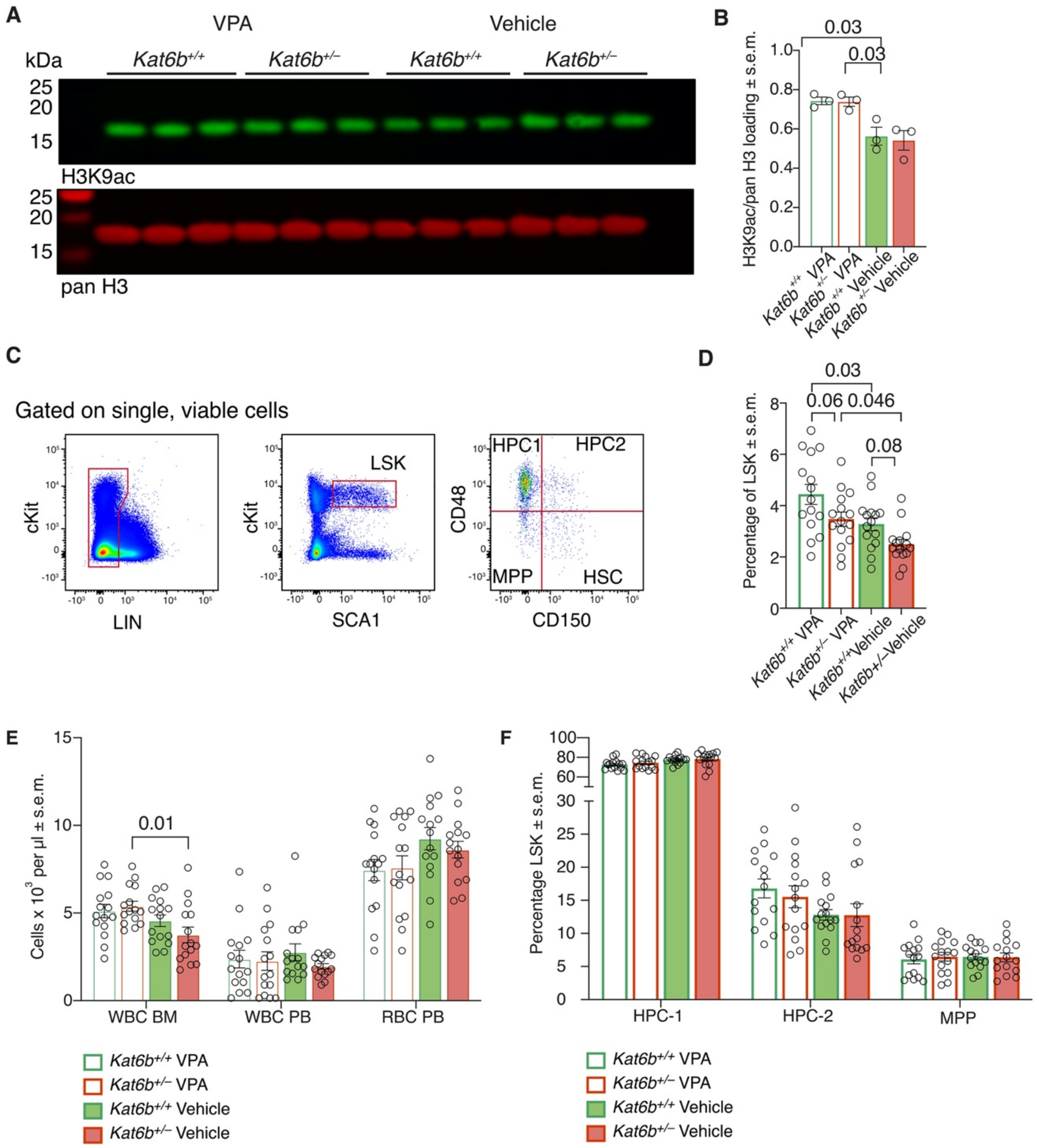

Supplemental Figure 12. Effects of VPA treatment of mice on histone acetylation in the brain and on haematopoietic cell numbers

(A,B) Representative Western immunoblot (A) and quantitation (B) of H3K9ac and pan H3 in the cortex of 19-day old *Kat6b*$^{+/+}$ and *Kat6b*$^{+/–}$ mice treated with VPA or Vehicle from 2 weeks of age for 5 days. Each lane represents an individual mouse. 0.5 µg of acid extracted protein loaded per lane.

(C) Representative flow cytometry plots showing gating for lineage (LIN) negative, SCA1 positive, KIT positive (LSK) cells, haematopoietic stem cells (HSCs, $CD150^{+}$, $CD48^{-}$) and progenitor cells (multipotent, MPP; haemopoietic progenitor cell 1 and 2, HPC1, HPC2) after size selection and gating on viable cells. Lineage (LIN) markers were B220, CD19, CD4, CD8, TER119, GR1 and MAC1.

(D) HSCs within the LSK population for VPA and Vehicle treated *Kat6b*$^{+/+}$ and *Kat6b*$^{+/-}$ mice assessed by flow cytometry.

(E) Number of white blood cells (WBC) per µl in peripheral blood (PB) and bone marrow (BM), as well as peripheral blood red blood cells (RBC) in VPA and Vehicle treated mice.

(F) Proportion of HPC-1, HPC-2 and MPP progenitor cell populations within the LSK population for VPA and Vehicle treated *Kat6b*$^{+/+}$ and *Kat6b*$^{+/-}$ mice.

N = 3 mice per genotype and treatment group (B); 15-16 *Kat6b*$^{+/+}$ (7-8M/7-8F) and 15 *Kat6b*$^{+/-}$ (7-8M/7-8F) per treatment group (C-F). Data are presented as mean ± s.e.m. and were analysed using a two-way ANOVA (B,D,E) or three-way ANOVA (F) with Holm-Sidak correction. Circles, individual mice.

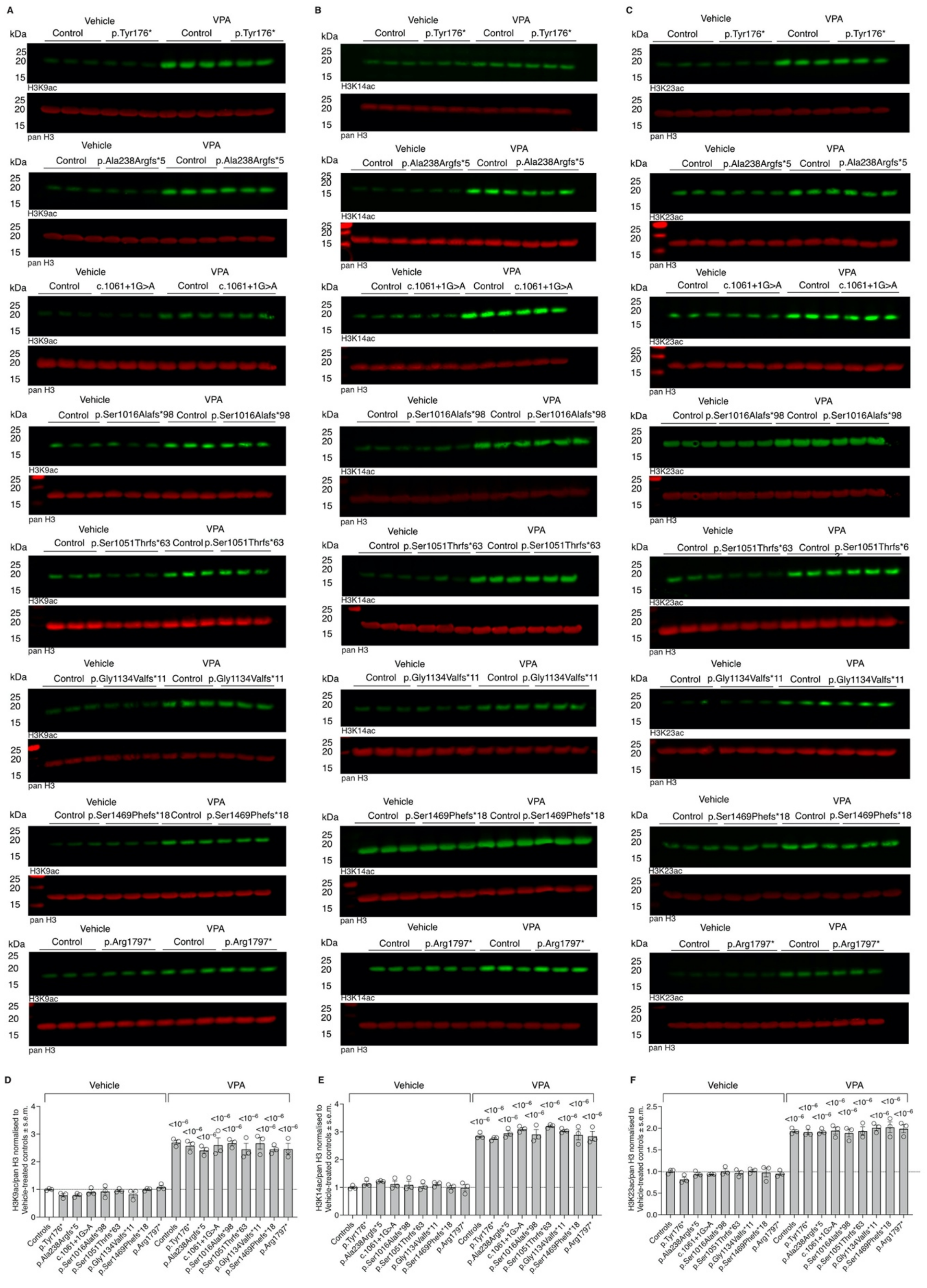

Supplemental Figure 13. VPA elevates histone acetylation in SBBYSS HEK293T cells

(A-C) Western immunoblots of H3K9ac (A), H3K14ac (B) or H3K23ac (C) and pan H3 in control HEK293T cells and cells carrying SBBYSS mutations treated with Vehicle or 1 mM

VPA. 0.5 μg (A), 2 μg (B) or 0.25 μg (C) protein loaded per sample. Each lane represents an individual cell clone.

(D-F) Quantitation of Western blot signals in (A-C) normalised to pan H3 loading and to Vehicle controls on the same blot. Each circle represents one lane of the corresponding western immunoblot.

N = 3 clones per mutation. Data are presented as mean ± s.e.m. and were analysed using a two-way ANOVA with Holm-Sidak correction.

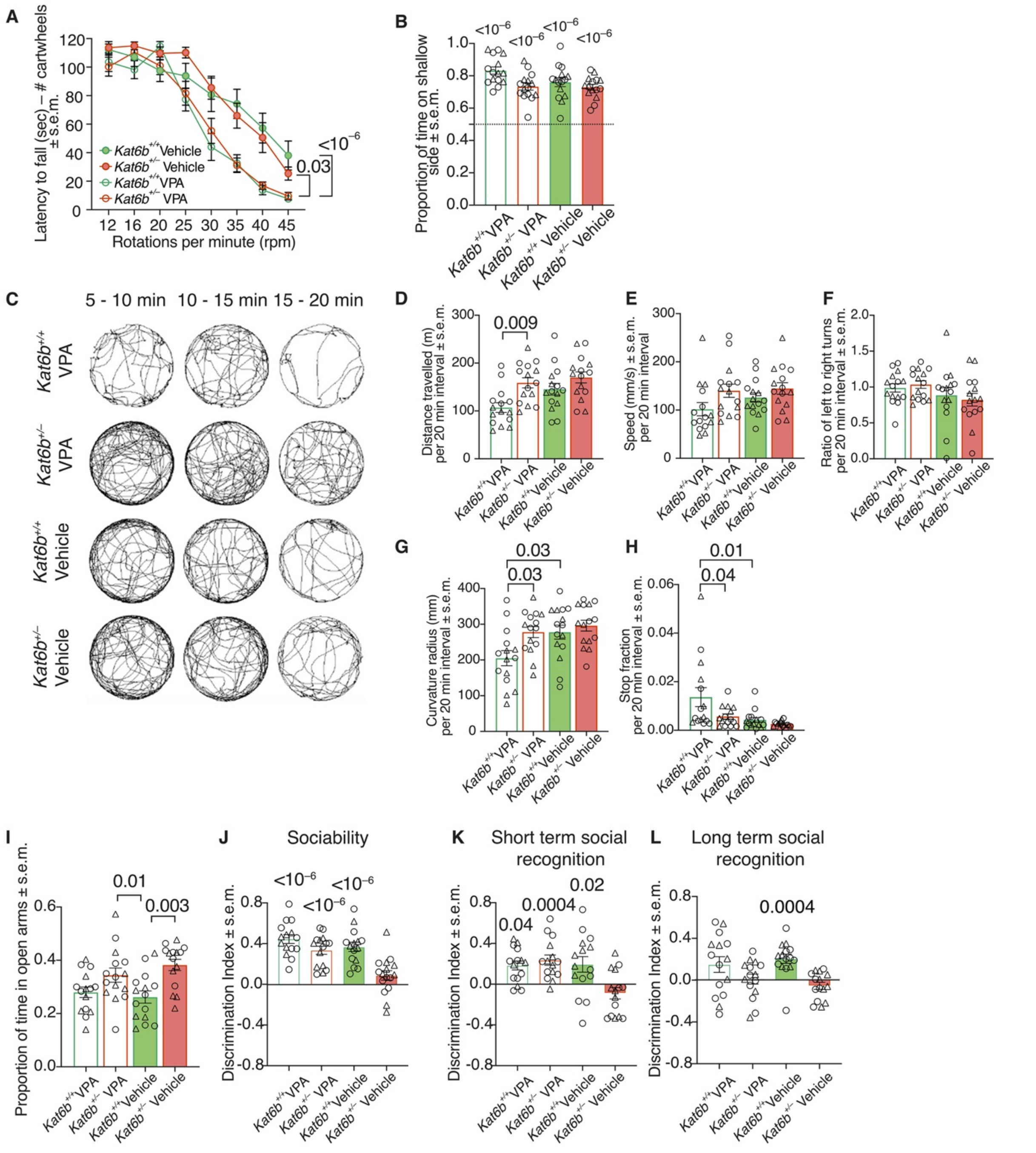

Supplemental Figure 14. Effects of VPA treatment on the behaviour of *Kat6b*$^{+/–}$ mice

(A-L) Behavioural test results of adult *Kat6b*$^{+/+}$ and *Kat6b*$^{+/–}$ mice treated with VPA or Vehicle from 2 weeks of age.

(A) Latency to fall (sec) with a 1 sec cartwheel penalty in the rotor-rod test. The average of two trials per mouse is depicted. There was no difference in the latency to fall between *Kat6b*$^{+/+}$

and *Kat6b*$^{+/–}$ Vehicle ($p = 0.39$) or VPA ($p = 0.18$) treated groups, but a significant effect of VPA treatment ($p = 0.03$ to $<10^{-6}$).

(B) Proportion of time spent on the shallow side of the visual cliff test compared to indiscriminate movement (0.5). Dotted line = 0.5.

(C) Representative traces of movement in the open field test. Traces are broken up into 5 min intervals.

(D-H) Motor parameters per 20-min time interval assessed in the open field test as indicated on the y-axes.

(I) Proportion of time spent in the open arms of the elevated O maze.

(J-L) Discrimination index for sessions 1, 2 and 3, respectively, of the three-chamber social test.

N = 15 *Kat6b*$^{+/+}$ (7-8M/7-8F) and 15 *Kat6b*$^{+/–}$ (7-8M/7-8F) per treatment group. Data presented as mean ± s.e.m. and were analysed using a three-way ANOVA with Holm-Sidak correction (A), one-sample t-test comparing to a theoretical value of 0.5 (B) or 0 (J-L) or a two-way ANOVA with Holm-Sidak correction (D-I). Circles, triangles, individual female and male mice.

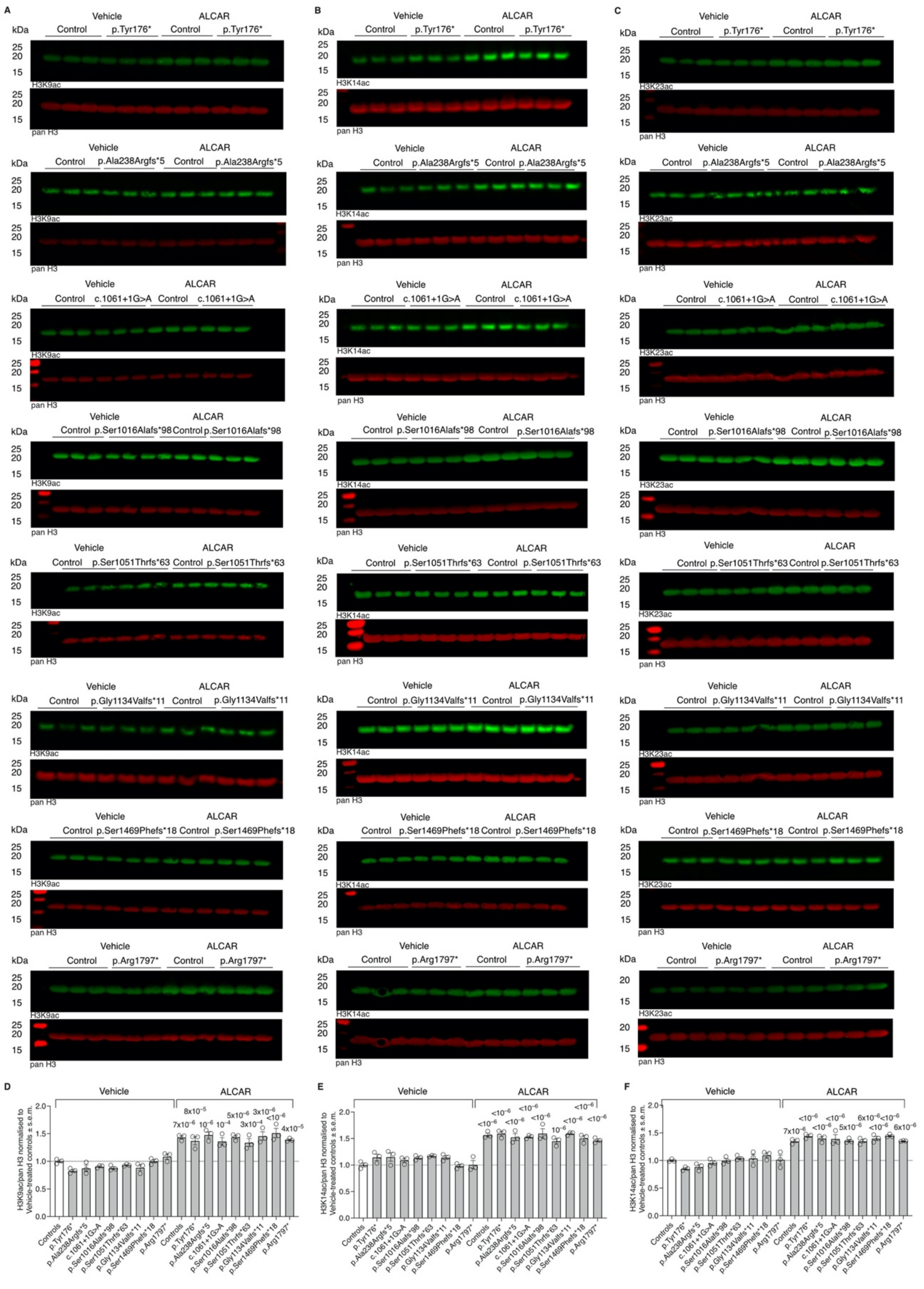

Supplemental Figure 15. ALCAR elevates histone acetylation in SBBYSS HEK293T cells

(A-C) Western immunoblots of H3K9ac (A), H3K14ac (B) or H3K23ac (C) and pan H3 in control HEK293T cells and cells carrying SBBYSS mutations treated with Vehicle or 1 mM

ALCAR. 0.5 µg (A), 2 µg (B) or 0.25 µg (C) protein loaded per sample. Each lane represents an individual cell clone.

(D-F) Quantitation of Western blot signals in (A-C) normalised to pan H3 loading and to Vehicle controls on the same blot. Each circle represents one lane of the corresponding western immunoblot.

N = 3 clones per mutation. Data are presented as mean ± s.e.m. and were analysed using a two-way ANOVA with Holm-Sidak correction.

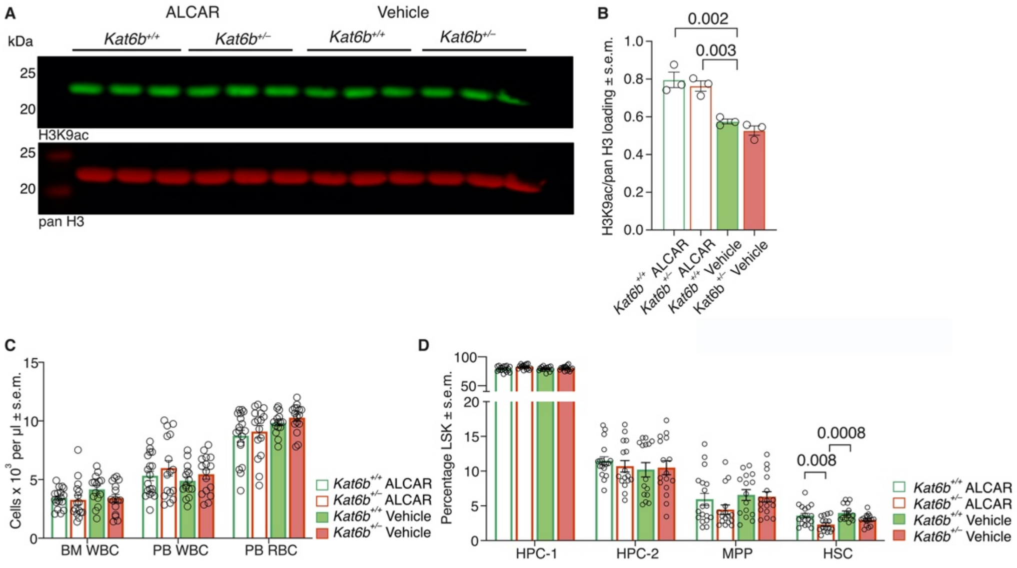

Supplemental Figure 16. Effects of ALCAR treatment of mice on histone acetylation in the brain and on haematopoietic cell numbers

(A,B) Representative Western immunoblot (A) and quantitation (B) of H3K9ac and pan H3 in the cortex of 19-day old *Kat6b*$^{+/+}$ and *Kat6b*$^{+/–}$ mice treated with ALCAR or Vehicle from 2 weeks of age for 5 days. Each lane represents an individual mouse. 0.5 µg of acid extracted protein loaded per lane.

(C) Number of white blood cells (WBC) per µl in peripheral blood (PB) and bone marrow (BM), as well as peripheral blood red blood cells (RBC) in ALCAR and Vehicle treated mice.

(D) Proportion of HPC-1, HPC-2, MPP and HSC populations within the LSK population for ALCAR and Vehicle treated *Kat6b*$^{+/+}$ and *Kat6b*$^{+/–}$ mice.

N = 3 mice per genotype and treatment group (B); 15-18 *Kat6b*$^{+/+}$ (7-8M/8-10F) and 16 *Kat6b*$^{+/–}$ (8-9M/5-7F) mice per treatment group (C,D). Each circle represents an individual mouse. Data are presented as mean ± s.e.m. and were analysed using a two-way ANOVA (B, C) or three-way ANOVA (D) with Holm-Sidak correction.

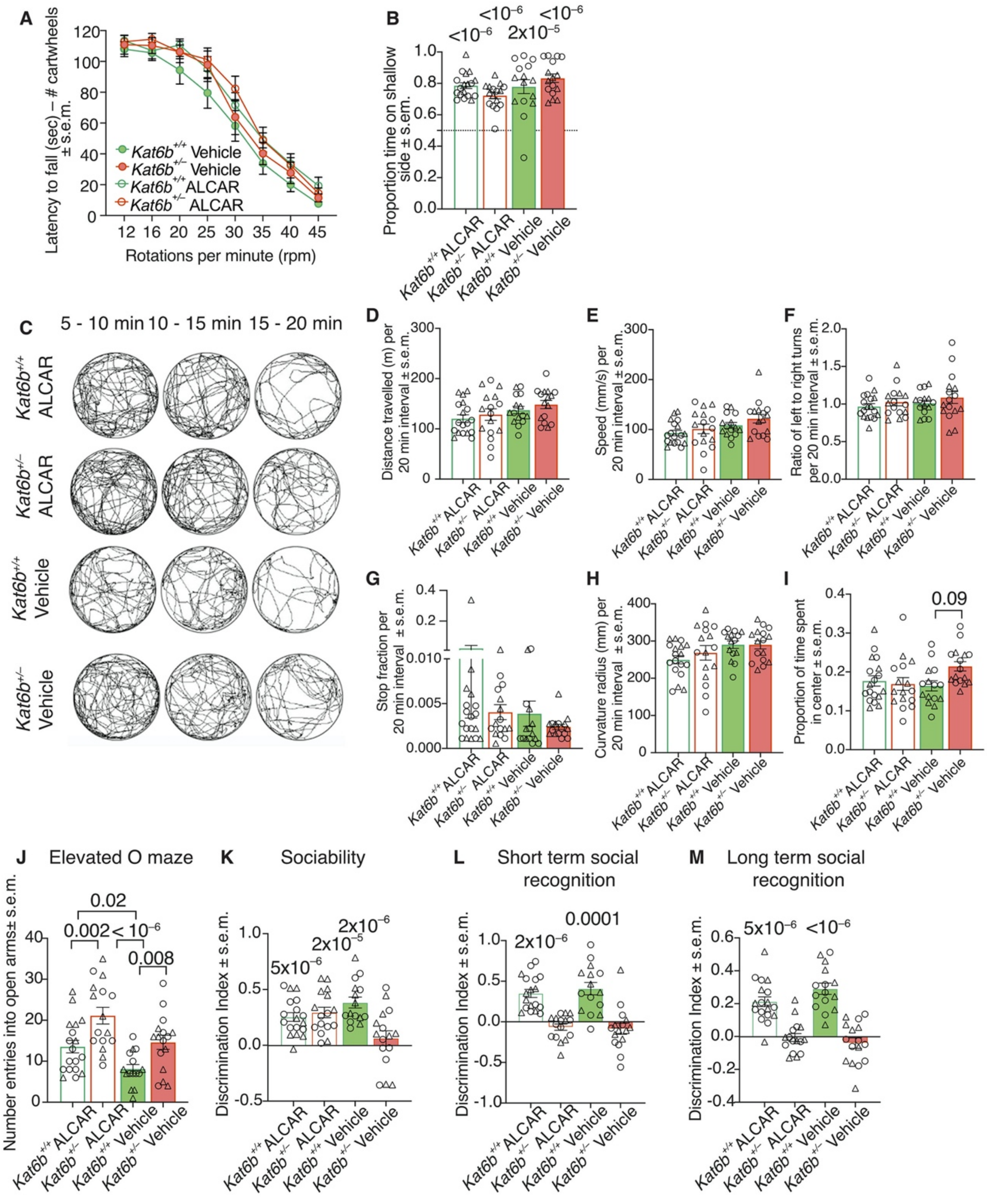

Supplemental Figure 17. Effects of ALCAR treatment on the behaviour of *Kat6b*$^{+/–}$ mice

(A-M) Behavioural test results of adult *Kat6b*$^{+/+}$ and *Kat6b*$^{+/–}$ mice treated with ALCAR or Vehicle from 2 weeks of age.

(A) Latency to fall (sec) with a 1 sec cartwheel penalty in the rotor-rod test. The average of two trials per mouse is depicted. No difference was observed between genotypes or treatment groups.

(B) Proportion of time spent on the shallow side of the visual cliff apparatus compared to indiscriminate movement (0.5). Dotted line = 0.5.

(C) Representative traces of movement within the open field test for ALCAR and Vehicle treated *Kat6b*$^{+/+}$ and *Kat6b*$^{+/–}$ mice. Traces are broken up into 5 min intervals.

(D-H) Motor parameters per 20-min time interval in the open field test as indicated on the y-axes.

(I) Proportion of time spent in the centre of the open field arena.

(J) Total number of entries into the open arms of the elevated O maze.

(K-M) Discrimination index for sessions 1, 2 and 3 of the three-chamber social test.

N = 15-18 *Kat6b*$^{+/+}$ (7-8M/8-10F) and 16 *Kat6b*$^{+/–}$ (8-9M/5-7F) mice per treatment group. Data presented as mean ± s.e.m. and were analysed using a three-way ANOVA with Holm-Sidak correction (A), one sample t-test comparing to a theoretical value of 0.5 (B) or 0 (K-M) or a two-way ANOVA with Holm-Sidak correction (D-I). Circles, triangles, individual female and male mice.

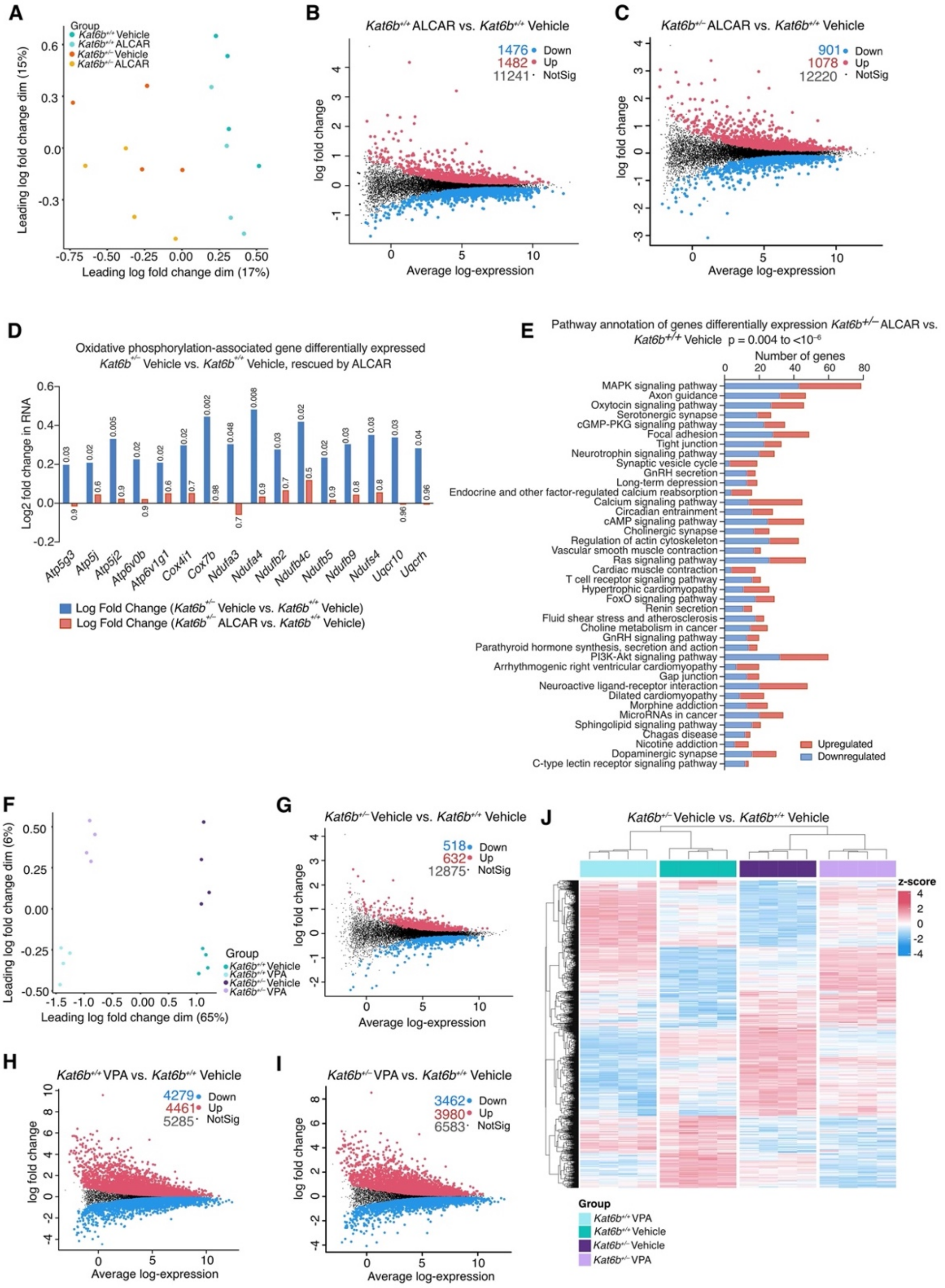

Supplemental Figure 18. Effects of ALCAR and VPA on gene expression in *Kat6b*$^{+/–}$ vs. *Kat6b*$^{+/+}$ E16.5 cortical neurons treated during ex vivo culture

(A-E) RNA sequencing results of cortical neurons isolated from *Kat6b*$^{+/–}$ vs. *Kat6b*$^{+/+}$ E16.5 mouse foetuses and cultured with 1 mM ALCAR, 1 mM VPA or Vehicle control (untreated medium) for 4 days. N = 4 foetuses (2M/2F) per genotype. An FDR < 0.05 was used as the significance cut-off. Data were analysed as described in the methods section.

(A) Multidimensional scaling (MDS) plot of *Kat6b*$^{+/–}$ vs. *Kat6b*$^{+/+}$ E16.5 cortical neurons treated with ALCAR vs. Vehicle control treatment.

(B) Mean-difference plot of expressed genes comparing ALCAR treated *Kat6b*$^{+/+}$ vs. Vehicle treated *Kat6b*$^{+/+}$ neurons, upregulated (red), downregulated (blue), not significantly changed (black).

(C) Mean-difference plot of expressed genes comparing ALCAR treated *Kat6b*$^{+/–}$ vs. Vehicle treated *Kat6b*$^{+/+}$ neurons.

(D) Oxidative phosphorylation (KEGG annotation) genes differentially expressed in Vehicle treated *Kat6b*$^{+/–}$ vs. Vehicle treated *Kat6b*$^{+/+}$ neurons (FDR = 0.048 to 0.003; blue), but not in ALCAR treated *Kat6b*$^{+/–}$ vs. Vehicle treated *Kat6b*$^{+/+}$ neurons (p = 0.1 to 1.0; red).

(E) KEGG pathway annotations of genes differentially expressed in Vehicle treated *Kat6b*$^{+/–}$ vs. Vehicle treated *Kat6b*$^{+/+}$ neurons and not rescued in ALCAR treated *Kat6b*$^{+/–}$ vs. Vehicle treated *Kat6b*$^{+/+}$ neurons. Number of affected genes in each pathway, downregulated genes (blue), upregulated genes (red).

(F) MDS plot of *Kat6b*$^{+/–}$ vs. *Kat6b*$^{+/+}$ E16.5 cortical neurons treated with VPA vs. Vehicle.

(G-I) Mean-difference plot of expressed genes comparing Vehicle treated *Kat6b*$^{+/–}$ vs. Vehicle treated *Kat6b*$^{+/+}$ neurons (G), comparing VPA treated *Kat6b*$^{+/+}$ vs. Vehicle treated *Kat6b*$^{+/+}$ neurons (H), or comparing VPA treated *Kat6b*$^{+/–}$ vs. Vehicle treated *Kat6b*$^{+/+}$ neurons (I).

(J) Heatmap of genes differentially expressed between Vehicle treated *Kat6b*$^{+/–}$ vs. Vehicle treated *Kat6b*$^{+/+}$ neurons (in the VPA vs. Vehicle experiment). Results for VPA treated *Kat6b*$^{+/–}$ and VPA treated *Kat6b*$^{+/+}$ neurons are also shown.

Supplemental Tables

Supplemental Table 1: CRISPR/Cas9 crRNA, HDR and MiSeq primer sequences

Protein sequence mutation	*Coding sequence mutation*	*Reference*	*crRNA sequence*	*ssHDR sequence 5' –> 3'*	*MiSeq Fdw primer with Overhang sequence 5' –> 3'*	*MiSeq Rev primer with Overhang sequence 5' –> 3'*
Eco R1 Control	N/A	N/A	CCTCTAA<br>GGTTTGC<br>TTACGA	AGCCATCTCTCTCCTTGC<br>CAGAACCTCTAAGGTTTG<br>CTTAGAATTCCGATGGAG<br>CCAGAGAGGATCCTGGG<br>AGGGAGAGCTTGGCA	GTGACCTATGAA<br>CTCAGGAGTCCA<br>GCTCAGGTTCTG<br>GGAGAG	CTGAGACTTGCA<br>CATCGCAGCCAT<br>CCTTAGGCCTCC<br>TCCTT
*p.Tyr176**	c.527dup	Clayton-Smith et al., 2011	CGCAGTA<br>CAGGGTC<br>AATTAT	TGGGAGGTTACTGAAAG<br>ACGGACCGCAGTACAGG<br>GTCAATTAATGGGAGCTT<br>AGATGGCAAAGGGGCAC<br>CTCAGTATCCCAGT	GTGACCTATGAA<br>CTCAGGAGTCAA<br>GGACTTGAGGA<br>GCCGAAT	CTGAGACTTGCA<br>CATCGCAGCTCA<br>TGGGGTAGAAG<br>GCTGAC
*p.Ala238Argfs*5*	c.708-709delTT	Zhang et al., 2020	TGCCACA<br>ATCTGCA<br>CAAGAG	TAGGAAAAACACAACTT<br>ACCACTACTGCCACAATC<br>TGCACGAGAGGAGTTCTT<br>CTGGTTTCTTTTCACGAT<br>TTGATTCTT	GTGACCTATGAA<br>CTCAGGAGTCGT<br>GCTGATCCCATT<br>CCAATA	CTGAGACTTGCA<br>CATCGCAGCTCA<br>GTACTAACATAC<br>TCACGCTTTTT
	c.1061+1G>A	Zhang et al., 2020	ATTAAAG<br>CAACGAT<br>TGTTGT	CCCATTGGACGACCGAA<br>AAATAAATTAAAGCAAC<br>GATTGTTATAGCTTGAGA<br>TCTTATCAAAAGAAATCA<br>TTTATGTTTTGC	GTGACCTATGAA<br>CTCAGGAGTCAT<br>TCAGGGATGTGG<br>ATTTGC	CTGAGACTTGCA<br>CATCGCAGCTTC<br>AACCCTTGAGTT<br>TGAAAAA
*p.Ser1016Alafs*98*	c.3046del	Zhang et al., 2020	TAATGGA<br>ACAAGCT<br>AGCTGC	TACCCTCCCCACTTAGGC<br>TGAGCGGCTAATGGAAC<br>AAGCTGCTGCTGGGAGA<br>AGGAGGAACAAGAAATC<br>CTGTCAACTAGAG	GTGACCTATGAA<br>CTCAGGAGTCCA<br>CTTTGCCATTGA<br>TCCTCA	CTGAGACTTGCA<br>CATCGCAGCCTG<br>CTCTCTTTAGAC<br>AGCTCCA
*p.Ser1051Thrfs*63*	c.3152delG	Brea-Fernandez et al., 2019	TGTGACT<br>GGCCGGC<br>TCTCCG	CAGCTCCAGCAGCTGCCC<br>TCGCTCCCCTGTGACTGG<br>CCGGTCTCCGGGGAATGC<br>AAATATTTATTTTTCGAT<br>TGTACTTTTGC	GTGACCTATGAA<br>CTCAGGAGTCCA<br>GTAGGCAATCAC<br>CTGCAA	CTGAGACTTGCA<br>CATCGCAGCTGG<br>CAACTGACTGTG<br>GTTTC
*p.Gly1134Valfs*11*	c.3401delG	Zhang et al., 2020	AGAAAA<br>GGGGTCG<br>TAAACGC	AATTAATTTCAGAGGCCT<br>TTTGTACTAAAGAAGAA<br>AAGGGTCGTAAACGCAG<br>GAGGATCAACAGCAGTG<br>TAACAACAGAGACCATTT	GTGACCTATGAA<br>CTCAGGAGTCTC<br>AGACACGCGTTC<br>AGTACA	CTGAGACTTGCA<br>CATCGCAGCGCC<br>ATCTTCCTCCAC<br>TTCAA
*p.Ser1469Phefs*18*	c.4405dupT	Clayton-Smith et al., 2011	ACCTCTG<br>GGTTCGA<br>GTGACC	GTCAGGTCGACGCCACA<br>GTCCATTAAGACCTCTGG<br>GTTCGAAGTGACCAGGCT<br>GCACATTAAGGTCTAAA<br>AAAGTCTCCTGGTTT	GTGACCTATGAA<br>CTCAGGAGTCAT<br>GAAGAGCCATC<br>CCACAAC	CTGAGACTTGCA<br>CATCGCAGCCTC<br>CTCGTCAGATTC<br>GGGTA
*p.Arg1797**	c.5389C > T	Clayton-Smith et al., 2011	CATTGGC<br>TTATACG<br>AGCGAA	AATCCCCGAGACGAGCA<br>ACGCCAACATTGGCTTAT<br>ACGAATGAATGGGTCAG<br>AGTGATTTTGGGGCTGGG<br>CATTACCCGCA	GTGACCTATGAA<br>CTCAGGAGTCTC<br>TCCTCAAGGCTG<br>TGTGGT	CTGAGACTTGCA<br>CATCGCAGCTGG<br>CTGTAAGGCAAT<br>GAATG

Supplemental Table 2: Miseq validation of SBBYSS mutations in HEK293T cells provided as Excel file

Supplemental Table 3: Observations of *Kat6b* mutant and control mice following caesarean section at E19.25

	Time to inflate lungs (min)	Time to pink skin (min)*	Time to establish regular breathing (min)	Milk in stomach 1-2 h after birth
$Kat6b^{+/+}$	2.35 ± 0.38	4.34 ± 0.39	5.55 ± 0.53	Yes
$Kat6b^{+/–}$	3.36 ± 0.55	5.64 ± 0.51	7.82 ± 1.3	Yes
$Kat6b^{–/–}$	8.44 ± 1.48	11.25 ± 1.40	34.19 ± 5.75	N/A
p value, $Kat6b^{–/–}$ *vs.* $Kat6b^{+/+}$	0.0002	0.00004	0.000008	

*, indication of blood oxygenation. N = 4-7 mice per genotype. Data analysed using a one-way ANOVA with Benjamini and Hochberg correction for multiple testing.

Supplemental Tables 4 to 7 reporting RNA sequencing results of cortical neurons derived from E16.5 $Kat6b^{+/–}$ and $Kat6b^{+/–}$ foetuses treated with Vehicle, VPA or ALCAR including all genes and differentially expressed genes and KEGG pathway enrichment are provided as four Excel files

Supplemental Table 8: Genotyping primers

Targeted sequence	Primer sequence 5' –> 3'		PCR amplicons
Kat6b+<br>*Kat6b –*	F1<br>F2<br>R	TGCCTCAGAAAGCCATTACC<br>CAGACAAATCAGCCCCAGAT<br>GAGGCTCAGGGCTACAAGTG	WT = 230 bp<br>Del = 600 bp

Supplemental Table 9: RT-qPCR primers

Targeted sequence	Primer sequence 5' –> 3'	Reference
KAT6B (human)	F GGATTTGGACGGTTTCTCATTG<br>R GAGATACTCCAAGATGACGCTC	This study
GAPDH (human)	F TGCACCACCAACTGCTTAGC<br>R GGCATGGACTGTGGTCATGAG	Wichmann et al., 2022 (1)
Kat6b (mouse)	F GTGCTTTTCCGTCCTCACTCC<br>R CACGATTTGACTCTTTAGTCCCC	This study
Gapdh (mouse)	F TGCACCACCAACTGCTTAGC<br>R GGCATGGACTGTGGTCATGAG	Wichmann et al., 2022 (1)

Supplemental Table 10: Buffers and solutions

Buffer	*Recipe*
2% FACS buffer pH 7.1 - 7.2	2% (vol/vol) FCS, 150 mM NaCl, 3.7 mM KCl, 2.5 mM $CaCl_2.2H_2O$, 1.2 mM $MgSO_4.7H_2O$, 0.8 mM K_2HPO_4, 1.2 mM KH_2PO_4, 11.5 mM HEPES, made up in MQ-H_2O
PBS	16 mM $Na_2HPO_42•H_2O$, 0.4 mM $NaH_2PO_4•H_2O$, 150 mM NaCl, in MQ-H_2O, pH 7.4
Red cell lysis buffer pH 7.2	150mM ammonium chloride (NH4Cl), 0.1 mM EDTA, 12 mM $NaHCO_3$ made up in MQ-H_2O
Histone acid extraction buffer	10mM HEPES (pH 7.9), 1.5 mM $MgCl_2$, 10 mM KCL, 0.5 mM DTT, 1M $Na(C_3H_7COO)$ and 100 mM EDTA

Supplemental Table 11: Antibodies

Marker	*Fluorophore*	*Clone number*	*Order ref*
LIN (B220, CD19, CD4, CD8, GR1, TER119, LYG6)	A700	B220 clone 16A, CD19 clone 1D3, CD4 clone 17A2, CD8a clone 53-6.7, Gr1 clone RB6-8C5, Ter119 clone TER-119 and LyG6	Made in house
cKIT	PerCP/Cy5.5	BD clone 2B8	#563160
SCA1	A594	Clone E13	Made in house
CD48	PECy7	eBiosciences clone HM48-1	#25-0481-80
CD150	A647	BioLegend clone TC15-12F12.2	#115918
B220	A700	B220 Clone 16A	Made in house
CD19	PECy7	BD Pharmingen clone 1D3	#552854
CD4	APC	GK1.5	BD 553730
CD8	PE	Clone 56.3.7	Made in house
GR1	A594	Clone 1A8	Made in house
MAC1/LYG6	PerCP/CY5.5	BioLegend clone ICRF44	#301327
H3K9ac	Unconjugated	C5B11	Cell signalling #9649
H3K9ac	Unconjugated	13-0033	Epicypher
H3K14ac	Unconjugated	Ab52946	Abcam
H3K23ac	Unconjugated	07-335	Millipore
Pan H3	Unconjugated	Sp2/0-Ag14	Abcam, ab10779
LIVE/DEAD Fixable dead cell stain	A488	N/A	ThermoFisher, L23101
Viability marker	Flurogold	Polyclonal	AB153-I
Anti-rabbit Secondary for FACS	Pacific Blue	Polyclonal	Jackson Immunoresearch, 111-475-003
Anti-rabbit secondary for western immunoblotting	IRDye® 680	Polyclonal	Li-Cor, 926-68071
Anti-Mouse secondary for western immunoblotting	IRDye® 800	Polyclonal	Li-Cor, 926-32210

Supplemental Methods

CRISPR/Cas9 HDR of SBBYSS mutations in HEK293T cells

SBBYSS-causing mutations were generated in HEK293T cells using CRISPR/Cas9 and homology direct repair (HDR), as described (2). The Alt-R CRISPR HDR Design Tool (IDT https://sg.idtdna.com/pages/tools/alt-r-crispr-hdr-design-tool) was used to design custom guide RNAs and single-stranded HDR donor DNA oligonucleotides to generate 8 *KAT6B* SBBYSS mutations, as well as positive control cells that retained the wild-type sequence but carried a silent mutation including a novel EcoR1 restriction enzyme cut site in *KAT6B* (Supplemental Table 1). A ribonucleoprotein (RNP) approach with Lipofectamine CRISPRMAX Cas9 Transfection Reagent (ThermoFisher Scientific, CMAX00015) transfection was performed, following a modified Alt-R CRISPR-Cas9 System protocol (IDT). Briefly, custom crRNAs were complexed with trRNAs to form 1 µM gRNAs. Per HDR reaction, 3.75 µl of 1 µM gRNA, 0.125 µl of 5 µg/µl TrueCut Cas9 v2 (ThermoFisher Scientific, A36498) and CRISPRMAX Cas9 Plus Reagent (ThermoFisher Scientific, CMAX00015) were combined with 57.125 µl Gibco OptiMEM medium (ThermoFisher Scientific, 31985088). Following a 5 min incubation at room temperature, 3.75 µl of the appropriate 0.3 µM ssDNA repair template was added and incubated another 5 min. Next, 59.5 µl OptiMEM, 3 µl CRISPRMAX transfection reagent (ThermoFisher Scientific, CMAX00015) and 0.55 µl Alt-R HDR Enhancer v2 (IDT, 10007910) was added to the RNP complexes and incubated for 20 min at room temperature. HEK293T cells were diluted to 200,000 cells/ml in Gibco DMEM (ThermoFisher Scientific, 11885084) with 10% foetal bovine serum (FBS) and without antibiotics. In a 48-well plate, 250 µl diluted cells (50,000 cells/well) were combined with 125 µl of the transfection complex mixture and incubated for 24 hours before removing RNP medium and replacing with DMEM with 10% FBS and 100 Units/ml penicillin/streptomycin (15140122, ThermoFisher Scientific). Editing efficiency was determined on bulk-edited cell populations by targeted PCR of the

Cas9-targeted region and secondary PCR using overhang sequences (Supplemental Table 1), followed by Illumina MiSeq sequencing, as previously described (3). MiSeq sequencing results for the SBBYSS mutations generate are displayed in Supplemental Table 2 supplied as an Excel file. The intended SBBYSS mutation was highly represented 92-100% in sequence reads of the replicate clonal cell lines generated for 4 of the 8 SBBYSS mutations. Although the other 4 SBBYSS mutations ranged in sequence reads from 51-100% within their replicate clonal cell lines, the effects on histone acetylation were curiously consistent between clonal lines, possibly because the alternative CRISPR/Cas9 mutations were indels within the same sequence region.

Mice

Mice were housed in groups of 4-6 in ventilated cages (AirLaw) and provided with γ-irradiated feed (Barastoc, Ridley AgriProducts) and sterilised water. Mice were held in a 14-h light and 10-h dark cycle.

Kat6b null allele

The *Kat6b* locus was targeted twice (Supplemental Figure 5A-C). Embryonic stem cells (ESCs) were first targeted to flank *Kat6b* exon 2 with *loxP*-sites. *Kat6b*$^{\text{exon2-fl}}$ mice were generated and used to isolate blastocytes and embryonic stem cells that were then retargeted to flank *Kat6b* exons 11 to 12 with *loxP*-sites (*Kat6b*$^{e2fl\&e11\text{-}12\text{-}fl}$). *Kat6b*$^{e2\text{-}fl\&e11\text{-}12\text{-}fl}$ mice carrying the 4 *loxP* sites were crossed to a Cre-deleter strain (4), resulting in the Cre-recombinase-mediated deletion of exons 2-12 of the *Kat6b* gene creating a *Kat6b* null allele (*Kat6b*$^{-}$). *C57Bl/6 x 129Sv* hybrid ESCs were originally used to enable efficient re-targeting of the *C57Bl/6* allele, but mice were then backcrossed to *C57Bl/6* mice for more than 10 generations. Mice were genotyped by PCR using primers displayed in Supplemental Table 8.

Behavioural tests

Light levels were standardised across tests using a luminometer (Lutron LM-81LX. S041136) and the temperature maintained at 22°C. Testing was recorded from above using an HD WedCam C615 (Logitech; Lausanne, Switzerland), excepting the home cage observation, which was recorded from the side (Actual Analytics). Behavioural assays were performed in the light phase of the light/dark cycle and only one test was performed per day. Prior to testing all mice were habituated to the operator. Mice were habituated to the testing room 30 min prior to testing. A low volume (~60 decibel) white noise speaker was turned on throughout testing. All mice were returned to their home cage following testing.

Ultrasonic vocalisations

Individual pups were separated from their mother and placed into a sound-attenuated chamber, pre-warmed to nesting temperature (~34°C). Mice were separated and recorded for 3 min on postnatal days 4, 8 and 12. Vocalisations were visualised and analysed using bioacoustics software (Avisoft SASLabPro).

Basic Behavioural milestones

Developmental milestones as described (5) were assessed in *Kat6b*$^{+/-}$ and *Kat6b*$^{+/+}$ mice from postnatal day 1 to 21.

Open Field test

The open field test was performed as described (5), with modifications as described (6). Each mouse was placed in an arena comprising a 90 cm diameter circular floor and 0.4 m high black corrugated wall surrounded by a white opaque curtain. Light intensity was 50 lux in the centre

of the arena. Mice were allowed to freely explore the arena for 20 min. Mouse ambulation was analysed using MouseMove software as described (6).

Novel object recognition

The novel object recognition test was performed as described (7, 8), with minor modifications as described (6). Object recognition was assessed by calculating the discrimination index (DI) (9), defined as

$$\text{Discrimination Index} = \frac{(\text{Time exploring the novel object} - \text{Time exploring the familiar object})}{(\text{Time exploring the novel object} + \text{Time exploring the familiar object})}$$

The discrimination indices of all genotype and treatment groups were compared using a one sample t test, to a theoretical value of 0, which would indicate no preference for the novel or familiar object or mouse.

Y maze for spatial and working memory

Two tests were carried out using the Y maze, as described (10). The Y maze comprised three arms at 120 degrees to each other. Each arm was 38 cm long, 7 cm wide and surrounded by 12 cm high opaque walls. Two test arms (A and B) contained guillotine doors at the stem to block entry if required, while a third arm (C) was designated the home arm and contained a 10 cm^2 start area.

In the working memory test mice were placed in the start area of the home arm and allowed to freely explore all three arms of the maze for 5 min. The sequence of entries into each arm was assessed and one *"spontaneous alternation"* was recorded when a mouse had entered each of

the three arms within three consecutive arm entries. The proportion of spontaneous alternations were defined as the number of alternations / (total number of arm entries – 2).

In the spatial reference memory test mice firstly underwent a training phase, where one arm, A or B, was blocked ("novel arm"). Mice were placed in the start area of the "home arm" and allowed to explore the available arms (home arm and "familiar arm") for 10 min. Mice were returned to their home cage for 1 h. The novel arm was unblocked, and mice allowed to explore all three arms of the maze for 5 min. The proportion of time spent in the novel arm relative to the familiar arm, excluding the home arm was assessed, as was the discrimination index for the novel versus familiar arm.

Elevated O maze

The elevated O maze test was performed as described (11). The apparatus comprised a 10 cm wide annular platform 60 cm above the floor, with two opposing, open sections and two opposing, enclosed sections surrounded by a 20 cm high opaque wall. Light intensity was set to 100 lux. Test mice were placed at the border between open and closed section with its head facing an open section and allowed to freely explore for 5 min. The time spent collectively within the open or closed sections was assessed, as was the number of entries into the open sections.

Grip Strength Test

The grip strength test was performed using a grip strength meter (BIO-GS3, Bioseb). Mice were held by the tail and gently lowered towards the meter until they grabbed the bar with both forepaws. Mice were gently pulled away from the bar and the maximum force used by the

mouse before releasing the bar was recorded. Mice were given 5 trials, separated by a 1 min rest. The best 3 of these trials was used to compare grip strength between genotypes.

Three-chamber social test

The three-chamber social test was performed as described (12). The apparatus consisted of a clear acrylic enclosure with two end chambers (21 cm H x 27 cm W), each containing a circular wire cage (10 cm diameter). The central chamber (21 cm H x 12 cm W) had a metal grill floor (aversive). Opaque, dark, vertically adjustable internal walls separated each end chamber from the central chamber. An adjustable space at the base of each internal wall allowed a test mouse to move from one section to another.

Mice were first habituated to the empty apparatus for 10 min before being returned to their home cage for 1 h. Mice were returned to the apparatus with one cage still empty and the other containing an age, sex and genetic background-matched mouse. The test mouse was placed into the central chamber and allowed to explore for 5 min. 1 h later the original control mouse (familiar mouse) was placed into one of the wire cages and a new age, sex and genetic background matched mouse (novel mouse) was placed into the wire cage in the second end chamber. The test mouse was placed into the central chamber and allowed to explore for 5 min. The following day the familiar mouse was placed into the wire cage in one of the end chambers and a new age, sex and genetic background matched novel mouse was placed in the other. The test mouse was placed in the central chamber and allowed to explore for 5 min. The proportion of time interacting with each cage, as indicated by nose pointing and sniffing around each wire cage, was assessed. Prior to testing all familiar and novel mice were habituated to the wire cage for 10 min per day for 3 days. The proportion of interaction time spent with the mouse versus

empty cage or novel versus familiar mouse was assessed and the discrimination index for the mouse versus empty cage and novel versus familiar mouse was calculated.

Foot shock test for associative memory

Fear conditioning with an auditory cue was performed as described (13). On day 1, test mice were placed into a sound attenuated fear conditioning chamber (Maze Engineers) and allowed to explore for 3 min. A 70 dB tone was played for 15 sec. A 0.4 mA, 1 sec foot shock coincided with the final second of the tone. A 60 sec break was given before the second shock and this pattern repeated once more for a total of 3 tone/shock pairings. On day 2, mice re-entered the chamber and were allowed to explore for 3 min. A 70 dB 15 sec tone was played without a shock, followed by a 60 sec break. A total of 5 unpaired tones were played. The proportion of time spent frozen, defined as an absence of movement except for respiration, was assessed for the pre-tone and each post-tone period on day 2.

Barnes maze

The Barnes maze was performed as described (14, 15). The Barnes maze is a circular platform (~1.2 m in diameter and 0.9 m above floor level) containing 20 holes of 5 cm diameter spaced evenly around the periphery. One of these holes (target) allows access to a small, dark chamber into which a test mouse can descend. To encourage mice to enter the target hole the light intensity was increased to 200 lux and noise level increased to ~85 decibel. Visual cues were placed surrounding the maze.

During the training phase, mice were placed in a wire cage in the centre of the platform. After 10 sec the cage was removed, and mice allowed to freely explore the maze for a maximum of 4 min. If a mouse did not enter the target hole within this period it was gently encouraged to

do so. Once inside the target chamber mice were allowed to remain for 2 min. Mice received 4 trials per day for 4 days, with a 15 min break between trials. On day 5, 24 h after the final training day, the target hole was closed. Mice were allowed to freely explore the maze for 2 minutes. The number of errors (nose pokes and head deflections) before finding the target and deviation (how many holes away from the target) at first error were assessed. In addition, search strategies were classified as random (exploring two or more non-consecutive holes), serial (searching clockwise or anti-clockwise with no more than one hole missed) or spatial (navigating directly to the target hole or the +1 or –1 position).

Rotor-rod test

Motor coordination and strength were measured using a rotating rod (rotor-rod; Rotamex-5, Columbus Instruments). Mice were lowered onto a 3 cm diameter rod rotating at 12, 16, 20, 25, 30, 35, 40 or 45 rpm for 2 min or until they fell. Mice were given a 5 min rest between sessions with increasing rotational speed. Two trials were performed with a 1 h break between trials. The latency to fall was recorded for each rotational speed. A 1 sec penalty was added if a mouse failed to walk in time with the rod but rather gripped the rod and rotated with it (cartwheel).

Hanging mesh

Mice were placed onto a square wire mesh and allowed to grip the mesh with all four limbs. Mice were then inverted ~30cm above a padded surface and allowed to hang until falling. The latency to fall (sec) was recorded.

Visual Cliff

24 h prior to testing mice had their whiskers trimmed to less than 5 mm. The visual cliff consisted of a clear bottomed box placed with 1/2 of its length extending over the edge of a table. On one side of this was a chequered pattern immediately under the clear acrylic bottom of the box (shallow side). On the other side was a 'visual cliff' with the acrylic bottom suspended above a chequered pattern beneath a vertical drop (deep side). Mice were placed on the shallow side of the box, farthest from the cliff and allowed to freely explore for 5 min. The proportion of time on the shallow side was assessed and compared to a theoretic proportion of 0.5, which would indicate indiscriminate movement between the shallow and deep areas.

Home cage analysis

A home cage analysis was performed using an automated home cage analyser equipped with microchip detectors at the bottom and a camera (Actual Analytics). Microchips were inserted subcutaneously in the lower abdominal region under general anaesthesia 1 week prior to testing. Cohorts of 2 to 4 mice that had been previously housed together, were analysed over a 124 h period (16).

Buffers and Antibodies

Buffers and antibodies can be found in Supplemental Tables 10 and 11.

Peripheral blood intracellular flow cytometry

200 µl of peripheral blood were collected from mice in EDTA tubes. White blood cell counts (WBCB) were determined using a haematology analyser (ADVIA 2120i; Siemens Healthineers). Red blood cells were depleted using a hypotonic RBCB buffer. Dead cells were stained using a LIVE/DEAD Fixable stain (L23101, ThermoFisher) in PBS for 30 min on ice.

Cells were washed in PBS and centrifuged (200 *g*, 5 min). Cells were resuspended in 2% FACS buffer containing cell surface antibodies for 1 h on ice. Cells were washed in 2% FACS buffer and centrifuged (200 g, 5 min) before being fixed and permeabilised using the FOXP3 transcription factor kit (00-5523-00, ThermoFisher) for 1 h on ice. Cells were washed using 2% FACS buffer for 1 h to remove the fixation buffer. Cells were resuspended in 2% FACS buffer containing a primary histone acetylation antibody and incubated O/N at 4°C on a roller. The following morning the cells were washed using 2% FACS buffer and a secondary antibody added for 1 h on ice. Samples were analysed using a flow cytometry analyser (FACS LSR™ II SORP or FortessaX20, BD Biosciences) at a rate of less than 7,500 events per second.

Acid protein (histone) extraction from adult cortex or HEK293T cells

Cortices were dissected and snap frozen. Frozen tissue was pulverised using a hammer/metal tool. Pulverised tissue was collected into a 1.5 ml Eppendorf tube.

HEK293T cells were grown in DMEM + 10% FBS + penicillin/streptomycin in 6-well cell culture dishes. Cells were washed twice in PBS containing 2.2 mg/ml sodium butyrate to inhibit histone deacetylases and 1x EDTA-free protease inhibitor tablet (5056489001, Sigma) before being scraped and collected.

Histones were extracted using one of two methods:

(i) Pulverised cortices were lysed using histone acid buffer containing 2.2 mg/ml sodium butyrate and 1x EDTA-free protease inhibitor tablet (5056489001, Sigma) for 30 min on a roller. Nuclei were pelleted (11000 *g*, 10 min) and resuspended in 400 µl 0.2 M H_2SO_4 and incubated on ice for 2 h. Tissue debris was removed by centrifugation (11000 *g*, 10 min) and supernatant transferred to dialysis tubing (08-607, Spectrum™). Samples were dialysed against

acetic acid for 1 h at 4°C followed by dialysis against MQ-H_2O O/N. The following morning samples were collected, and protein quantitated using a BCA assay.

(ii) Alternative, HEK293T cells were lysed and histones extracted using TCA as described (17).

Western immunoblotting

0.2 - 2 μg adult cortex-derived or HEK293T-derived histones were separated on a 4-12% Bis-Tris gel (NP0321, ThermoFisher) in MES-running buffer and transferred onto a nitrocellulose membrane (926-31902, Li-Cor). Membranes were blocked in Odyssey blocking buffer (927-50000, Li-Cor) for 1 h at RT and probed with an acetylation antibody and pan H3 loading control, O/N at 4°C on a roller. Membranes were washed in PBST (PBS + 0.1% Tween-20) and incubated with Odyssey IRDye® anti-rabbit 800 (926-32211, Li-Cor) and Odyssey IRDye® anti-mouse 680 (926-68070, Li-Cor) for 1 hr at RT. Membranes were washed and exposed using an automated western blot densitometry system (Odyssey ® Imagers, Li-Cor).

E16.5 cortical neuron culture

Dissected E16.5 cortices were digested in 200 μl trypsin-EDTA (Sigma, 1006132) for 10 min in a 37°C water bath. Excess trypsin was removed and replaced with 1 ml cortical neuron medium (DMEM/F12 (Gibco, 12500-062), 15.8 mM D-glucose (Sigma, G7021), 5 mM HEPES (Sigma, H-4034), 13.4 mM $NaHCO_3$ (Sigma, G-7021), 100 U/ml penicillin-streptomycin (Gibco, 15140-122), 25 μg/ml insulin (Sigma, I-6634), 60 μM putrescine dihydrochloride (Sigma, P-5780), 100 μg/ml apo-transferrin (Sigma, T-2252), 30 nM selenium sodium salt (Sigma S-9133), 20 nM progesterone (Sigma, P-6149), 0.2% BSA (Sigma, A-3311), 1% FCS)(18). Cells were gently triturated, passed through a 100 μm cell sieve (Corning, CLS431752) and pelleted (200 *g*, 5 min). Cells were counted using an automated cell counter

(Countess^{TM}, ThermoFisher) and plated onto 12-well plates (Nunc, 150628) or chamber slides (Ibidi, 80826) pre-coated overnight with 0.1% poly-D-lysine (Sigma, P6403). Cells were grown at 37°C in 5% CO_2.

RT-qPCR

RNA was isolated from HEK293T cells or mouse adult cortex using an RNA extraction kit (RNeasy Mini, Qiagen, 74106). RNA was collected from E16.5 cortical neurons by storing cells in DNA/RNA Shield (Zymo, R1100), followed by RNA extraction using a kit (RNeasy Micro, Qiagen, 74004) according to the manufacturer's instructions and including the optional DNase I digest step. RNA was quantitated using a spectrophotometer/fluorometer (Denovix, DS-11 series) or high sensitivity RNA tape (Agilent, 5067-5579 and 4200 Tapestation. Agilent) and 1 μg RNA was used for cDNA synthesis using a cDNA synthesis kit (SuperScript IV cDNA Synthesis Kit, ThermoFisher, 18091050) according to the manufacturer's instructions. cDNA was amplified using a real-time PCR machine (QuantStudio, ThermoFisher) with primers shown in Supplemental Table 9.

RNA sequencing

RNA sequencing was performed on cultured cortical neurons isolated from E16.5 *Kat6b*$^{+/+}$and *Kat6b*$^{+/–}$ foetuses, isolated and cultured as described in the *E16.5 cortical neuron culture* section above. Cells were treated with Vehicle (untreated medium), 1 mM VPA or 1 mM ALCAR for 4 days, with daily medium changes. For RNA isolation, medium was removed and replaced with 200 μl DNA/RNA Shield (Zymo, R1100) followed by RNA extraction using a kit (RNeasy micro, Qiagen, 74004) according to the manufacturer's instructions and including the optional DNase I digest. RNA quantity and quality was assessed using a high sensitivity RNA tape (Agilent, 5067-5579 and 4200 Tapestation, Agilent). 100 ng RNA was used to

generate RNA libraries using a sequencing library construction kit (TruSeq RNA Library Prep Kit v2, Illumina) according to the manufacturer's instructions. Libraries were sequenced on a NextSeq2000 instrument (Illumina) to give ~22 million reads per sample.

RNA sequencing data analysis

Paired-end FastQ files were aligned to the *Mus musculus* genome (build mm39) using Rsubread (v2.12.3) (19). In all cases at least 98% of fragments were successfully mapped to the genome. Fragments overlapping genes were then summarized using Rsubread's featureCounts function. Genes were identified by Rsubread's inbuilt RefSeq annotation to the mm39 genome. Differential expression analyses were then carried out using limma (v3.56.4) (20) and edgeR (v3.42.4) (21).

Prior to analysis, all sex specific genes (*Xist* and those unique to the Y chromosome) were removed to avoid sex biases. Additionally, all genes without an official gene symbol, non-protein coding genes, and Riken genes were also removed. It should be noted that all samples were sequenced over two runs. Similarity between the two runs was examined using a multi-dimensional scaling (MDS) plot. It was found that the second technical replicate of one of the ALCAR treated *Kat6b* wild type (WT) samples was of poor quality and removed from analysis. All other technical replicates were combined to form single samples using limma's sumTechReps function. Independent analyses were then conducted on the ALCAR/Vehicle and VPA/Vehicle treated samples.

In both cases, the same analytical pipeline was applied. Expression based filtered was performed using edgeR's filterByExpr function with default parameters. A total of 14,199 genes remained in the ALCAR/Vehicle analysis, while 14,025 remained the VPA/Vehicle analysis. Sample composition was then normalized in each case using the TMM method (22). The data was then transformed to log_2CPM with associated precision weights using voom (23).

Correlation between samples from the mouse was then estimated using limma's duplicateCorrelation function (24). Following this, the data was re-transformed to log_2CPM using voom to incorporate the correlation estimate. Differential expression between groups was then assessed using linear models and robust empirical bayes moderated t-statistics. The linear models incorporated the correlation estimate and mouse blocking factor (robust limma-voom pipeline with duplicate correlation estimate).

In both cases the false discovery fate (FDR) was controlled below 5% using the Benjamini and Hochberg method. Pathway analyses of the Gene Ontology (GO) and KEGG databases was performed using limma's goana and kegga functions respectively. Analyses of the Molecular Signatures Database was achieved using limma's fry function. All MDS and mean-difference (MD) plots were drawn using limma's plotMDS and plotMD functions respectively. All data for heatmaps was row scaled using the pheatmap package, and heatmaps drawn using the ComplexHeatmap package.

Mitochondrial ToxGlow assay of ATP output

SBBYSS HEK293T cells and E16.5 cortical neurons were cultured as describe above. HEK293T cells were grown in DMEM (Gibco, 12320032), 10 U/ml penicillin/streptomycin (Gibco, 15140122), 1x GlutaMax™ (35050061), 1x MEM Non-Essential Amino Acids Solution (Gibco, 11140050), 1 mM HEPES (Sigma, H-4034 and 10% dialysed FBS (Gibco, 26400-044) with either 25 mM D-glucose (Sigma, G8644) or 25 mM D-galactose (Gibco, 11966025) for 48 h prior to testing. Cortical neurons were cultured in cortical neuron medium with 15.8 mM glucose and 1 % FBS or with 15.8 mM galactose and 1% dialysed FBS 24 h prior to testing. ATP levels were assayed using a kit (Mitochondrial ToxGlo™ assay, Promega, G8000) according to the manufacturer's instructions. Luminescence was assessed using a microplate reader (Clariostar, BMG Labtech).

Mitochondrial MitoTracker staining and live cell imaging

SBBYSS HEK293T cells or E16.5 cortical neurons were cultured on 8-well chamber slides (Ibidi, 80826) pre-coated with 0.1 % (w/v) poly-D-lysine (Sigma, P7405) in respective galactose-based medium, as described in the *Mitochondrial ToxGlow assay of ATP output* section above. The mitochondrial marker dye MitoTracker DeepRed (ThermoFisher, M22426) was added to all samples at to a final concentration of 0.1 - 0.2 nM, for 30 min at 37°C. Cells were washed twice in fresh medium, and images were acquired using a confocal microscope (Stellaris 8, Leica). Images were collected using a 40 x 1.3 NA HC PL APO CS2 oil immersion objective with a voxel size consistently set to 100 x 100 x 346 nm independent of the overall zoom factor. A super continuum white light laser source was set to 641 nm for the optimal excitation of the MitoTracker DeepRed and the resultant fluorescence collected via a detector (HyD X, Leica) running in analogue mode with a gain of 2.5 across a band extending from 651 to 750 nm. Images were collected with a depth of 16-bits with 2 times line averaging to minimise noise. Three to five random positions across a monolayer of cells were chosen for each condition. Acquired Z-stacks were deconvolved using Huygens deconvolution software (Professional Version; Version 21.10.0p0 64b) and deconvolved stacks were analysed using image analysis software (Fiji2, Version 2.14.0/1.54f) (25) using the MorphLibJ plugin, as described (26). Average branch number and length of skeletonised Z-projections were assessed as readouts of mitochondrial morphology.

Cresyl Violet staining of adult brains

Adult mice were fixed by transcardial perfusion with PBS followed by 4 % PFA. Brains were dissected and postfixed in 4% PFA for 24 h on a roller at 4°C. Brains were paraffin embedded

and 7 μm coronal sections stained for cresyl violet. Volumetric analysis was carried out as described in (27, 28).

References

1. Wichmann J, Pitt C, Eccles S, Garnham AL, Li-Wai-Suen CSN, May R, et al. Loss of TIP60 (KAT5) abolishes H2AZ lysine 7 acetylation and causes p53, INK4A, and ARF-independent cell cycle arrest. *Cell Death Dis.* 2022;13(7):627.
2. Jacobi AM, Rettig GR, Turk R, Collingwood MA, Zeiner SA, Quadros RM, et al. Simplified CRISPR tools for efficient genome editing and streamlined protocols for their delivery into mammalian cells and mouse zygotes. *Methods.* 2017;121-122:16-28.
3. Aubrey BJ, Kelly GL, Kueh AJ, Brennan MS, O'Connor L, Milla L, et al. An inducible lentiviral guide RNA platform enables the identification of tumor-essential genes and tumor-promoting mutations in vivo. *Cell Rep.* 2015;10(8):1422-32.
4. Schwenk F, Baron U, and Rajewsky K. A cre-transgenic mouse strain for the ubiquitous deletion of loxP-flanked gene segments including deletion in germ cells. *Nucleic Acids Res.* 1995;23(24):5080-1.
5. Heyser CJ. Assessment of developmental milestones in rodents. *Curr Protoc Neurosci.* 2004;Chapter 8:Unit 8 18.
6. Samson AL, Ju L, Ah Kim H, Zhang SR, Lee JA, Sturgeon SA, et al. MouseMove: an open source program for semi-automated analysis of movement and cognitive testing in rodents. *Sci Rep.* 2015;5:16171.
7. Leger M, Quiedeville A, Bouet V, Haelewyn B, Boulouard M, Schumann-Bard P, et al. Object recognition test in mice. *Nat Protoc.* 2013;8(12):2531-7.
8. Bevins RA, and Besheer J. Object recognition in rats and mice: a one-trial non-matching-to-sample learning task to study 'recognition memory'. *Nat Protoc.* 2006;1(3):1306-11.
9. Ennaceur A, and Delacour J. A new one-trial test for neurobiological studies of memory in rats. 1: Behavioral data. *Behav Brain Res.* 1988;31(1):47-59.
10. Kraeuter AK, Guest PC, and Sarnyai Z. Neuropsychiatric Sequelae of Early Nutritional Modifications: A Beginner's Guide to Behavioral Analysis. *Methods Mol Biol.* 2018;1735:403-20.
11. Tucker LB, and McCabe JT. Behavior of Male and Female C57BL/6J Mice Is More Consistent with Repeated Trials in the Elevated Zero Maze than in the Elevated Plus Maze. *Front Behav Neurosci.* 2017;11:13.
12. Kaidanovich-Beilin O, Lipina T, Vukobradovic I, Roder J, and Woodgett JR. Assessment of social interaction behaviors. *J Vis Exp.* 2011(48).
13. Curzon PR, N.R & Browman, K.E. CRC Press/Taylor & Francis; 2011:Section 2.5.1-2.5.2.
14. Mayford M, Bach ME, Huang YY, Wang L, Hawkins RD, and Kandel ER. Control of memory formation through regulated expression of a CaMKII transgene. *Science.* 1996;274(5293):1678-83.

15. Bach ME, Hawkins RD, Osman M, Kandel ER, and Mayford M. Impairment of spatial but not contextual memory in CaMKII mutant mice with a selective loss of hippocampal LTP in the range of the theta frequency. *Cell.* 1995;81(6):905-15.
16. Armstrong JD, Acevedo-Arozena A, Bains RS, Cater H, Chartsias A, Nolan P, et al. Tracking of Individual Mice in a Social Setting Using Video Tracking Combined with RFID tags. *Proc Measuring Behav.* 2016;10:413-6.
17. Sidoli S, Bhanu NV, Karch KR, Wang X, and Garcia BA. Complete Workflow for Analysis of Histone Post-translational Modifications Using Bottom-up Mass Spectrometry: From Histone Extraction to Data Analysis. *J Vis Exp.* 2016(111).
18. Merson TD, Dixon MP, Collin C, Rietze RL, Bartlett PF, Thomas T, et al. The transcriptional coactivator Querkopf controls adult neurogenesis. *J Neurosci.* 2006;26(44):11359-70.
19. Liao Y, Smyth GK, and Shi W. The R package Rsubread is easier, faster, cheaper and better for alignment and quantification of RNA sequencing reads. *Nucleic Acids Res.* 2019;47(8):e47.
20. Ritchie ME, Phipson B, Wu D, Hu Y, Law CW, Shi W, et al. limma powers differential expression analyses for RNA-sequencing and microarray studies. *Nucleic Acids Res.* 2015;43(7):e47.
21. Robinson MD, McCarthy DJ, and Smyth GK. edgeR: a Bioconductor package for differential expression analysis of digital gene expression data. *Bioinformatics.* 2010;26(1):139-40.
22. Robinson MD, and Oshlack A. A scaling normalization method for differential expression analysis of RNA-seq data. *Genome Biol.* 2010;11(3):R25.
23. Law CW, Chen Y, Shi W, and Smyth GK. voom: Precision weights unlock linear model analysis tools for RNA-seq read counts. *Genome Biol.* 2014;15(2):R29.
24. Smyth GK, Michaud J, and Scott HS. Use of within-array replicate spots for assessing differential expression in microarray experiments. *Bioinformatics.* 2005;21(9):2067-75.
25. Schindelin J, Arganda-Carreras I, Frise E, Kaynig V, Longair M, Pietzsch T, et al. Fiji: an open-source platform for biological-image analysis. *Nat Methods.* 2012;9(7):676-82.
26. Chaudhry A, Shi R, and Luciani DS. A pipeline for multidimensional confocal analysis of mitochondrial morphology, function, and dynamics in pancreatic beta-cells. *Am J Physiol Endocrinol Metab.* 2020;318(2):E87-E101.
27. Coggeshall RE. A consideration of neural counting methods. *Trends Neurosci.* 1992;15(1):9-13.
28. West MJ, Slomianka L, and Gundersen HJ. Unbiased stereological estimation of the total number of neurons in thesubdivisions of the rat hippocampus using the optical fractionator. *Anat Rec.* 1991;231(4):482-97.
