# Supplementary material for: Increasing histone acetylation improves sociability and restores learning and memory in KAT6B-haploinsufficient mice
Source: J Clin Invest. 2024 Apr 1;134(7):e167672. doi: 10.1172/JCI167672 (PMC10977983; doi:10.1172/JCI167672)

Figure 2

Figure 2C

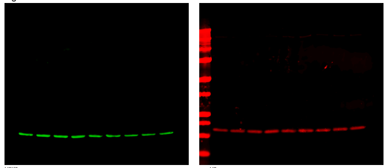

H3K9ac pan H3

Figure 2E

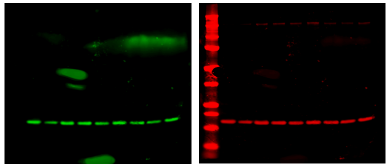

H3K23ac pan H3

Figure 2G

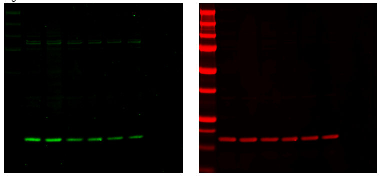

H3K9ac pan H3

Figure 2H

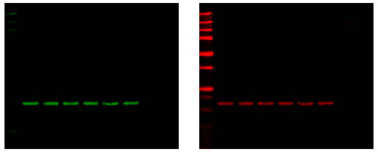

H3K9ac pan H3

Figure 7

Figure 7D

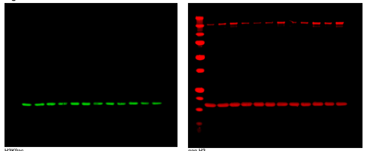

H3K9ac pan H3

Figure 7F

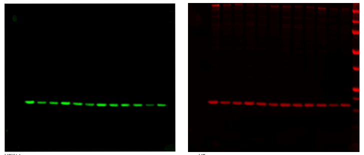

H3K14ac pan H3

Figure 7H

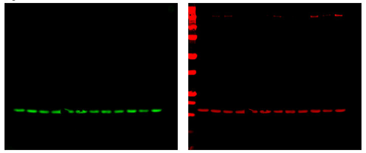

H3K23ac pan H3

Figure 10

Figure 10D

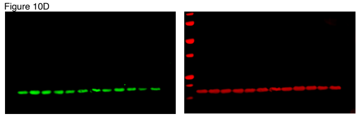

H3K9ac pan H3

Figure 10F

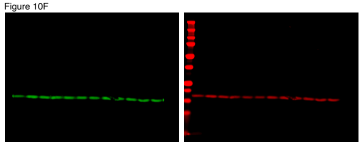

H3K14ac pan H3

Figure 10H

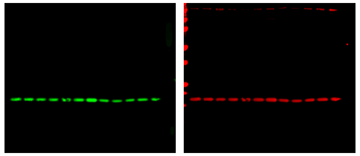

H3K23ac pan H3

Supplemental Figure 1

Supplemental Figure 1A

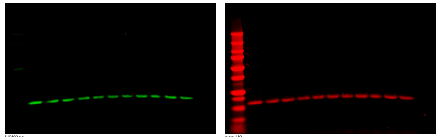

H3K9ac pan H3

Supplemental Figure 1C

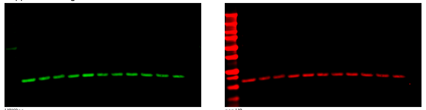

H3K9ac pan H3

Supplemental Figure 1E

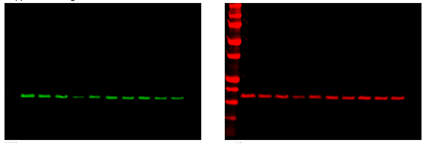

H3K9ac pan H3

Supplemental Figure 1G

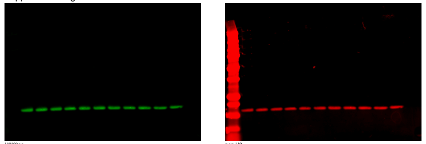

H3K9ac pan H3

Supplemental Figure 2

Supplemental Figure 2A

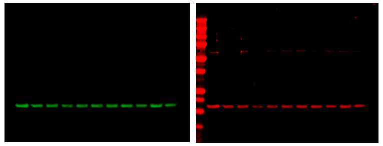

H3K23ac pan H3

Supplemental Figure 2C

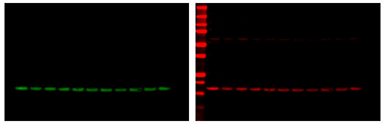

H3K23ac pan H3

Supplemental Figure 2E

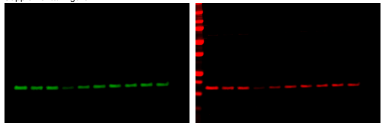

H3K23ac pan H3

Supplemental Figure 2G

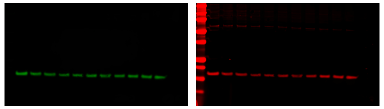

H3K23ac pan H3

Supplemental Figure 3

Supplemental Figure 3A

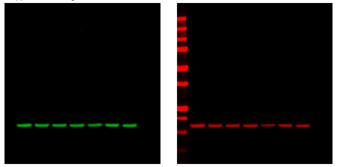

H3K14ac pan H3

Supplemental Figure 3C

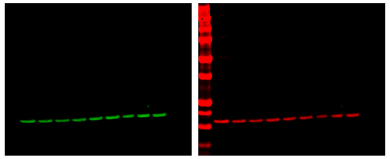

H3K14ac pan H3

Supplemental Figure 3E

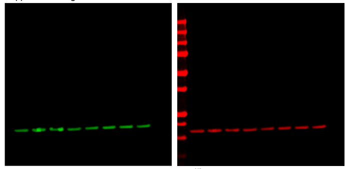

H3K14ac pan H3

Supplemental Figure 3G

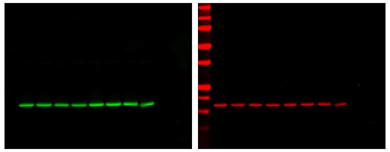

H3K14ac pan H3

Supplemental Figure 3I

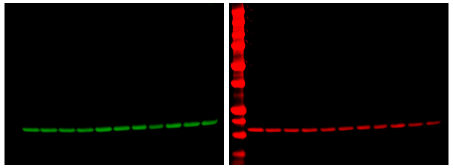

H3K14ac pan H3

Supplemental Figure 3K

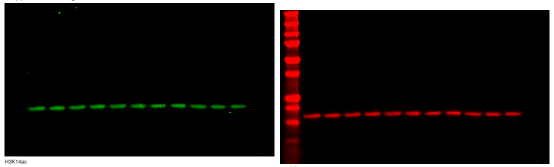

H3K14ac pan H3

Supplemental Figure 6A

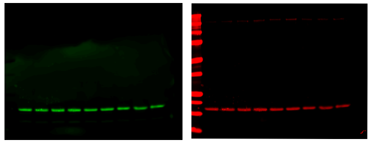

H3K14ac pan H3

Supplemental Figure 12A

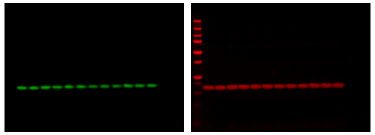

H3K9ac pan H3

Supplemental Figure 16A

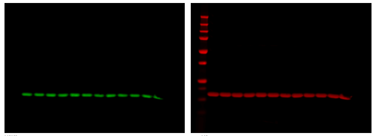

H3K9ac pan H3

Supplemental Figure 13 A

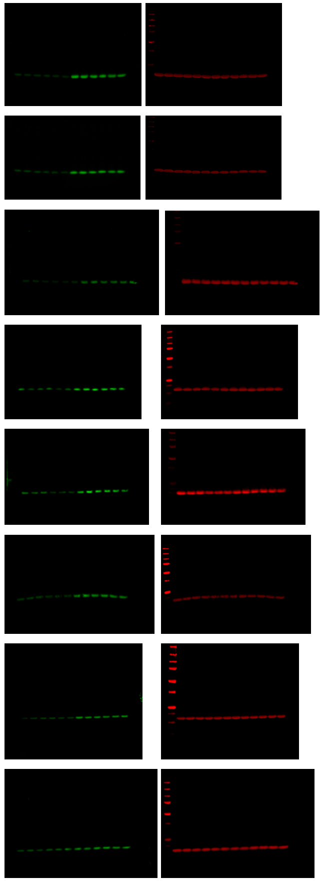

Supplemental Figure 13B

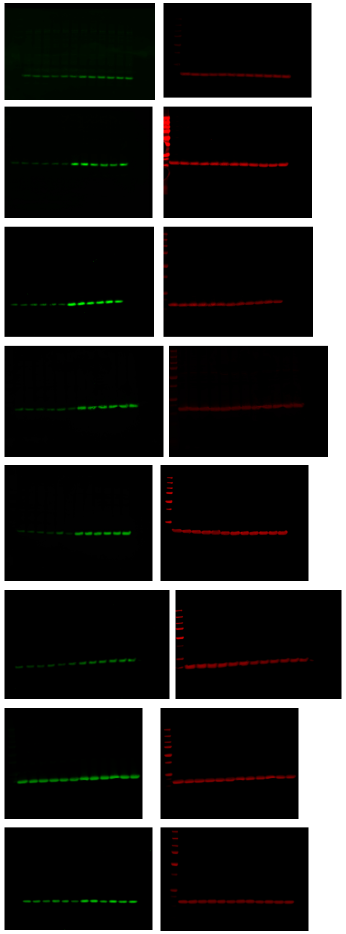

Supplemental Figure 13C

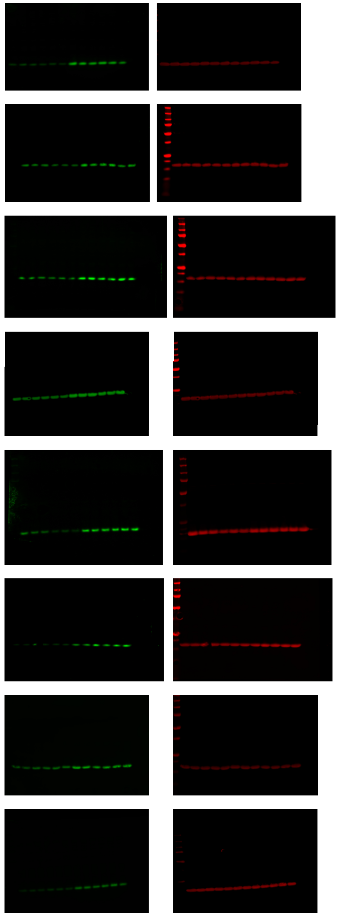

Supplemental Figure 15A

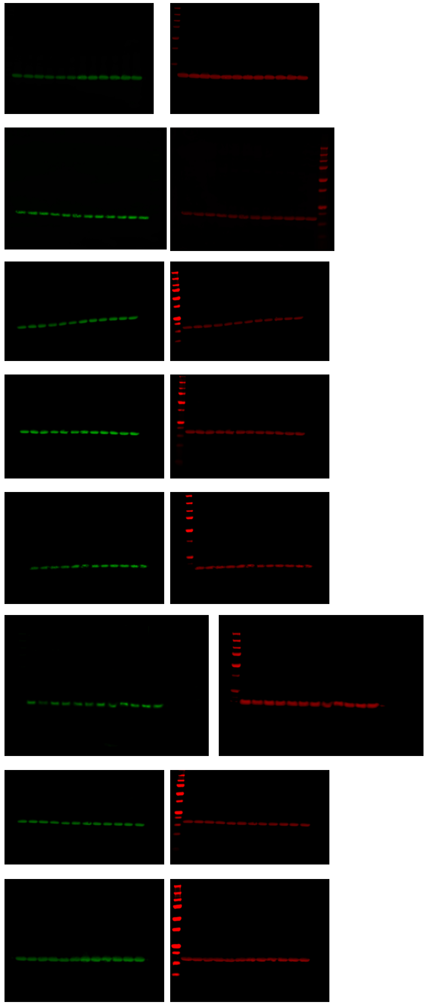

Supplemental Figure 15B

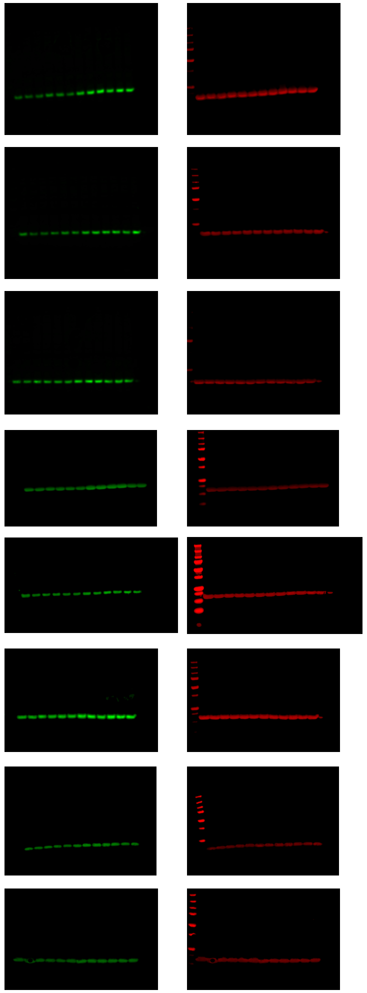

Supplemental Figure 15C

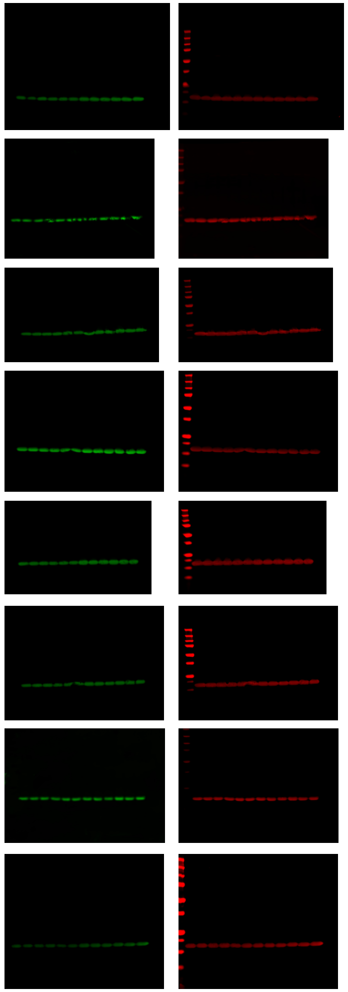

Supplement: Unedited blot and gel images [file jci-134-167672-s088.pdf]
